# Changes in cortical gene expression in the muscarinic M1 receptor knockout mouse: potential relevance to schizophrenia, Alzheimer’s disease and cognition

**DOI:** 10.1038/s41537-021-00174-z

**Published:** 2021-09-14

**Authors:** Brian Dean, Elizabeth Scarr

**Affiliations:** 1 The Molecular Psychiatry Laboratory, The Florey Institute for Neuroscience and Mental Health, Parkville, VIC Australia; 2 Melbourne Veterinary School, Faculty of Veterinary and Agricultural Sciences, The University of Melbourne, Parkville, VIC Australia

**Keywords:** Schizophrenia, Molecular neuroscience

## Abstract

Postmortem and neuroimaging studies show low levels of cortical muscarinic M1 receptors (CHRM1) in patients with schizophrenia which is significant because CHRM signalling has been shown to change levels of gene expression and cortical gene expression is altered in schizophrenia. We decided to identify CHRM1-mediated changes in cortical gene expression by measuring levels of RNA in the cortex of the Chrm1^−/−^ mouse (*n* = 10), where there would be no signalling by that receptor, and in wild type mouse (*n* = 10) using the Affymetrix Mouse Exon 1.0 ST Array. We detected RNA for 15,501 annotated genes and noncoding RNA of which 1,467 RNAs were higher and 229 RNAs lower in the cortex of the Chrm1^−^^/−^ mouse. Pathways and proteins affected by the changes in cortical gene expression in the Chrm1^−/−^ are linked to the molecular pathology of schizophrenia. Our human cortical gene expression data showed 47 genes had altered expression in Chrm1^−/−^ mouse and the frontal pole from patients with schizophrenia with the change in expression of 44 genes being in opposite directions. In addition, genes with altered levels of expression in the Chrm1^−^^/−^ mouse have been shown to affect amyloid precursor protein processing which is associated with the pathophysiology of Alzheimer’s disease, and 69 genes with altered expression in the Chrm1^−^^/−^ mouse are risk genes associated with human cognitive ability. Our findings argue CHRM1-mediated changes in gene expression are relevant to the pathophysiologies of schizophrenia and Alzheimer’s disease and the maintenance of cognitive ability in humans.

## Introduction

Schizophrenia is characterised by the onset of positive (e.g., delusions and hallucinations), negative (e.g., apathy and anhedonia), and cognitive symptoms^1^. Whilst the molecular mechanisms causing schizophrenia are unknown, it is clear a dysfunctional prefrontal cortex contributes to the pathophysiology of the disorder^2^ and that schizophrenia occurs in individuals with a genetic susceptibility due to the inheritance of risk genes who encounter one or more deleterious environmental factors^3^. Importantly, both inherited risk genes^4^ and environmental factors, via epigenetic mechanisms^3^, act to change levels of gene transcription, and thus changes in levels of RNA in the prefrontal cortex from patients with schizophrenia are likely contributing to the molecular pathology of the disorder^5^.

One finding supported by human postmortem and neuroimaging studies is that there are lower levels of muscarinic M1 receptors (CHRM1) in the cortex of patients with schizophrenia^6^ and that the cognitive deficits experienced by patients with schizophrenia are related to the levels of that receptor^7^. Importantly, our data show that levels of CHRM2−4 are not altered in the cortex of patients with schizophrenia^8,9^ and that levels of CHRM5 could not be detected (unpublished data). Significantly, signalling through CHRMs has been shown to affect gene expression^10^ which argues that changes in cortical CHRM1-regulated gene expression must be contributing to the molecular pathology of schizophrenia. Moreover, it has been recently reported that a co-formulation of the selective M1/M4 agonist, xanomeline, and the peripheral muscarinic receptor antagonist, trospium, reduces both the positive and negative symptoms of schizophrenia^11^. This raises the possibility that changes in CHRM1 receptor-mediated gene expression may be an important mechanism of action of drugs that can reduce symptom severity in those with the disorder.

The development of Chrm1−5^−^^/−^ mice has provided useful tools for the study of the physiological roles of those receptors in mammalian CNS^12^. At the behavioural level, Chrm1^−/−^ mice showed no deficits in sensory-motor gating, nociception, motor coordination, anxiety-related behaviour, or hippocampal learning and memory^13^. By contrast, Chrm1^−^^/−^ mice were severely impaired in non-matching-to-sample working memory^14^, which needs cortical and hippocampal engagement. Hence, it would appear that the cortical Chrm1 modulates behaviours involving cortical to sub-cortical communication which is significant because patients with schizophrenia have a loss of CHRM1 positive pyramidal neurons in cortical laminae V, neurons which are critical in cortical-subcortical communication^15^.

Using HEK293 cells, it has been shown that CHRM1 signalling affects gene expression^10^. We have shown there are lower levels of cortical CHRM1^8^ and changes in gene expression in the cortex from patients with schizophrenia^16–18^. Based on these data, we postulated that some changes in cortical gene expression in patients with schizophrenia would be associated with lower levels of CHRM1 signalling. Thus, we decided to compare cortical gene expression in Chrm1^−^^/−^ to wild type (w/t) mice using the Mouse Exon 1.0 ST Array because that array is the mouse equivalent of the Human Exon 1.0 ST Array which we used to measure gene expression in the human cortex. Using analogous technology allowed us to better compare changes in gene expression in the cortex from Chrm1^−^^/−^ mice and patients with schizophrenia. In addition, because the CHRM1 is important in cognition^19^ we wanted to identify genes with changed levels of expression in the Chrm1^−^^/−^ mice that, using gene-wide association studies (GWAS), had been associated with human cognitive ability^20^.

## Results

### Changes in cortical gene expression in Chrm1^−^^/−^ mice

**Fig. 1:**
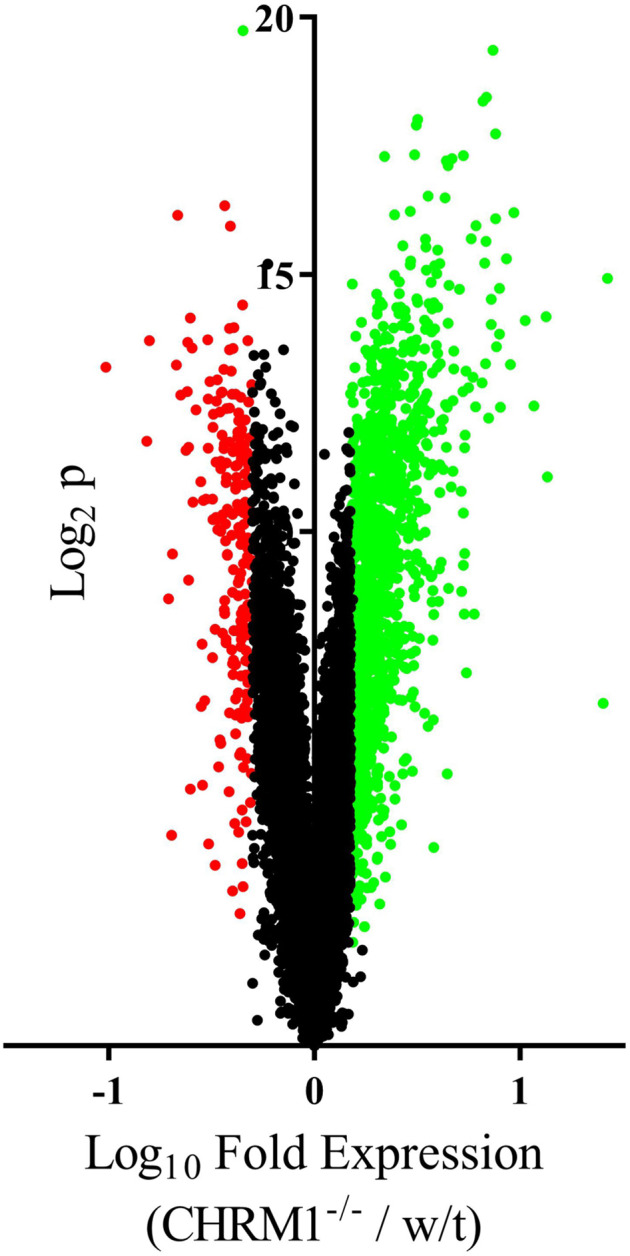
Levels of gene expression in the cortex of the Chrm1^−^^/^^−^ compared to that in wild type mice. Volcano plot showing the levels of expression of 15,501 genes in the cortex of Chrm1^−^^/^^−^ compared to that in wild type (w/t) mice. Green dots = increased gene expression, red dots = decreased gene expression, black dots unchanged gene expression.

Levels of RNA for 15,501 annotated genes and noncoding RNA were detectable in the cortex from Chrm1^−^^/−^ and w/t mice of which, levels of 1,467 RNAs were higher and 229 RNAs were lower in the Chrm1^−^^/−^ mice (Fig. 1; Supplementary Table 1). Notably, the signal for mRNA from the Chrm1 gene was essentially equal to the background, giving significant face validity to our analyses.

### Impact of changed gene expression on Chrm1^−^^/^^−^ cortical function

**Fig. 2:**
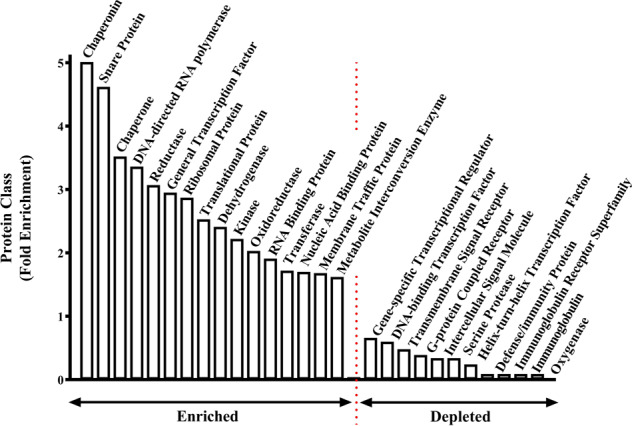
Protein classes affected by changes in gene expression in the Chrm1^−^^/^^−^ mouse. Protein classes containing enriched or depleted levels of genes with altered levels of expression in the cortex of Chrm1^−^^/^^−^ mouse.

The Panther Gene Ontology Classification System predicted changes in gene expression would impact on 198 distinct biological processes in the cortex of Chrm1^−^^/^^−^ mice (Supplementary Table 2). These changes in biological processes would likely be driven by the enrichment and depletion of specific classes of proteins in the cortex of Chrm1^−^^/^^−^ mice (Fig. 2; Supplementary Table 3) especially as some of these protein classes have well-defined roles in CNS function.

**Fig. 3:**
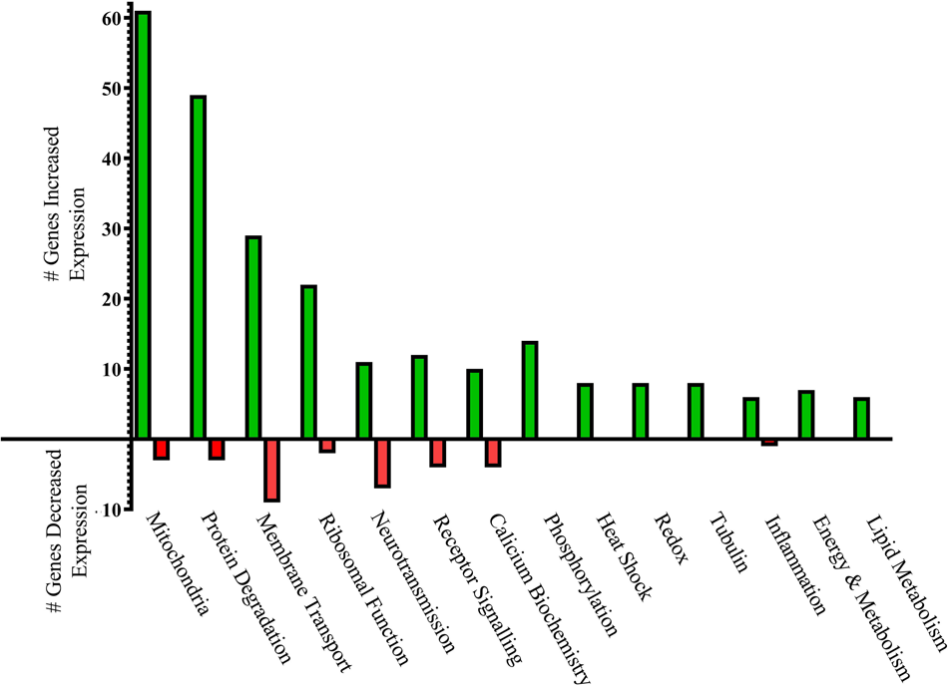
Cellular functions affected by changes in cortical gene expression in the Chrm1^−^^/−^ mouse. The number of coding and noncoding RNA that are known to be associated with specific cellular functions that had either higher (green columns) or lower (red columns) levels on the cortex of Chrm1^−^^/−^ mouse.

We also completed a manual curation of the functional role of genes with altered levels of expression in the cortex of Chrm1^−^^/^^−^ mice to identify groupings of genes that could be clustered within specific cellular functions. This analysis showed that genes involved in mitochondrial function, protein degradation, membrane transport, neurotransmission, receptor signalling, ribosomal function, calcium biochemistry, phosphorylation, heat shock, Redox reactions, tubulin biochemistry, inflammation, energy, and metabolism, as well as lipid metabolism had altered levels of expression in the cortex of the Chrm1^−^^/^^−^ mouse (Fig. 3 and Supplementary Table 4).

### Changes in cortical gene expression in Chrm1^−^^/−^ mice and in patients with schizophrenia

One of the strengths of our study design is we measured levels of gene expression in the mouse cortex with a GeneChip™ Mouse Exon 1.0 ST Array, which is comparable to the GeneChip™ Human Exon 1.0 ST Array used in our study comparing gene expression in three cortical regions from patients with schizophrenia to that in age-matched controls^18^. These two studies showed there were 47 genes with changed levels of expression in both the cortex of Chrm1^−^^/−^ mice and Brodmann’s (BA) 10 from patients with schizophrenia^18^ (Supplementary Table 5). By contrast, there were no genes that had changed levels of expression in the cortex of Chrm1^−^^/−^ mice and BA 9 or BA 33 from patients with schizophrenia.

### Changes in cortical gene expression in Chrm1^−^^/−^ mice: relevance to the human cognition GWAS

As the Chrm1^−^^/−^ mice had some aberrations in the cognitive function we postulated some changes in cortical gene expression could be associated with cognition that could be relevant to humans. It was therefore notable that there were changes in the levels of expression of 69 genes reported as being associated with variation in human cognition in the cortex of the Chrm1^−^^/−^ mouse (Supplementary Table 6). As cognitive deficits are a symptom of schizophrenia we also compared changes in gene expression in BA 10 from patients with schizophrenia to genes associated with variation in human cognitive ability and found 12 genes with altered levels of expression in BA 10 from patients with schizophrenia that have been associated with varying cognitive ability in humans (Supplementary Table 7). In addition, there were two genes (AKT interacting protein (Aktip) and nuclear receptor subfamily 1, group D, member 2 (Nrld2) which had changed levels of expression in the cortex of Chrm1^−^^/−^ mice and BA 10 from people with schizophrenia that have also been shown to be associated with varying cognitive ability in humans.

## Discussion

Here we show Chrm1 signalling in mouse CNS is associated with changes in cortical gene expression. Changes in gene expression are mostly higher in the Chrm1^−^^/−^ mouse, thus our data argues Chrm1 signalling predominantly acts to suppress cortical gene expression. Changes in cortical gene expression in the Chrm1^−^^/−^ mouse include the expression of enzymes, SNARE proteins, chaperone proteins, as well as proteins involved in receptor signalling, gene transcription and translation, metabolism, and immunity. Such changes in cortical gene expression would impact on mitochondrial function, protein translation and degradation, membrane transport, neurotransmission, receptor signalling, and calcium biochemistry, which are critical to maintaining CNS function. One limitation of transcriptomic studies is that changes in coding RNA (i.e., gene expression) can be directly, inversely, or not related to changes in levels of protein encoded by messenger RNA^21^ whilst changes in non-coding RNA can activate or inhibit gene expression^22^. Hence, understanding the impact of changes in the cortical transcriptome of the Chrm1^−^^/−^ mouse on the cortical proteome will be required to better understand the changes in the activity of affected biochemical pathways.

Behaviourally, the Chrm1^−^^/−^ mouse does not have seizures after exposure to pilocarpine but does have increased locomotor activity, mild and selective cognitive impairments, and a number of electrophysiological deficits^23^. Importantly, the Chrm1^−^^/−^ mouse has impairments in nonmatching-to-sample working memory and consolidation, a behaviour requiring cortical interactions with the hippocampus^14^. At the biochemical level, Chrm1 agonist-stimulated activation of the mitogen-activated protein kinase (MAPK) pathway is essentially abolished in primary cortical cell cultures isolated from newborn Chrm1^−^^/−^ mice^24^. Interestingly, our data shows the expression of two MAPKs (Mapk8 and Mapk10) were higher in the cortex of Chrm1^−^^/−^ mice which could be a compensatory response to decrease signalling through the Chrm1. In addition, Chrm1 agonist-stimulated phosphatidyl inositol (PI) hydrolysis is reduced by >60% in primary cortical cell cultures from the Chrm1^−^^/−^ mouse^24^. Our study showed there was a higher expression of four enzymes critical to the functioning of the PI hydrolysis (Pi4k2a, Pip5k1a, Pik3r3, and Ip6k1). It has also been shown that pan-CHRM agonist-stimulated [35S]-GTPγS binding could not be detected in the cortex of the Chrm1^-/-^ mice^25^. Our data argue signalling via G-proteins would be further affected because of changed cortical expression of guanine nucleotide-binding proteins (Higher: Gnai3, Gnb4; Lower: Gng4, Gnao1) and regulators of G-protein signalling (Higher: Rgs4, Rgs14, Rgs17; Lower: Rgs2) in the Chrm1^−^^/−^ mouse. Our data therefore significantly increase understanding of the molecular mechanisms causing changes in Chrm1 signalling previously shown to be present in the cortex of Chrm1^−^^/−^ mice.

Primary cortical neuron cultures from the Chrm1^−^^/−^ mouse have increased production of the amyloid precursor protein (APP)^26^. It is therefore significant that our study has shown higher expression of proteins related to APP (Apbb1, Appbp2, Apbb3) in the cortex of the Chrm1^−^^/−^ mouse. Apbb1 (amyloid beta (A4) precursor protein-binding, family B, member 1) is a member of the Fe65 protein family, which interacts with APP to modulate its activity^27^. Appbp2 (amyloid-beta precursor protein (cytoplasmic tail) binding protein 2) has been shown to regulate APP protein transport and /or processing^28^. Apbb3 (amyloid beta (A4) precursor protein-binding, family B, member 3) is a protein in the cytosol that binds to the intracellular domain of APP to modulate the internalisation of that protein^29^. Thus, our data add to data suggesting Chrm1 signalling impacts on APP function, which has long been thought to be important in the pathophysiological processes underlying Alzheimer’s disease^30^.

By using homologous technologies to measure cortical gene expression, we were able to compare changes in cortical gene expression in the Chrm1^−^^/−^ mouse and BA 9, 10, and 33 from patients with schizophrenia which showed that 47 genes had altered levels of expression in the Chrm1^−^^/−^ mouse and in BA 10, but not BA 9 or 33, from patients with schizophrenia. It could be argued the comparable changes in cortical gene expression in the Chrm1^−^^/−^ and BA 10 from patients with schizophrenia were due to chance. However, the changes in gene expression in BA 9, 10, and 33 from patients with schizophrenia do not occur in the same three cortical regions from patients with bipolar disorder or major depressive disorders^31^ who do not have changes in their cortical CHRM1 levels^32,33^. These data argue that the diagnostically selective changes in gene expression present in the Chrm1^−^^/−^ and schizophrenia are likely associated with lower levels of CHRM1. Moreover, existing data implicates 26 of these genes with changed levels of expression in Chrm1^−^^/−^ mouse to the pathophysiology of schizophrenia (Supplementary Table 8), further arguing the changes in cortical gene expression in the Chrm1^−^^/−^ mouse are not purely due to chance.

It was notable that the direction of change of 44 of the 47 genes with changed levels of expression in BA 10 from patients with schizophrenia was in the opposite direction to what occurred in the cortex of the Chrm1^−^^/−^ mice. Our premise for studying the Chrm1^−^^/−^ mouse was that it was a good model in which to identify changes in cortical gene expression that would be expected in the absence of signalling by that receptor. Following that hypothesis, we would expect to have found comparable changes in the expression of some genes in the cortex of patients with schizophrenia where we have shown lower levels of CHRM18 and no changes in levels of CHRM2, 39, or 48 compared to that in controls. However, our study in the Chrm1^−^^/−^ has revealed opposing changes in levels of most of the genes that are also changed in BA 10 from patients with schizophrenia. Notably, it has been suggested that derangements of biochemical pathways from their “normal” physiological responsiveness are associated with the molecular pathology of disorders of the CNS^34^. Hence, one explanation for the opposing changes in expression of the 44 genes in the Chrm1^−^^/−^ mouse and BA 10 from patients with schizophrenia is that those genes are an important component of the pathology of the disorder.

Our gene expression studies in the cortex of the Chrm1^−^^/−^ mice and in the cortex of patients with schizophrenia have been carried out using RNA from tissue homogenates. We^15^, and others^35^, have shown there are high levels of CHRM1 on pyramidal neurons^15^ but the CHRM1 is also present on inhibitory neurons^35^, astrocytes^35^, and CNS microvascular endothelial cells^36^. Thus, at this time it is not possible to determine which cells are affected by the changes in gene expression that we have described in the Chrm1^−^^/−^ mouse and in BA 10 from patients with schizophrenia^18^.

A confound faced by all studies into the molecular pathology of schizophrenia is that the diagnosis encompasses a syndrome of disorders, which has led to the argument that the pathophysiology of schizophrenia cannot be fully elucidated until it is possible to study definable sub-groups within schizophrenia^37^. We have identified a sub-group of patients (~25%) within the syndrome of schizophrenia that can be characterised because they have markedly lower (~75%) levels of cortical CHRM1^38,39^. It would therefore be reasonable to postulate that there may be more similarities in changes in cortical gene expression in the Chrm1^−^^/−^ mouse in BA 10 from the sub-group of patients within schizophrenia and identifying such comparable changes may significantly contribute to understanding their pathophysiology. Unfortunately, our data on gene expression in BA 10 from patients with schizophrenia were from cohorts that were predominantly (80%) compose of patients with schizophrenia that do not have a marked loss of cortical CHRM1^38^.

Cognitive deficits are a core feature of both schizophrenia^40^ and Alzheimer’s disease^41^ and the CHRM1 is known to play an important role in regulating cognitive ability^7^. It is therefore of interest that 69 genes with changed levels of expression in the cortex of the Chrm1^−^^/−^ mouse have been identified by the GWAS on human cognition to be associated with varying cognitive ability^20^. Relevant to the involvement of the cortical CHRM1 and cognition, we have reported cortical CHRM1 expression varies with catechol-O-methyltransferase (COMT) genotypes that are associated with varying levels of cognitive ability but do not vary with COMT genotypes not associated with varying cognitive ability^19^. More recently, we have shown that individuals with the COMT genotypes that vary with cognitive ability have varying levels of soluble (sCOMT), but not membrane-bound, COMT in the human prefrontal cortex^42^. Importantly, sCOMT regulates the levels of catecholestrogens that, in the presence of low levels of oestrogen, bind oestrogen receptors causing them to translocate to the nucleus where they can affect gene expression by binding to oestrogen receptor elements. Pursuing that hypothesis, we have confirmed that cortical gene expression does vary between individuals with the different COMT genotypes that are associated with varying cognitive ability^43^. Relevant to our Chrm1^−^^/−^ mouse study, one of those genes (Cadm4) had a higher level of expression in the cortex of the Chrm1^−^^/−^ mouse. Whilst this suggests there is not a large overlap between CHRM1-mediated changes at the level of gene expression and changes in gene expression associated with COMT genotypes it will be important to determine if Cadm4 may be an important bridge between the two mechanisms.

In more general terms, our study suggests that the changes in gene expression in the absence of signalling through the Chrm1 would have profound effects on mitochondrial function, protein degradation, membrane transport, neurotransmission, receptor signalling, ribosomal function, calcium biochemistry, protein phosphorylation, redox state, tubulin, inflammation, as well as energy and lipid metabolism. Thus, the absence of Chrm1 signalling, at least at the level of gene expression, would be expected to have significant effects on cortical function. It is therefore significant that the Chrm1^−^^/−^ mouse did not show profound changes at the level of behaviour^23^. That been said, changed mitochondrial function, protein degradation, membrane transport, neurotransmission, receptor signalling, ribosomal function, calcium biochemistry, protein phosphorylation, redox state, tubulin, inflammation as well as energy and lipid metabolism have all been implicated in the pathophysiology of schizophrenia^18,44–50^ and in cognitive functioning^51–55^. This suggests the Chrm1^−^^/−^ mouse may be more useful in studying the molecular, rather than behavioral, changes regulated by Chrm1 signalling especially as some of these molecular changes appear to be relevant to schizophrenia^6^ and cognition^56^.

There are limitations using the Chrm1^−^^/−^ gene knockout model in studying biological systems and pathways. First, the loss of Chrm1 signalling is universal across all tissue, which may not be an issue in patients with schizophrenia as both neuroimaging^57^ and postmortem^39^ studies suggest lower levels of CHRM1 are widespread in the CNS of those with the disorder. In addition, the use of the gene knockout mouse can now be more refined as there are techniques to manipulate gene expression that allow increasing control of which cells over or under-express a gene and when the onset of either over or under expression is triggered^58^. This gives the opportunity to study the impact of changes in Chrm1 signalling within specific cell types and/or specific CNS regions and to control for when the changed level of gene expression is triggered rather than the absence of Chrm1 signalling being present from conception. This level of control could be critical in better understanding the contribution of decreased signalling through the CHRM1 to the pathophysiology in disorders such as schizophrenia which has a peak age of onset in late adolescence to early adulthood^59^. However, even without such refinements, our study is significant because it shows that the modulation of gene expression is an important mechanism of action of the Chrm1 that will be important in controlling cortical function.

## Methods

### Chrm1^−^^/−^ mice

Homozygous, inbred, specific pathogen-free breeding colonies of Chrm1^−^^/−^ mice and C57Bl/6NTac wild-type (WT) mice with the same genetic background were obtained from Taconic (Cambridge City, IN). Progeny were bred from these mice and homozygous Chrm1^−^^/−^ offspring confirmed by genotyping with male F3 progeny being used for these studies; we used male mice as in our postmortem studies nearly all cases are males. As the onset of schizophrenia in males is mainly in adolescence^60^ we used two groups (Chrm1^−^^/−^ and w/t) of 10 × 8 week old mice as they would have all reached breeding age. All mice were housed in 12 h light/dark conditions with ad libitum food and water. After cervical dislocation, grey matter, which had been excised from the frontal cortex (1−3 mm anterior with respect to bregma), was rapidly frozen by immersion in –40 °C isopentane prior to being stored at − 80 °C until required.

### Ethical approval

All studies reported had prior approval from the Florey Institute for Neuroscience and Mental Health Animal Ethics Committee.

### RNA extraction and quantification

To measure cortical gene expression in Chrm1^−^^/−^ and w/t, total RNA was isolated from ∼ 50 mg of frozen grey matter using 0.5 ml TRIzol reagent (Life Technologies, Scoresby, VIC, Australia). After homogenisation and phase separation according to the manufacturer’s instructions, an equal volume of 70% ethanol was added to the aqueous phase and RNA was then isolated using RNeasy mini kits (Qiagen, Chadstone Centre, VIC, Australia). All samples were treated with DNase using on-column digestion; the absence of DNA contamination being proven using PCR and primers specific for genomic DNA. RNA quantity and quality were analysed by spectrophotometry (NanoDrop; Thermo Fisher Scientific Australia, Scoresby, VIC, Australia) and by obtaining RNA integrity numbers (RINs) using an Agilent 2100 bioanalyser (Agilent Technologies, Santa Clara, CA, USA). All samples used for the microarray study had RINs of ⩾9.0 to ensure an RNA quality suitable for microarray hybridisation.

Levels of RNA in the mouse cortex were measured at the Australian Genome Research Facility (AGRF; Melbourne, Australia). At the AGRF, ribosomal RNA was eliminated prior to generating cRNA that was end labelled with biotin using the Affymetrix synthesis and labelling kit with random priming. Samples passing the quality checkpoints were prepared for hybridisation using a standard probe cocktail. Each sample was loaded onto an Affymetrix Mouse Exon 1.0 ST Array (Affymetrix, Santa Clara, CA, USA) and hybridised overnight. Following post-hybridisation washes, the chips were scanned and the fluorescent signals converted into a DAT file. After visual confirmation of the scans and quality control analysis, these files were used to generate cell intensity (CEL) and chip (CHP) files for analysis by the authors. Data files are held by the CRC for Mental Health and will be made available to bona fide researchers upon request.

### Data analyses and statistics

All CEL files were imported into JMP Genomics 9.0 (SAS, Cary, NC, USA) at the gene level, collapsing the exon-level data onto known transcripts. To control for array-to-array variation, the data were normalised using the Robust Multichip Average (RMA) algorithm. The data were then log2 transformed and, because they were to be analysed across multiple groups, normalised for between-group comparisons using the ‘Least Square means’ method (see: http://support.sas.com/onlinedoc/913/getDoc/en/statug.hlp/glm_sect34.htm) and then imported using metaprobe set and probe set list files.

Being aware of problems in analysing transcriptomic data^61^, and having compared three recognised criteria for such analyses (Supplementary Material 1), we used the criteria suggested to be appropriate for studies of gene expression in the mouse^62^, which uses a significance of *p* < 0.01 and a fold change in gene expression of ≥ ±0.5 to define a changed level of gene expression between experimental groups. Then, to better understand the potential biological relevance of changes in cortical gene expression in the Chrm1^−^^/−^ mouse, genes with altered levels of expression in the cortex of that mouse were included in analyses using the Panther Gene Ontology Classification System^63^, which identifies pathways and functions that includes genes with a change level of expression at a statistically significant over or under-representation. Finally, we manually reviewed the changes in gene expression in the cortex of Chrm1^−^^/−^ mice to determine if there were clusters of genes that would be expected to impact on specific cellular functions.

In our study design, we were aware it had been standard practice to validate data from transcriptomic technologies using an alternative technology such as in situ hybridisation^64^ or quantitative polymerase chain reaction (qPCR)^65^. We used this approach in a number of our transcriptomic studies and showed that changes in levels of gene expression could be repeatedly validated using qPCR^16,17,46,66,67^. As we had repeatedly shown qPCR does validate results from the Affymetrix gene expression arrays we have more recently accepted the data from these arrays without further validation^18,31,43,68^, which is the design we adopted for this study.

### Data analyses: cross species comparison

To determine if changes in gene expression in the cortex of Chrm1^−^^/−^ had similarities to those in patients with schizophrenia, we compared changes in gene expression in the cortex of that mouse to those in the cortex from patients with schizophrenia^18^. In addition, because of the role of the Chrm1 in cognition^19^, we compared the genes with the changed level of expression in the Chrm1^−^^/−^ mouse to genes associated with changing levels of cortical ability in humans as identified by GWAS^20^.

### Reporting summary

Further information on research design is available in the Nature Research Reporting Summary linked to this article.

## Supplementary information

Supplementary Information

Reporting Summary

## Data Availability

The funding to obtain the core array data (.CEL files) from which the data in this publication have been extracted was from the CRC for Mental Health under a contract that required these data to keep confidential. However, following a review of the core data it was decided that the data on annotated RNA could be made available for publication as these data were not adding to the intellectual property portfolio relating to research translation being undertaken by the CRC for Mental Health. With regards to core data, restrictions currently apply to the availability of the .cel files because some of the data they contain are contributing to the commercialisation IP portfolio of CRC for Mental Health and other stakeholders. Thus, for the next 6 months, access to the core array data may be allowed for bone fide researchers who should initially approach the Corresponding Authors who will seek to establish an agreement with the CRC for Mental Health and other stakeholders for release the data under agreed conditions. It is expected all array data will be freely available after 6 months at which time it will be lodged in a publicly accessible database.
