# Supplementary material for: Changes in cortical gene expression in the muscarinic M1 receptor knockout mouse: potential relevance to schizophrenia, Alzheimer’s disease and cognition
Source: NPJ Schizophr. 2021 Sep 14;7:44. doi: 10.1038/s41537-021-00174-z (PMC8440523; doi:10.1038/s41537-021-00174-z)
Supplement: Supplementary file 1 — Supplementary Information [file 41537_2021_174_MOESM1_ESM.pdf]

Supplementary Table 1: Changes ($p < 0.01$; fold Chrm1$^{-/-}$ / wt ≥± 0.5) in levels of coding and non-coding RNA in the cortex of CHRM1$^{-/-}$ mice.

Gene Name	Gene Symbol	Fold CHRM1$^{-/-}$ / wt	p
mitochondrial inner membrane organizing system 1	Minos1	26.571	<0.0001
mortality factor 4 like 2	Morf4l2	25.392	<0.0001
ubiquitin specific peptidase 17-like D	Usp17ld	13.603	<0.0001
translocase of outer mitochondrial membrane 7 homolog (yeast)	Tomm7	13.368	<0.0001
brain expressed X-linked 2	Bex2	11.646	<0.0001
ring-box 1	Rbx1	10.575	<0.0001
zinc finger, MYND-type containing 8	Zmynd8	9.320	<0.0001
CCR4-NOT transcription complex, subunit 8	Cnot8	8.978	<0.0001
SNF8, ESCRT-II complex subunit, homolog (S. cerevisiae)	Snf8	8.585	<0.0001
heat-responsive protein 12	Hrsp12	8.004	<0.0001
NADH dehydrogenase (ubiquinone) 1 alpha subcomplex, 1	Ndufa1	7.940	<0.0001
zinc finger, HIT type 3	Znhit3	7.921	<0.0001
zinc finger, DHHC domain containing 17	Zdhhc17	7.674	<0.0001
neural precursor cell expressed, developmentally down-regulated gene 8	Nedd8	7.614	<0.0001
proteasome maturation protein	Pomp	7.604	<0.0001
deoxyhypusine hydroxylase/monooxygenase	Dohh	7.365	<0.0001
family with sequence similarity 213, member B	Fam213b	7.249	<0.0001
mitochondrial ribosomal protein S34	Mrps34	7.232	<0.0001
ubiquinol-cytochrome c reductase complex assembly factor 3	Uqcc3	7.031	<0.0001
immediate early response 3 interacting protein 1	Ier3ip1	6.843	<0.0001
eukaryotic translation initiation factor 1	Eif1	6.815	<0.0001
platelet-activating factor receptor	Ptafr	6.791	<0.0001
CLP1, cleavage and polyadenylation factor I subunit	Clp1	6.737	<0.0001
cytochrome c oxidase assembly protein 14	Cox14	6.596	<0.0001
biliverdin reductase A	Blvra	6.538	<0.0001
mitochondrial ribosomal protein S12	Mrps12	6.113	<0.0001
StAR-related lipid transfer (START) domain containing 4	Stard4	6.073	<0.0001
motile sperm domain containing 1	Mospd1	5.970	<0.0001
signal peptidase complex subunit 1 homolog (S. cerevisiae)	Spcs1	5.908	<0.0001
split hand/foot malformation (ectrodactyly) type 1	Shfm1	5.784	<0.0001

Rab geranylgeranyl transferase, b subunit	Rabggtb	5.605	<0.0001
sprouty homolog 2 (Drosophila)	Spry2	5.479	<0.0001
polycomb group ring finger 1	Pcgf1	5.448	<0.0001
mago homolog, exon junction complex core component	Magoh	5.389	<0.0001
yrdC domain containing (E.coli)	Yrdc	5.381	<0.0001
WW domain binding protein 5	Wbp5	5.367	<0.0001
general transcription factor IIB	Gtf2b	5.348	<0.0001
SWI5 recombination repair homolog (yeast)	Swi5	5.296	<0.0001
cathepsin S	Ctss	5.294	<0.0001
FK506 binding protein 2	Fkbp2	5.286	<0.0001
programmed cell death 10	Pdcd10	5.281	<0.0001
transmembrane protein 14C	Tmem14c	5.187	<0.0001
integral membrane protein 2A	Itm2a	5.158	<0.0001
mitochondrial ribosomal protein S18C	Mrps18c	5.081	<0.0001
cleavage stimulation factor, 3 pre-RNA subunit 2, tau	Cstf2t	4.767	<0.0001
autophagy related 101	Atg101	4.759	<0.0001
translocase of inner mitochondrial membrane 10	Timm10	4.709	<0.0001
CD9 antigen	Cd9	4.702	<0.0001
cilia and flagella associated protein 36	Cfap36	4.657	<0.0001
propionyl Coenzyme A carboxylase, beta polypeptide	Pccb	4.632	<0.0001
NADH dehydrogenase (ubiquinone) 1 beta subcomplex, 7	Ndufb7	4.518	<0.0001
TAF11 RNA polymerase II, TATA box binding protein (TBP)-associated factor	Taf11	4.478	<0.0001
eukaryotic translation initiation factor 3, subunit E	Eif3e	4.473	<0.0001
family with sequence similarity 173, member A	Fam173a	4.456	<0.0001
TSR3 20S rRNA accumulation	Tsr3	4.427	<0.0001
NADH dehydrogenase (ubiquinone) 1 beta subcomplex 8	Ndufb8	4.427	<0.0001
histocompatibility 2, D region locus 1	H2-D1	4.388	<0.0001
DnaJ (Hsp40) homolog, subfamily C, member 12	Dnajc12	4.370	<0.0001
coronin, actin binding protein 1A	Coro1a	4.328	<0.0001
lymphocyte antigen 6 complex, locus H	Ly6h	4.317	<0.0001
phosducin-like	Pdcl	4.241	<0.0001
transmembrane protein 11	Tmem11	4.231	<0.0001
proteasome (prosome, macropain) subunit, beta type 6	Psmb6	4.16	<0.0001
glyoxalase domain containing 4	Glod4	4.142	<0.0001

coiled-coil domain containing 59	Ccdc59	4.125	<0.0001
complement component 1, q subcomponent, alpha polypeptide	C1qa	4.082	<0.0001
peroxiredoxin 4	Prdx4	4.074	<0.0001
RAB18, member RAS oncogene family	Rab18	4.065	<0.0001
SUB1 homolog (S. cerevisiae)	Sub1	4.063	<0.0001
mitochondrial ribosomal protein S17	Mrps17	4.053	<0.0001
F-box protein 30	Fbxo30	4.019	<0.0001
ubiquinol-cytochrome c reductase, complex III subunit X	Uqcr10	3.978	<0.0001
branched chain ketoacid dehydrogenase E1, alpha polypeptide	Bckdha	3.977	<0.0001
MPN domain containing	Mpnd	3.971	<0.0001
reticulocalbin 2	Rcn2	3.955	<0.0001
makorin, ring finger protein, 1	Mkrn1	3.930	<0.0001
branched chain ketoacid dehydrogenase kinase	Bckdk	3.920	<0.0001
GPN-loop GTPase 3	Gpn3	3.838	<0.0001
2,3-bisphosphoglycerate mutase	Bpgm	3.833	<0.0001
cytochrome b-561	Cyb561	3.829	<0.0001
LSM1 homolog, U6 small nuclear RNA associated (S. cerevisiae)	Lsm1	3.808	<0.0001
small integral membrane protein 12	Smim12	3.799	0.0001
immunoglobulin (CD79A) binding protein 1	Igbp1	3.798	<0.0001
ring finger protein 7	Rnf7	3.778	<0.0001
glyoxalase 1	Glo1	3.767	<0.0001
differentially expressed in B16F10 1	Deb1	3.760	<0.0001
cold inducible RNA binding protein	Cirbp	3.758	<0.0001
ribosomal protein S4-like	Rps4l	3.745	<0.0001
TSR1 20S rRNA accumulation	Tsr1	3.737	<0.0001
cytochrome c oxidase assembly protein 17	Cox17	3.733	<0.0001
translocase of outer mitochondrial membrane 40 homolog-like (yeast)	Tomm40l	3.730	<0.0001
nudix (nucleoside diphosphate linked moiety X)-type motif 11	Nudt11	3.726	<0.0001
serine/threonine kinase 38 like	Stk38l	3.719	<0.0001
microsomal glutathione S-transferase 3	Mgst3	3.705	<0.0001
mitochondrial ribosomal protein S26	Mrps26	3.701	<0.0001
vesicle transport through interaction with t-SNAREs 1B	Vti1b	3.695	<0.0001
mitochondrial ribosomal protein L2	Mrpl2	3.695	<0.0001
TAF13 RNA polymerase II, TATA box binding protein (TBP)-associated factor	Taf13	3.615	<0.0001

apurinic/apyrimidinic endonuclease 1	Apex1	3.612	<0.0001
ribosomal protein S3	Rps3	3.603	<0.0001
interferon-related developmental regulator 1	Ifrd1	3.571	<0.0001
cellular nucleic acid binding protein	Cnbp	3.566	<0.0001
transducin (beta)-like 3	Tbl3	3.560	<0.0001
adipocyte plasma membrane associated protein	Apmap	3.540	<0.0001
DNA-damage-inducible transcript 4	Ddit4	3.524	<0.0001
proteasome (prosome, macropain) 26S subunit, non-ATPase, 4	Psmd4	3.511	<0.0001
suppressor of cytokine signaling 6	Socs6	3.506	<0.0001
RAB28, member RAS oncogene family	Rab28	3.504	<0.0001
NADH dehydrogenase (ubiquinone) 1 alpha subcomplex, assembly factor 2	Ndufaf2	3.488	<0.0001
mitochondrial ribosomal protein L24	Mrpl24	3.473	<0.0001
antizyme inhibitor 1	Azin1	3.462	<0.0001
C1GALT1-specific chaperone 1	C1galt1c1	3.462	<0.0001
MAF1 homolog (S. cerevisiae)	Maf1	3.459	<0.0001
nuclear import 7 homolog (S. cerevisiae)	Nip7	3.450	<0.0001
proteasome (prosome, macropain) 26S subunit, ATPase 2	Psmc2	3.447	<0.0001
polymerase (RNA) I polypeptide D	Polr1d	3.442	<0.0001
solute carrier family 40 (iron-regulated transporter), member 1	Slc40a1	3.435	<0.0001
nuclear receptor coactivator 4	Ncoa4	3.431	<0.0001
ribosomal protein S14	Rps14	3.431	<0.0001
PITH (C-terminal proteasome-interacting domain of thioredoxin-like) domain containing 1	Pithd1	3.428	<0.0001
pantothenate kinase 3	Pank3	3.413	<0.0001
zinc finger protein 637	Zfp637	3.393	<0.0001
phosphomannomutase 1	Pmm1	3.359	<0.0001
ribosomal protein L31	Rpl31	3.352	<0.0001
electron transferring flavoprotein, dehydrogenase	Etfdh	3.328	<0.0001
proteasome (prosome, macropain) subunit, alpha type 7	Psma7	3.324	<0.0001
collagen, type IV, alpha 3 (Goodpasture antigen) binding protein	Col4a3bp	3.320	<0.0001
proteasome (prosome, macropain) assembly chaperone 1	Psmg1	3.300	<0.0001
SEC11 homolog C, signal peptidase complex subunit	Sec11c	3.270	<0.0001
mitochondrial ribosomal protein L41	Mrpl41	3.235	<0.0001
zinc finger, CCHC domain containing 12	Zcchc12	3.233	<0.0001
6-pyruvoyl-tetrahydropterin synthase	Pts	3.226	<0.0001

echinoderm microtubule associated protein like 2	Eml2	3.225	<0.0001
paraneoplastic antigen MA2	Pnma2	3.222	<0.0001
signal recognition particle 14	Srp14	3.220	<0.0001
zinc finger protein 330	Zfp330	3.210	<0.0001
ribosomal protein S9	Rps9	3.201	<0.0001
chibby homolog 1 (Drosophila)	Cby1	3.201	<0.0001
demethyl-Q 7	Coq7	3.200	<0.0001
succinate-CoA ligase, GDP-forming, alpha subunit	Suclg1	3.200	<0.0001
WD repeat domain 73	Wdr73	3.174	<0.0001
methylsterol monoxygenase 1	Msmo1	3.170	<0.0001
megalencephalic leukoencephalopathy with subcortical cysts 1 homolog (human)	Mlc1	3.168	<0.0001
thioredoxin domain containing 5	Txndc5	3.146	<0.0001
RNA polymerase II associated protein 3	Rpap3	3.138	<0.0001
adipocyte-related X-chromosome expressed sequence 2	Arxes2	3.136	<0.0001
magnesium-dependent phosphatase 1	Mdp1	3.135	<0.0001
acyl-CoA thioesterase 9	Acot9	3.132	<0.0001
transferrin receptor	Tfrc	3.131	<0.0001
phosphomevalonate kinase	Pmvk	3.130	<0.0001
synovial sarcoma, X breakpoint 2 interacting protein	Ssx2ip	3.127	<0.0001
autophagy related 4B, cysteine peptidase	Atg4b	3.121	<0.0001
integrin beta 1 (fibronectin receptor beta)	Itgb1	3.103	<0.0001
OTU domain containing 6B	Otud6b	3.100	<0.0001
energy homeostasis associated	Enho	3.098	<0.0001
peptidylprolyl isomerase D (cyclophilin D)	Ppid	3.096	<0.0001
kelch repeat and BTB (POZ) domain containing 2	Kbtbd2	3.088	<0.0001
receptor (calcitonin) activity modifying protein 3	Ramp3	3.080	<0.0001
coiled-coil domain containing 62	Ccdc62	3.076	<0.0001
serine/threonine kinase 32C	Stk32c	3.074	<0.0001
synaptotagmin IV	Syt4	3.068	<0.0001
related RAS viral (r-ras) oncogene homolog 2	Rras2	3.061	<0.0001
ATP synthase, H+ transporting, mitochondrial F1 complex, O subunit	Atp5o	3.055	<0.0001
f-box protein 9	Fbxo9	3.053	<0.0001
adipogenesis associated Mth938 domain containing	Aamdc	3.051	<0.0001
zinc finger protein 251	Zfp251	3.044	<0.0001

myeloid derived growth factor	Mydgf	3.044	<0.0001
DDB1 and CUL4 associated factor 12-like 1	Dcaf12l1	3.042	<0.0001
store-operated calcium entry-associated regulatory factor	Saraf	3.037	<0.0001
chitinase domain containing 1	Chid1	3.032	<0.0001
splicing factor 3b, subunit 5	Sf3b5	3.032	<0.0001
N-terminal EF-hand calcium binding protein 3	Necab3	3.025	<0.0001
ADP-ribosylation factor-like 4D	Arl4d	3.018	<0.0001
guanine deaminase	Gda	3.009	<0.0001
protein phosphatase 4, regulatory subunit 2	Ppp4r2	3.002	<0.0001
activin receptor IIA	Acvr2a	2.996	<0.0001
SUMO/sentrin specific peptidase 8	Senp8	2.993	<0.0001
gene rich cluster, C10 gene	Grcc10	2.983	<0.0001
protein disulfide isomerase associated 6	Pdia6	2.979	<0.0001
Ras-like without CAAX 1	Rit1	2.964	<0.0001
RAB3 GTPase activating protein subunit 1	Rab3gap1	2.963	<0.0001
vacuolar protein sorting 4b (yeast)	Vps4b	2.962	<0.0001
N-6 adenine-specific DNA methyltransferase 2 (putative)	N6amt2	2.954	<0.0001
signal peptidase complex subunit 3 homolog (S. cerevisiae)	Spcs3	2.951	<0.0001
RAP2B, member of RAS oncogene family	Rap2b	2.950	<0.0001
prostaglandin E synthase 2	Ptges2	2.947	<0.0001
mRNA turnover 4, homolog (S. cerevisiae)	Mrto4	2.942	<0.0001
GTPase activating protein (SH3 domain) binding protein 1	G3bp1	2.935	<0.0001
magnesium transporter 1	Magt1	2.934	<0.0001
STIP1 homology and U-Box containing protein 1	Stub1	2.929	<0.0001
family with sequence similarity 188, member A	Fam188a	2.926	<0.0001
selenoprotein T	Selt	2.919	<0.0001
beclin 1, autophagy related	Becn1	2.919	<0.0001
hydroxyacyl glutathione hydrolase	Hagh	2.894	<0.0001
protein tyrosine phosphatase, mitochondrial 1	Ptpmt1	2.888	<0.0001
ORM1-like 3 (S. cerevisiae)	Ormdl3	2.882	<0.0001
acetoacetyl-CoA synthetase	Aacs	2.875	<0.0001
solute carrier family 33 (acetyl-CoA transporter), member 1	Slc33a1	2.871	<0.0001
neuron derived neurotrophic factor	Nenf	2.870	<0.0001
ceroid-lipofuscinosis, neuronal 8	Cln8	2.867	<0.0001

chromodomain helicase DNA binding protein 3, opposite strand	Chd3os	2.856	<0.0001
histidyl-tRNA synthetase	Hars	2.845	<0.0001
transmembrane protein 100	Tmem100	2.840	<0.0001
exosome component 9	Exosc9	2.835	<0.0001
glutathione S-transferase, alpha 4	Gsta4	2.808	<0.0001
acyl-Coenzyme A binding domain containing 5	Acbd5	2.805	<0.0001
CD2 antigen (cytoplasmic tail) binding protein 2	Cd2bp2	2.799	<0.0001
ubiquinol-cytochrome c reductase, Rieske iron-sulfur polypeptide 1	Uqcrfs1	2.796	<0.0001
LSM6 homolog, U6 small nuclear RNA associated (S. cerevisiae)	Lsm6	2.789	<0.0001
G elongation factor, mitochondrial 1	Gfm1	2.776	<0.0001
WD repeat domain 37	Wdr37	2.775	<0.0001
transmembrane protein 222	Tmem222	2.760	<0.0001
BRCA2 and CDKN1A interacting protein	Bccip	2.759	<0.0001
vacuolar protein sorting 16 (yeast)	Vps16	2.750	<0.0001
ubiquitin-conjugating enzyme E2W (putative)	Ube2w	2.739	<0.0001
RAS-like, family 11, member B	Rasl11b	2.737	<0.0001
translational activator of mitochondrially encoded cytochrome c oxidase I	Taco1	2.734	<0.0001
transcription elongation factor A (SII)-like 8	Tceal8	2.733	<0.0001
gamma-aminobutyric acid (GABA) A receptor-associated protein-like 2	Gabarapl2	2.731	<0.0001
NudC domain containing 2	Nudcd2	2.727	<0.0001
malectin	Mlec	2.721	<0.0001
G patch domain and KOW motifs	Gpkow	2.718	<0.0001
protein arginine N-methyltransferase 2	Prmt2	2.718	<0.0001
F-box protein 45	Fbxo45	2.715	<0.0001
glutathione S-transferase, pi 1	Gstp1	2.708	<0.0001
ubiquitin-conjugating enzyme E2M	Ube2m	2.702	<0.0001
antizyme inhibitor 2	Azin2	2.702	<0.0001
ankyrin repeat and SOCS box-containing 1	Asb1	2.690	<0.0001
ribosomal protein L22	Rpl22	2.689	<0.0001
syntaxin 7	Stx7	2.689	<0.0001
Rab40B, member RAS oncogene family	Rab40b	2.688	<0.0001
CDK5 regulatory subunit associated protein 3	Cdk5rap3	2.675	<0.0001
serine/threonine kinase 17b (apoptosis-inducing)	Stk17b	2.667	<0.0001
insulin induced gene 1	Insig1	2.666	<0.0001

pygopus 2	Pygo2	2.665	<0.0001
GIPC PDZ domain containing family, member 1	Gipc1	2.661	<0.0001
cathepsin F	Ctsf	2.660	<0.0001
unc-119 homolog (C. elegans)	Unc119	2.660	<0.0001
phosphoglucomutase 2	Pgm2	2.656	<0.0001
pleckstrin and Sec7 domain containing 3	Psd3	2.653	<0.0001
molybdenum cofactor synthesis 2	Mocs2	2.649	<0.0001
ATP-binding cassette, sub-family B (MDR/TAP), member 1A	Abcb1a	2.647	<0.0001
SEC11 homolog A, signal peptidase complex subunit	Sec11a	2.643	<0.0001
pyruvate dehydrogenase complex, component X	Pdhx	2.641	<0.0001
ribosomal protein L8	Rpl8	2.629	<0.0001
FK506 binding protein 4	Fkbp4	2.628	<0.0001
protein phosphatase 2, regulatory subunit B, delta	Ppp2r2d	2.627	<0.0001
NADH dehydrogenase (ubiquinone) 1 alpha subcomplex, 5	Ndufa5	2.623	<0.0001
spinster homolog 1	Spns1	2.621	<0.0001
dynein cytoplasmic 1 intermediate chain 1	Dync1i1	2.619	<0.0001
secretogranin V	Scg5	2.613	<0.0001
cell adhesion molecule 4	Cadm4	2.609	<0.0001
zinc finger protein 24	Zfp24	2.592	<0.0001
actin related protein 2/3 complex, subunit 3	Arpc3	2.590	<0.0001
pyruvate dehydrogenase E1 alpha 1	Pdha1	2.587	<0.0001
serine protease inhibitor, Kunitz type 2	Spint2	2.585	<0.0001
Na+/K+ transporting ATPase interacting 4	Nkain4	2.584	<0.0001
ER membrane protein complex subunit 9	Emc9	2.583	<0.0001
NOL1/NOP2/Sun domain family member 2	Nsun2	2.582	<0.0001
zinc finger protein 239	Zfp239	2.579	<0.0001
ubiquitin-conjugating enzyme E2E 1	Ube2e1	2.577	<0.0001
yippee-like 1 (Drosophila)	Ypel1	2.573	<0.0001
melanoma antigen, family H, 1	Mageh1	2.570	<0.0001
p53 and DNA damage regulated 1	Pdrg1	2.570	<0.0001
prolyl 4-hydroxylase, transmembrane (endoplasmic reticulum)	P4htm	2.569	<0.0001
Crn, crooked neck-like 1 (Drosophila)	Crnkl1	2.554	<0.0001
SAC1 (suppressor of actin mutations 1, homolog)-like (S. cerevisiae)	Sacm1l	2.554	<0.0001
F-box and WD-40 domain protein 7	Fbxw7	2.545	<0.0001

coiled-coil domain containing 25	Ccdc25	2.545	<0.0001
cyclin-dependent kinase 5, regulatory subunit 1 (p35)	Cdk5r1	2.544	<0.0001
serine palmitoyltransferase, small subunit A	Sptssa	2.544	<0.0001
solute carrier family 39 (zinc transporter), member 12	Slc39a12	2.533	<0.0001
coiled-coil-helix-coiled-coil-helix domain containing 4	Chchd4	2.531	<0.0001
mitochondrial ribosomal protein L11	Mrpl11	2.531	<0.0001
DNA segment, Chr 10, Johns Hopkins University 81 expressed	D10Jhu81e	2.529	<0.0001
RAD51 homolog D	Rad51d	2.524	<0.0001
serine (or cysteine) peptidase inhibitor, clade I, member 1	Serpini1	2.516	<0.0001
proteasome (prosome, macropain) subunit, beta type 1	Psmb1	2.515	<0.0001
electron transferring flavoprotein, alpha polypeptide	Etfa	2.512	<0.0001
ubiquitin specific peptidase 5 (isopeptidase T)	Usp5	2.507	<0.0001
proteasome (prosome, macropain) activator subunit 1 (PA28 alpha)	Psme1	2.506	<0.0001
S100 calcium binding protein A6 (calcyclin)	S100a6	2.503	<0.0001
transmembrane emp24-like trafficking protein 10 (yeast)	Tmed10	2.497	<0.0001
solute carrier family 35 (UDP-N-acetylglucosamine (UDP-GlcNAc) transporter), member 3	Slc35a3	2.493	<0.0001
3-phosphoadenosine 5-phosphosulfate synthase 1	Papss1	2.493	<0.0001
ATP-binding cassette, sub-family B (MDR/TAP), member 8	Abcb8	2.492	<0.0001
vacuolar protein sorting 51 homolog (S. cerevisiae)	Vps51	2.491	<0.0001
intraflagellar transport 52	Ift52	2.486	<0.0001
proteasome (prosome, macropain) subunit, alpha type 3	Psma3	2.477	<0.0001
ArfGAP with coiled-coil, ankyrin repeat and PH domains 3	Acap3	2.475	<0.0001
tetraspanin 15	Tspan15	2.474	<0.0001
pleckstrin homology domain containing, family J member 1	Plekhj1	2.474	<0.0001
FK506 binding protein 9	Fkbp9	2.471	<0.0001
zinc finger, AN1-type domain 6	Zfand6	2.470	<0.0001
eukaryotic translation initiation factor 2, subunit 3, structural gene X-linked	Eif2s3x	2.469	<0.0001
aryl-hydrocarbon receptor-interacting protein	Aip	2.467	<0.0001
solute carrier family 5 (sodium-dependent vitamin transporter), member 6	Slc5a6	2.465	<0.0001
RNA binding motif protein 12 B2	Rbm12b2	2.459	<0.0001
tumor necrosis factor alpha induced protein 6	Tnfaip6	2.458	<0.0001
ankyrin repeat domain 46	Ankrd46	2.456	<0.0001
neuron specific gene family member 1	Nsg1	2.452	<0.0001
ubiquitin carboxy-terminal hydrolase L1	Uchl1	2.445	<0.0001

adaptor-related protein complex 2, sigma 1 subunit	Ap2s1	2.444	<0.0001
polymerase (RNA) II (DNA directed) polypeptide B	Polr2b	2.444	<0.0001
kelch-like 9	Klhl9	2.440	<0.0001
transmembrane protein 199	Tmem199	2.438	<0.0001
ubiquinol-cytochrome c reductase, complex III subunit VII	Uqcrq	2.437	<0.0001
progestin and adipoQ receptor family member VII	Paqr7	2.433	<0.0001
tubulin folding cofactor B	Tbcb	2.432	<0.0001
translocase of inner mitochondrial membrane 44	Timm44	2.425	<0.0001
zinc finger protein 445	Zfp445	2.423	<0.0001
translocase of inner mitochondrial membrane 17a	Timm17a	2.421	<0.0001
thioredoxin domain containing 15	Txndc15	2.418	<0.0001
FGGY carbohydrate kinase domain containing	Fggy	2.416	<0.0001
uridine monophosphate synthetase	Umps	2.414	<0.0001
ADP-ribosylarginine hydrolase	Adprh	2.414	<0.0001
protein phosphatase 1K (PP2C domain containing)	Ppm1k	2.401	<0.0001
DAZ associated protein 2	Dazap2	2.394	<0.0001
death-associated protein kinase 3	Dapk3	2.394	<0.0001
DEAD/H (Asp-Glu-Ala-Asp/His) box polypeptide 3, X-linked	Ddx3x	2.393	<0.0001
reprimo, TP53 dependent G2 arrest mediator candidate	Rprm	2.391	<0.0001
SUZ RNA binding domain containing 1	Szrd1	2.391	<0.0001
tetratricopeptide repeat domain 27	Ttc27	2.388	<0.0001
drebrin-like	Dbnl	2.385	<0.0001
bolA-like 2 (E. coli)	Bola2	2.384	<0.0001
ubiquinol-cytochrome c reductase core protein 1	Uqcrc1	2.383	<0.0001
sulfotransferase family 4A, member 1	Sult4a1	2.381	<0.0001
NOP58 ribonucleoprotein	Nop58	2.380	<0.0001
DnaJ (Hsp40) homolog, subfamily C, member 10	Dnajc10	2.378	<0.0001
cysteine dioxygenase 1, cytosolic	Cdo1	2.375	<0.0001
solute carrier family 25 (mitochondrial carrier, phosphate carrier), member 25	Slc25a25	2.372	<0.0001
CCAAT/enhancer binding protein (C/EBP), gamma	Cebpg	2.371	<0.0001
DDB1 and CUL4 associated factor 11	Dcaf11	2.366	<0.0001
selenophosphate synthetase 2	Sephs2	2.366	<0.0001
DNA segment, Chr 6, Wayne State University 163, expressed	D6Wsu163e	2.366	<0.0001
attractin like 1	Atrnl1	2.365	<0.0001

zinc finger protein 146	Zfp146	2.363	<0.0001
mitochondrial ribosomal protein L55	Mrpl55	2.363	<0.0001
lon peptidase 1, mitochondrial	Lonp1	2.357	<0.0001
heparan-alpha-glucosaminide N-acetyltransferase	Hgsnat	2.356	<0.0001
solute carrier family 16 (monocarboxylic acid transporters), member 1	Slc16a1	2.355	<0.0001
DEAD (Asp-Glu-Ala-Asp) box polypeptide 3, Y-linked	Ddx3y	2.351	<0.0001
adaptor-related protein complex AP-4, beta 1	Ap4b1	2.349	<0.0001
acyl-Coenzyme A dehydrogenase family, member 8	Acad8	2.344	<0.0001
casein kinase 2, alpha 1 polypeptide	Csnk2a1	2.344	<0.0001
sperm associated antigen 7	Spag7	2.343	<0.0001
M phase phosphoprotein 6	Mphosph6	2.342	0.0001
mitochondrial ribosomal protein L14	Mrpl14	2.340	<0.0001
family with sequence similarity 19, member A2	Fam19a2	2.339	<0.0001
insulin-like growth factor binding protein 2	Igfbp2	2.337	<0.0001
biphenyl hydrolase-like (serine hydrolase, breast epithelial mucin-associated antigen)	Bphl	2.337	<0.0001
annexin A6	Anxa6	2.328	<0.0001
SV2 related protein	Svop	2.328	<0.0001
FtsJ homolog 2 (E. coli)	Ftsj2	2.326	<0.0001
tetratricopeptide repeat domain 30B	Ttc30b	2.326	<0.0001
transmembrane protein 218	Tmem218	2.317	<0.0001
mitochondrial ribosomal protein L9	Mrpl9	2.316	<0.0001
potassium large conductance calcium-activated channel, subfamily M, beta member 4	Kcnmb4	2.316	<0.0001
actin related protein 2/3 complex, subunit 5-like	Arpc5l	2.314	<0.0001
growth associated protein 43	Gap43	2.311	<0.0001
phosphoribosyl pyrophosphate amidotransferase	Ppat	2.310	<0.0001
melanoma antigen, family E, 1	Magee1	2.307	<0.0001
aminoacyl tRNA synthetase complex-interacting multifunctional protein 1	Aimp1	2.307	<0.0001
adrenergic receptor, beta 1	Adrb1	2.306	<0.0001
serine (or cysteine) peptidase inhibitor, clade E, member 2	Serpine2	2.304	<0.0001
apolipoprotein L domain containing 1	Apold1	2.303	<0.0001
protein kinase C and casein kinase substrate in neurons 3	Pacsin3	2.303	<0.0001
armadillo repeat containing 10	Armc10	2.302	<0.0001
phospholipase A2, group XVI	Pla2g16	2.301	<0.0001
aldehyde dehydrogenase family 6, subfamily A1	Aldh6a1	2.296	<0.0001

protein arginine N-methyltransferase 1	Prmt1	2.294	<0.0001
ring finger protein 8	Rnf8	2.294	<0.0001
flotillin 1	Flot1	2.289	<0.0001
transmembrane protein 14A	Tmem14a	2.286	<0.0001
coiled-coil domain containing 126	Ccdc126	2.286	<0.0001
phosphofructokinase, liver, B-type	Pfkl	2.285	<0.0001
nucleosome assembly protein 1-like 3	Nap1l3	2.280	<0.0001
family with sequence similarity 105, member A	Fam105a	2.275	<0.0001
adenosine kinase	Adk	2.271	<0.0001
5,3-nucleotidase, cytosolic	Nt5c	2.269	<0.0001
transmembrane protein 242	Tmem242	2.268	<0.0001
ligase IV, DNA, ATP-dependent	Lig4	2.266	<0.0001
regulator of G-protein signaling 4	Rgs4	2.266	<0.0001
RNA (guanine-7-) methyltransferase	Rnmt	2.260	<0.0001
four jointed box 1 (Drosophila)	Fjx1	2.259	<0.0001
peroxiredoxin 3	Prdx3	2.258	<0.0001
matrilin 2	Matn2	2.257	<0.0001
adaptor protein complex AP-1, beta 1 subunit	Ap1b1	2.256	<0.0001
kelch domain containing 2	Klhdc2	2.254	<0.0001
down-regulator of transcription 1	Dr1	2.254	<0.0001
vacuolar protein sorting 11 (yeast)	Vps11	2.252	<0.0001
methylmalonic aciduria (cobalamin deficiency) cblD type, with homocystinuria	Mmadhc	2.250	<0.0001
potassium inwardly-rectifying channel, subfamily J, member 9	Kcnj9	2.246	<0.0001
jumonji domain containing 4	Jmjd4	2.242	<0.0001
chaperonin containing Tcp1, subunit 8 (theta)	Cct8	2.241	<0.0001
mitchondrial ribosomal protein S7	Mrps7	2.240	<0.0001
YKT6 homolog (S. Cerevisiae)	Ykt6	2.236	<0.0001
ER membrane protein complex subunit 8	Emc8	2.236	<0.0001
poly(A) binding protein interacting protein 2B	Paip2b	2.229	<0.0001
amyloid beta (A4) precursor protein-binding, family B, member 1	Apbb1	2.229	<0.0001
leucyl-tRNA synthetase	Lars	2.229	<0.0001
arginyltransferase 1	Ate1	2.228	<0.0001
platelet-activating factor acetylhydrolase, isoform 1b, subunit 2	Pafah1b2	2.223	<0.0001
GPALPP motifs containing 1	Gpalpp1	2.222	<0.0001

gap junction protein, alpha 1	Gja1	2.220	<0.0001
microtubule-associated protein 1 light chain 3 alpha	Map1lc3a	2.217	<0.0001
secretion regulating guanine nucleotide exchange factor	Sergef	2.209	<0.0001
DnaJ (Hsp40) homolog, subfamily A, member 1	Dnaja1	2.207	0.0005
Sjogrens syndrome nuclear autoantigen 1	Ssna1	2.205	<0.0001
translin	Tsn	2.203	<0.0001
high mobility group box 1	Hmgb1	2.203	<0.0001
argininosuccinate lyase	Asl	2.202	<0.0001
succinate dehydrogenase complex, subunit D, integral membrane protein	Sdhd	2.202	<0.0001
MOB kinase activator 2	Mob2	2.202	<0.0001
phosphoribosyl pyrophosphate synthetase 1	Prps1	2.198	<0.0001
NADH dehydrogenase (ubiquinone) Fe-S protein 2	Ndufs2	2.193	<0.0001
reticulon 4	Rtn4	2.192	<0.0001
DnaJ (Hsp40) homolog, subfamily C, member 7	Dnajc7	2.192	<0.0001
DEAD (Asp-Glu-Ala-Asp) box polypeptide 42	Ddx42	2.190	<0.0001
golgi to ER traffic protein 4 homolog (S. cerevisiae)	Get4	2.190	<0.0001
abhydrolase domain containing 8	Abhd8	2.189	<0.0001
transmembrane protein 87B	Tmem87b	2.188	<0.0001
pyridoxine 5-phosphate oxidase	Pnpo	2.188	<0.0001
diphthamine biosynethesis 7	Dph7	2.187	<0.0001
family with sequence similarity 175, member B	Fam175b	2.187	<0.0001
armadillo repeat containing, X-linked 3	Armcx3	2.186	<0.0001
transmembrane protein 80	Tmem80	2.185	<0.0001
staphylococcal nuclease and tudor domain containing 1	Snd1	2.183	<0.0001
transmembrane protein 223	Tmem223	2.178	<0.0001
S100 protein, beta polypeptide, neural	S100b	2.177	<0.0001
pentatricopeptide repeat domain 2	Ptcd2	2.177	<0.0001
SUMO/sentrin specific peptidase 2	Senp2	2.176	<0.0001
acylphosphatase 1, erythrocyte (common) type	Acyp1	2.175	<0.0001
general transcription factor II E, polypeptide 1 (alpha subunit)	Gtf2e1	2.174	<0.0001
RAP2C, member of RAS oncogene family	Rap2c	2.171	<0.0001
chaperonin containing Tcp1, subunit 3 (gamma)	Cct3	2.169	<0.0001
polymerase (RNA) II (DNA directed) polypeptide G	Polr2g	2.168	<0.0001
cytochrome c oxidase subunit Va	Cox5a	2.165	<0.0001

monoglyceride lipase	Mgll	2.163	<0.0001
growth arrest specific 5	Gas5	2.162	<0.0001
kelch-like 28	Klhl28	2.162	<0.0001
stathmin-like 4	Stmn4	2.161	<0.0001
solute carrier family 25 (mitochondrial carrier, brain), member 14	Slc25a14	2.158	<0.0001
tubulin, beta 2A class IIA	Tubb2a	2.157	<0.0001
late endosomal/lysosomal adaptor, MAPK and MTOR activator 2	Lamtor2	2.157	<0.0001
transmembrane protein 50A	Tmem50a	2.156	<0.0001
DCN1, defective in cullin neddylation 1, domain containing 1 (S. cerevisiae)	Dcun1d1	2.156	<0.0001
tetratricopeptide repeat domain 33	Ttc33	2.155	<0.0001
peroxiredoxin 5	Prdx5	2.153	<0.0001
sepiapterin reductase	Spr	2.153	<0.0001
tumor suppressor candidate 3	Tusc3	2.149	<0.0001
phosphorylated adaptor for RNA export	Phax	2.148	<0.0001
proteasome (prosome, macropain) 26S subunit, ATPase 3	Psmc3	2.147	<0.0001
mitogen-activated protein kinase kinase 1	Map2k1	2.146	<0.0001
dysbindin (dystrobrevin binding protein 1) domain containing 2	Dbndd2	2.144	<0.0001
eukaryotic translation initiation factor 1A domain containing	Eif1ad	2.141	<0.0001
coiled-coil domain containing 149	Ccdc149	2.140	<0.0001
neuron specific gene family member 2	Nsg2	2.139	<0.0001
poly(A)-specific ribonuclease (deadenylation nuclease)	Parn	2.134	<0.0001
potassium voltage-gated channel, subfamily F, member 1	Kcnf1	2.132	<0.0001
smu-1 suppressor of mec-8 and unc-52 homolog (C. elegans)	Smu1	2.132	<0.0001
zinc finger, AN1-type domain 5	Zfand5	2.132	<0.0001
serum response factor binding protein 1	Srfbp1	2.131	<0.0001
regulator of G-protein signaling 14	Rgs14	2.130	<0.0001
activin A receptor, type 1B	Acvr1b	2.130	<0.0001
MAM domain containing 4	Mamdc4	2.130	<0.0001
bleomycin hydrolase	Blmh	2.130	<0.0001
IAP promoted placental gene	Ipp	2.128	<0.0001
NADH dehydrogenase (ubiquinone) 1 alpha subcomplex, 6 (B14)	Ndufa6	2.127	<0.0001
proteasome (prosome, macropain) 26S subunit, non-ATPase, 6	Psmd6	2.126	<0.0001
platelet-activating factor acetylhydrolase, isoform 1b, subunit 1	Pafah1b1	2.124	<0.0001
NADH dehydrogenase (ubiquinone) 1 alpha subcomplex, 8	Ndufa8	2.122	<0.0001

junctophilin 3	Jph3	2.121	<0.0001
solute carrier family 13 (sodium-dependent citrate transporter), member 5	Slc13a5	2.120	<0.0001
ARP2 actin-related protein 2	Actr2	2.120	<0.0001
RNA binding motif protein, X-linked like-1	Rbmxl1	2.119	<0.0001
solute carrier family 25, member 33	Slc25a33	2.119	<0.0001
cytosolic iron-sulfur protein assembly 1	Ciao1	2.117	<0.0001
ubiquitin fusion degradation 1 like	Ufd1l	2.117	<0.0001
5-hydroxytryptamine (serotonin) receptor 2C	Htr2c	2.116	<0.0001
IQ motif and Sec7 domain 1	Iqsec1	2.115	<0.0001
calcium regulated heat stable protein 1	Carhsp1	2.111	<0.0001
ATP5S-like	Atp5sl	2.111	<0.0001
transmembrane and ubiquitin-like domain containing 1	Tmub1	2.109	<0.0001
protease, serine 12 neurotrypsin (motopsin)	Prss12	2.109	<0.0001
coiled-coil domain containing 186	Ccdc186	2.105	<0.0001
adipocyte-related X-chromosome expressed sequence 1	Arxes1	2.102	<0.0001
polo-like kinase 2	Plk2	2.099	<0.0001
RAB24, member RAS oncogene family	Rab24	2.099	<0.0001
nicotinamide nucleotide transhydrogenase	Nnt	2.098	<0.0001
modulator of apoptosis 1	Moap1	2.098	<0.0001
WD repeat domain 34	Wdr34	2.096	<0.0001
speckle-type POZ protein	Spop	2.095	<0.0001
copper chaperone for superoxide dismutase	Ccs	2.093	<0.0001
NADH dehydrogenase (ubiquinone) Fe-S protein 1	Ndufs1	2.092	<0.0001
Yip1 domain family, member 4	Yipf4	2.091	<0.0001
fasciculation and elongation protein zeta 2 (zygin II)	Fez2	2.091	<0.0001
hydroxyacylglutathione hydrolase-like	Haghl	2.088	<0.0001
syndecan 4	Sdc4	2.087	<0.0001
centrosomal protein 19	Cep19	2.087	<0.0001
catenin (cadherin associated protein), alpha 2	Ctnna2	2.086	<0.0001
DnaJ (Hsp40) homolog, subfamily C, member 25	Dnajc25	2.086	<0.0001
malignant T cell amplified sequence 1	Mcts1	2.084	<0.0001
KRR1, small subunit (SSU) processome component, homolog (yeast)	Krr1	2.084	<0.0001
WD repeat domain 55	Wdr55	2.083	<0.0001
zinc finger, C3HC type 1	Zc3hc1	2.083	<0.0001

KIF1 binding protein	Kif1bp	2.083	<0.0001
TNF receptor-associated protein 1	Trap1	2.082	<0.0001
cytochrome b-561 domain containing 2	Cyb561d2	2.081	<0.0001
nudix (nucleoside diphosphate linked moiety X)-type motif 10	Nudt10	2.079	0.0017
hexosaminidase B	Hexb	2.079	<0.0001
mitochondrial amidoxime reducing component 2	Marc2	2.079	<0.0001
GTP binding protein 4	Gtpbp4	2.078	<0.0001
tubulin polyglutamylase complex subunit 1	Tpgs1	2.078	<0.0001
coiled-coil-helix-coiled-coil-helix domain containing 6	Chchd6	2.074	<0.0001
breast cancer metastasis-suppressor 1-like	Brms1l	2.073	<0.0001
deoxyhypusine synthase	Dhps	2.073	<0.0001
spastic paraplegia 20, spartin (Troyer syndrome) homolog (human)	Spg20	2.073	<0.0001
glutaredoxin	Glrx	2.068	<0.0001
stromal cell derived factor 2	Sdf2	2.067	<0.0001
DnaJ (Hsp40) homolog, subfamily C, member 3	Dnajc3	2.067	<0.0001
non-SMC condensin II complex, subunit H2	Ncaph2	2.066	<0.0001
endoplasmic reticulum protein 29	Erp29	2.065	<0.0001
FERM domain containing 6	Frmd6	2.065	<0.0001
NADH dehydrogenase (ubiquinone) 1 beta subcomplex, 2	Ndufb2	2.065	<0.0001
pregnancy upregulated non-ubiquitously expressed CaM kinase	Pnck	2.065	<0.0001
START domain containing 7	Stard7	2.061	<0.0001
methionine sulfoxide reductase B1	Msrb1	2.060	<0.0001
nudix (nucleoside diphosphate linked moiety X)-type motif 18	Nudt18	2.058	<0.0001
dual specificity phosphatase 26 (putative)	Dusp26	2.057	<0.0001
ARP1 actin-related protein 1B, centractin beta	Actr1b	2.055	<0.0001
four and a half LIM domains 2	Fhl2	2.052	<0.0001
zinc finger, RAN-binding domain containing 1	Zranb1	2.052	<0.0001
tumor suppressor candidate 2	Tusc2	2.051	<0.0001
methylmalonyl-Coenzyme A mutase	Mut	2.049	<0.0001
ubiquinol-cytochrome c reductase complex assembly factor 1	Uqcc1	2.049	<0.0001
elongation factor 1 homolog (ELF1, S. cerevisiae)	Elof1	2.047	<0.0001
BRF2, subunit of RNA polymerase III transcription initiation factor, BRF1-like	Brf2	2.046	<0.0001
melanoma antigen, family D, 2	Maged2	2.046	<0.0001
ribosomal protein L37	Rpl37	2.045	<0.0001

poly-U binding splicing factor 60	Puf60	2.040	<0.0001
syntaxin 1A (brain)	Stx1a	2.040	<0.0001
acetyl-Coenzyme A carboxylase alpha	Acaca	2.036	<0.0001
ATP citrate lyase	Acly	2.036	<0.0001
ECSIT homolog (Drosophila)	Ecsit	2.036	<0.0001
necdin-like 2	Ndnl2	2.035	<0.0001
transmembrane protein 203	Tmem203	2.034	<0.0001
maestro heat-like repeat family member 7	Mroh7	2.032	<0.0001
carbonic anhydrase 5b, mitochondrial	Car5b	2.031	<0.0001
transcription elongation factor B (SIII), polypeptide 2	Tceb2	2.029	0.0002
phosphofructokinase, muscle	Pfkm	2.029	<0.0001
ring finger protein, LIM domain interacting	Rlim	2.029	<0.0001
YTH domain family 2	Ythdf2	2.028	<0.0001
family with sequence similarity 212, member B	Fam212b	2.028	<0.0001
NOP10 ribonucleoprotein	Nop10	2.028	<0.0001
vasoactive intestinal polypeptide	Vip	2.027	<0.0001
hexamethylene bis-acetamide inducible 1	Hexim1	2.027	<0.0001
peroxisomal biogenesis factor 5-like	Pex5l	2.027	<0.0001
lysine (K)-specific demethylase 1A	Kdm1a	2.026	<0.0001
glutathione S-transferase omega 1	Gsto1	2.025	<0.0001
lipoic acid synthetase	Lias	2.024	<0.0001
protease (prosome, macropain) 26S subunit, ATPase 1	Psmc1	2.024	<0.0001
peroxisomal biogenesis factor 7	Pex7	2.024	<0.0001
intraflagellar transport 27	Ift27	2.024	<0.0001
monoamine oxidase A	Maoa	2.024	<0.0001
angio-associated migratory protein	Aamp	2.021	<0.0001
aldehyde dehydrogenase family 1, subfamily A1	Aldh1a1	2.019	0.0002
transmembrane protein 86A	Tmem86a	2.019	<0.0001
dystrobrevin binding protein 1	Dtnbp1	2.018	<0.0001
WD repeat domain 12	Wdr12	2.018	<0.0001
choroidermia (RAB escort protein 1)	Chm	2.016	<0.0001
dihydrouridine synthase 1-like (S. cerevisiae)	Dus1l	2.016	<0.0001
transmembrane protein 159	Tmem159	2.016	<0.0001
WD repeat domain 54	Wdr54	2.015	<0.0001

peptidase domain containing associated with muscle regeneration 1	Pamr1	2.014	<0.0001
BRF1 homolog, subunit of RNA polymerase III transcription initiation factor IIIB (S. cerevisiae)	Brf1	2.014	<0.0001
Eph receptor B6	Ephb6	2.013	<0.0001
myotubularin related protein 4	Mtmr4	2.012	<0.0001
tudor domain containing 7	Tdrd7	2.012	<0.0001
clavesin 2	Clvs2	2.012	<0.0001
cytidine monophospho-N-acetylneuraminic acid synthetase	Cmas	2.011	<0.0001
solute carrier family 35, member E3	Slc35e3	2.011	<0.0001
family with sequence similarity 193, member A	Fam193a	2.008	<0.0001
ras homolog gene family, member B	Rhob	2.007	<0.0001
FtsJ homolog 3 (E. coli)	Ftsj3	2.006	<0.0001
presenilin associated, rhomboid-like	Parl	2.006	<0.0001
ST3 beta-galactoside alpha-2,3-sialyltransferase 5	St3gal5	2.005	<0.0001
transmembrane protein 129	Tmem129	2.004	<0.0001
exostoses (multiple) 1	Ext1	2.004	<0.0001
Rac GTPase-activating protein 1	Racgap1	2.004	<0.0001
intraflagellar transport 57	Ift57	2.003	<0.0001
processing of precursor 7, ribonuclease P family, (S. cerevisiae)	Pop7	2.002	<0.0001
PDZ and LIM domain 5	Pdlim5	2.002	<0.0001
Ras-related GTP binding A	Rraga	2.000	<0.0001
adenylosuccinate lyase	Adsl	1.997	<0.0001
translocase of outer mitochondrial membrane 70 homolog A (yeast)	Tomm70a	1.997	<0.0001
SDE2 telomere maintenance homolog (S. pombe)	Sde2	1.996	<0.0001
NADH dehydrogenase (ubiquinone) 1 alpha subcomplex, 9	Ndufa9	1.996	<0.0001
nucleosome assembly protein 1-like 4	Nap1l4	1.995	<0.0001
zinc finger protein 871	Zfp871	1.994	<0.0001
transmembrane protein 246	Tmem246	1.994	<0.0001
ubiquitin-like 5	Ubl5	1.993	<0.0001
protein arginine N-methyltransferase 8	Prmt8	1.992	<0.0001
hydroxysteroid (17-beta) dehydrogenase 4	Hsd17b4	1.992	<0.0001
DnaJ (Hsp40) homolog, subfamily C, member 11	Dnajc11	1.991	<0.0001
5-nucleotidase, cytosolic III	Nt5c3	1.989	<0.0001
polymerase (RNA) III (DNA directed) polypeptide H	Polr3h	1.987	<0.0001

potassium channel, subfamily K, member 1	Kcnk1	1.987	<0.0001
proteasome (prosome, macropain) 26S subunit, non-ATPase, 3	Psmd3	1.986	<0.0001
arginine/serine-rich coiled-coil 1	Rsrc1	1.982	<0.0001
translocase of inner mitochondrial membrane 13	Timm13	1.982	<0.0001
charged multivesicular body protein 1B	Chmp1b	1.982	<0.0001
coproporphyrinogen oxidase	Cpox	1.981	<0.0001
eukaryotic translation initiation factor 4A3	Eif4a3	1.981	<0.0001
sorting nexin family member 30	Snx30	1.981	<0.0001
blocked early in transport 1 homolog (S. cerevisiae)-like	Bet1l	1.979	<0.0001
fucose mutarotase	Fuom	1.978	<0.0001
eukaryotic translation initiation factor 4A2	Eif4a2	1.974	<0.0001
PAK1 interacting protein 1	Pak1ip1	1.973	<0.0001
SEC14 and spectrin domains 1	Sestd1	1.971	<0.0001
leucine rich repeat containing 24	Lrrc24	1.969	<0.0001
glutamate dehydrogenase 1	Glud1	1.967	<0.0001
disrupted in renal carcinoma 2 (human)	Dirc2	1.967	<0.0001
DEAH (Asp-Glu-Ala-His) box polypeptide 15	Dhx15	1.965	<0.0001
guanylate kinase 1	Guk1	1.964	<0.0001
F-box protein 42	Fbxo42	1.964	<0.0001
mortality factor 4 like 1	Morf4l1	1.962	<0.0001
DEAD (Asp-Glu-Ala-Asp) box polypeptide 20	Ddx20	1.961	<0.0001
dehydrodolichyl diphosphate synthase	Dhdds	1.961	<0.0001
sterol carrier protein 2, liver	Scp2	1.958	<0.0001
G protein-coupled receptor 26	Gpr26	1.957	<0.0001
ring finger protein 11	Rnf11	1.957	<0.0001
zinc finger, CCHC domain containing 18	Zcchc18	1.957	<0.0001
TROVE domain family, member 2	Trove2	1.957	<0.0001
chromobox 1	Cbx1	1.956	<0.0001
adaptor-related protein complex 3, mu 2 subunit	Ap3m2	1.955	<0.0001
coenzyme Q10 homolog B (S. cerevisiae)	Coq10b	1.952	<0.0001
carbonic anhydrase 8	Car8	1.951	<0.0001
acyl-Coenzyme A dehydrogenase family, member 9	Acad9	1.949	<0.0001
tripartite motif-containing 23	Trim23	1.945	<0.0001
abhydrolase domain containing 13	Abhd13	1.944	<0.0001

elongator acetyltransferase complex subunit 3	Elp3	1.943	<0.0001
histone cluster 2, H4	Hist2h4	1.941	0.0007
proteasome (prosome, macropain) subunit, alpha type 2	Psma2	1.940	<0.0001
guanine nucleotide binding protein (G protein), alpha inhibiting 3	Gnai3	1.939	<0.0001
homocysteine-inducible, endoplasmic reticulum stress-inducible, ubiquitin-like domain member 1	Herpud1	1.938	<0.0001
RuvB-like protein 2	Ruvbl2	1.937	<0.0001
cytidine and dCMP deaminase domain containing 1	Cdadc1	1.935	<0.0001
splicing factor 3B, subunit 6	Sf3b6	1.933	<0.0001
H2A histone family, member X	H2afx	1.932	<0.0001
pleiotropic regulator 1, PRL1 homolog (Arabidopsis)	Plrg1	1.928	<0.0001
importin 5	Ipo5	1.926	<0.0001
COP9 (constitutive photomorphogenic) homolog, subunit 5 (Arabidopsis thaliana)	Cops5	1.925	<0.0001
mitochondrial ribosomal protein S25	Mrps25	1.925	<0.0001
methyl-CpG binding domain protein 3	Mbd3	1.922	<0.0001
eukaryotic translation initiation factor 2B, subunit 5 epsilon	Eif2b5	1.920	<0.0001
ribosomal protein SA	Rpsa	1.920	<0.0001
TSC22 domain family, member 4	Tsc22d4	1.918	<0.0001
sphingosine phosphate lyase 1	Sgpl1	1.916	<0.0001
LIM domain only 4	Lmo4	1.915	<0.0001
patatin-like phospholipase domain containing 8	Pnpla8	1.915	<0.0001
MIS12 homolog (yeast)	Mis12	1.911	<0.0001
protein-kinase, interferon-inducible double stranded RNA dependent inhibitor, repressor of (P58 repressor)	Prkrir	1.910	<0.0001
cerebellin 2 precursor protein	Cbln2	1.910	<0.0001
ATP-binding cassette, sub-family F (GCN20), member 3	Abcf3	1.908	<0.0001
Yip1 domain family, member 1	Yipf1	1.907	<0.0001
glutaminyl-tRNA synthetase	Qars	1.906	<0.0001
TBC1 domain family, member 14	Tbc1d14	1.906	<0.0001
insulin-like growth factor binding protein 6	Igfbp6	1.906	<0.0001
ubiquitin-conjugating enzyme E2 variant 2	Ube2v2	1.904	<0.0001
reticulon 2 (Z-band associated protein)	Rtn2	1.903	<0.0001
dolichol-phosphate (beta-D) mannosyltransferase 1	Dpm1	1.903	<0.0001
ATP synthase, H+ transporting, mitochondrial F0 complex, subunit C3 (subunit 9)	Atp5g3	1.901	<0.0001

solute carrier family 37 (glycerol-3-phosphate transporter), member 3	Slc37a3	1.901	<0.0001
dehydrogenase E1 and transketolase domain containing 1	Dhtkd1	1.900	<0.0001
Yip1 interacting factor homolog A (S. cerevisiae)	Yif1a	1.900	<0.0001
diacylglycerol kinase, delta	Dgkd	1.900	<0.0001
nudix (nucleoside diphosphate linked moiety X)-type motif 16-like 1	Nudt16l1	1.898	<0.0001
negative elongation factor complex member C/D, Th1l	Nelfcd	1.898	<0.0001
upstream transcription factor 1	Usf1	1.898	<0.0001
euchromatic histone lysine N-methyltransferase 2	Ehmt2	1.897	<0.0001
FSHD region gene 1	Frg1	1.896	<0.0001
zinc finger and SCAN domain containing 21	Zscan21	1.896	<0.0001
potassium channel, subfamily K, member 2	Kcnk2	1.896	<0.0001
solute carrier family 6 (neurotransmitter transporter, L-proline), member 7	Slc6a7	1.895	<0.0001
transketolase	Tkt	1.895	<0.0001
Myb/SANT-like DNA-binding domain containing 4 with coiled-coils	Msantd4	1.894	<0.0001
RNA binding motif protein 8a	Rbm8a	1.894	<0.0001
biogenesis of lysosomal organelles complex-1, subunit 4, cappuccino	Bloc1s4	1.893	<0.0001
synaptosomal-associated protein, 47	Snap47	1.893	<0.0001
trafficking protein particle complex 13	Trappc13	1.892	<0.0001
protein phosphatase 1F (PP2C domain containing)	Ppm1f	1.892	<0.0001
TBC1 domain family, member 13	Tbc1d13	1.891	<0.0001
high mobility group nucleosomal binding domain 3	Hmgn3	1.891	<0.0001
transmembrane protein 126B	Tmem126b	1.890	<0.0001
cytochrome c oxidase subunit IV isoform 1	Cox4i1	1.890	<0.0001
Ca2+-dependent activator protein for secretion 2	Cadps2	1.889	<0.0001
UBX domain protein 6	Ubxn6	1.888	<0.0001
vesicle-associated membrane protein 4	Vamp4	1.888	<0.0001
calumenin	Calu	1.888	<0.0001
translocase of inner mitochondrial membrane 22	Timm22	1.887	<0.0001
gem (nuclear organelle) associated protein 4	Gemin4	1.887	0.0008
glutathione S-transferase, mu 5	Gstm5	1.886	<0.0001
3-hydroxy-3-methylglutaryl-Coenzyme A lyase	Hmgcl	1.886	<0.0001
kelch repeat and BTB (POZ) domain containing 4	Kbtbd4	1.886	<0.0001
actin related protein 2/3 complex, subunit 1A	Arpc1a	1.885	<0.0001
3-oxoacid CoA transferase 1	Oxct1	1.884	<0.0001

RWD domain containing 1	Rwdd1	1.883	<0.0001
nucleosome assembly protein 1-like 5	Nap1l5	1.882	<0.0001
neuropeptide Y receptor Y1	Npy1r	1.881	<0.0001
CD320 antigen	Cd320	1.880	<0.0001
COP9 (constitutive photomorphogenic) homolog, subunit 8 (Arabidopsis thaliana)	Cops8	1.880	<0.0001
eukaryotic translation initiation factor 4E member 3	Eif4e3	1.879	<0.0001
myocilin	Myoc	1.879	<0.0001
potassium inwardly-rectifying channel, subfamily J, member 2	Kcnj2	1.879	<0.0001
phosphoglucomutase 2-like 1	Pgm2l1	1.879	<0.0001
claudin 12	Cldn12	1.879	<0.0001
neurolysin (metallopeptidase M3 family)	Nln	1.877	<0.0001
solute carrier family 35 (CMP-sialic acid transporter), member 1	Slc35a1	1.875	<0.0001
folliculin	Flcn	1.875	<0.0001
myotubularin related protein 6	Mtmr6	1.874	<0.0001
DEAH (Asp-Glu-Ala-His) box polypeptide 40	Dhx40	1.873	<0.0001
ribonuclease, RNase K	Rnasek	1.872	<0.0001
proteasome (prosome, macropain) 26S subunit, non-ATPase, 11	Psmd11	1.871	<0.0001
family with sequence similarity 73, member B	Fam73b	1.870	<0.0001
glutamine fructose-6-phosphate transaminase 1	Gfpt1	1.867	<0.0001
PRP6 pre-mRNA splicing factor 6 homolog (yeast)	Prpf6	1.866	<0.0001
Kruppel-like factor 15	Klf15	1.866	<0.0001
signal recognition particle 19	Srp19	1.863	<0.0001
basic helix-loop-helix family, member e41	Bhlhe41	1.862	<0.0001
triple QxxK/R motif containing	Triqk	1.862	<0.0001
complement component 1, q subcomponent, C chain	C1qc	1.862	<0.0001
lectin, galactose binding, soluble 8	Lgals8	1.861	<0.0001
estrogen related receptor, alpha	Esrra	1.860	<0.0001
ankyrin repeat and MYND domain containing 2	Ankmy2	1.859	<0.0001
alcohol dehydrogenase 5 (class III), chi polypeptide	Adh5	1.858	<0.0001
elongator acetyltransferase complex subunit 5	Elp5	1.858	<0.0001
methylphosphate capping enzyme	Mepce	1.858	<0.0001
COMM domain containing 8	Commd8	1.857	<0.0001
cornichon homolog 4 (Drosophila)	Cnih4	1.855	<0.0001
eukaryotic translation initiation factor 2, subunit 2 (beta)	Eif2s2	1.854	<0.0001

ribosomal protein L21	Rpl21	1.850	<0.0001
thioredoxin-like 1	Txnl1	1.850	<0.0001
pannexin 1	Panx1	1.848	<0.0001
CD53 antigen	Cd53	1.847	<0.0001
DEAD (Asp-Glu-Ala-Asp) box polypeptide 25	Ddx25	1.845	<0.0001
family with sequence similarity 185, member A	Fam185a	1.844	<0.0001
diacylglycerol kinase, epsilon	Dgke	1.844	<0.0001
heterogeneous nuclear ribonucleoprotein H1	Hnrnph1	1.844	<0.0001
transmembrane and ubiquitin-like domain containing 2	Tmub2	1.844	<0.0001
tetraspanin 12	Tspan12	1.844	<0.0001
PHD finger protein 6	Phf6	1.842	<0.0001
stomatin-like 1	Stoml1	1.842	<0.0001
tumor suppressing subtransferable candidate 1	Tssc1	1.840	<0.0001
pleiotrophin	Ptn	1.839	<0.0001
ring finger protein 219	Rnf219	1.836	<0.0001
charged multivesicular body protein 2A	Chmp2a	1.835	<0.0001
LysM, putative peptidoglycan-binding, domain containing 1	Lysmd1	1.835	<0.0001
structure specific recognition protein 1	Ssrp1	1.835	<0.0001
zinc finger (CCCH type), RNA binding motif and serine/arginine rich 1	Zrsr1	1.834	<0.0001
pyridoxal (pyridoxine, vitamin B6) kinase	Pdxk	1.834	<0.0001
intraflagellar transport 46	Ift46	1.833	<0.0001
NADH dehydrogenase (ubiquinone) 1 beta subcomplex, 11	Ndufb11	1.832	<0.0001
family with sequence similarity 126, member A	Fam126a	1.832	<0.0001
cytokine receptor-like factor 3	Crlf3	1.830	<0.0001
ADP-ribosylation factor-like 2 binding protein	Arl2bp	1.830	<0.0001
BCL2/adenovirus E1B interacting protein 3-like	Bnip3l	1.830	<0.0001
a disintegrin and metallopeptidase domain 10	Adam10	1.829	<0.0001
exocyst complex component 5	Exoc5	1.829	<0.0001
CDP-diacylglycerol synthase 1	Cds1	1.828	<0.0001
DDB1 and CUL4 associated factor 8	Dcaf8	1.828	<0.0001
anaphase promoting complex subunit 13	Anapc13	1.827	<0.0001
sulfatase modifying factor 1	Sumf1	1.826	<0.0001
leptin receptor overlapping transcript	Leprot	1.826	<0.0001

SWI/SNF related, matrix associated, actin dependent regulator of chromatin, subfamily a, member 1	Smarca1	1.826	<0.0001
transmembrane protein 143	Tmem143	1.826	<0.0001
H3 histone, family 3B	H3f3b	1.825	<0.0001
nuclear receptor subfamily 2, group F, member 1	Nr2f1	1.824	<0.0001
TBC1 domain family, member 24	Tbc1d24	1.824	<0.0001
gem (nuclear organelle) associated protein 6	Gemin6	1.823	<0.0001
torsin family 1, member B	Tor1b	1.822	<0.0001
hepatoma-derived growth factor	Hdgf	1.822	<0.0001
RNA binding motif protein 45	Rbm45	1.822	<0.0001
arginine/serine-rich coiled-coil 2	Rsrc2	1.822	<0.0001
limb region 1	Lmbr1	1.821	<0.0001
transmembrane protein 101	Tmem101	1.821	<0.0001
emopamil binding protein-like	Ebpl	1.820	<0.0001
SEC13 homolog, nuclear pore and COPII coat complex component	Sec13	1.819	<0.0001
F-box protein 2	Fbxo2	1.816	<0.0001
t-complex 11 (mouse) like 2	Tcp11l2	1.816	<0.0001
chaperonin containing Tcp1, subunit 2 (beta)	Cct2	1.815	<0.0001
family with sequence similarity 110, member B	Fam110b	1.814	<0.0001
zinc finger protein 790	Zfp790	1.814	<0.0001
seven in absentia 1A	Siah1a	1.813	<0.0001
ferredoxin 1	Fdx1	1.813	<0.0001
CAS1 domain containing 1	Casd1	1.812	<0.0001
replication protein A1	Rpa1	1.810	<0.0001
mutS homolog 2 (E. coli)	Msh2	1.810	<0.0001
glycyl-tRNA synthetase	Gars	1.809	<0.0001
ubiquitin-fold modifier conjugating enzyme 1	Ufc1	1.809	<0.0001
ubiquitin-conjugating enzyme E2N	Ube2n	1.808	<0.0001
CaM kinase-like vesicle-associated	Camkv	1.808	<0.0001
par-6 family cell polarity regulator alpha	Pard6a	1.806	<0.0001
SURP and G patch domain containing 1	Sugp1	1.805	<0.0001
ATP-binding cassette, sub-family F (GCN20), member 2	Abcf2	1.805	<0.0001
intercellular adhesion molecule 5, telencephalin	Icam5	1.805	<0.0001
tubulin-specific chaperone C	Tbcc	1.804	<0.0001

v-ral simian leukemia viral oncogene homolog B (ras related)	Ralb	1.803	<0.0001
acetyl-Coenzyme A acetyltransferase 1	Acat1	1.802	<0.0001
ilvB (bacterial acetolactate synthase)-like	Ilvbl	1.801	<0.0001
ADP-ribosylation factor-like 6 interacting protein 6	Arl6ip6	1.801	<0.0001
tubulin-specific chaperone d	Tbcd	1.801	<0.0001
mitochondrial ribosomal protein S10	Mrps10	1.800	0.0008
zinc finger protein 869	Zfp869	1.799	<0.0001
F-box and leucine-rich repeat protein 5	Fbxl5	1.797	<0.0001
penta-EF hand domain containing 1	Pef1	1.796	<0.0001
transmembrane protein 74	Tmem74	1.796	0.0001
PHD finger protein 23	Phf23	1.796	<0.0001
PDZ domain containing 4	Pdzd4	1.795	<0.0001
Bardet-Biedl syndrome 1 (human)	Bbs1	1.795	<0.0001
DnaJ (Hsp40) homolog, subfamily C, member 18	Dnajc18	1.794	<0.0001
melanoma associated antigen (mutated) 1	Mum1	1.794	<0.0001
AU RNA binding protein/enoyl-coenzyme A hydratase	Auh	1.794	<0.0001
zinc finger and BTB domain containing 18	Zbtb18	1.793	<0.0001
armadillo repeat containing 1	Armc1	1.793	<0.0001
mannoside acetylglucosaminyltransferase 5	Mgat5	1.792	<0.0001
zinc finger protein 60	Zfp60	1.792	<0.0001
protocadherin beta 12	Pcdhb12	1.792	<0.0001
monocyte to macrophage differentiation-associated	Mmd	1.788	<0.0001
RNA binding motif protein 25	Rbm25	1.788	<0.0001
mitogen-activated protein kinase 8	Mapk8	1.788	<0.0001
vitronectin	Vtn	1.788	<0.0001
protocadherin beta 7	Pcdhb7	1.787	<0.0001
anaphase promoting complex subunit 16	Anapc16	1.786	<0.0001
ecotropic viral integration site 5 like	Evi5l	1.785	<0.0001
chaperonin containing Tcp1, subunit 4 (delta)	Cct4	1.784	<0.0001
programmed cell death 5	Pdcd5	1.783	0.0005
flap structure specific endonuclease 1	Fen1	1.783	<0.0001
sterile alpha motif domain containing 8	Samd8	1.783	<0.0001
dual serine/threonine and tyrosine protein kinase	Dstyk	1.782	<0.0001
NADH dehydrogenase (ubiquinone) 1 alpha subcomplex assembly factor 7	Ndufaf7	1.780	<0.0001

G protein-coupled receptor 107	Gpr107	1.780	<0.0001
translin-associated factor X	Tsnax	1.780	<0.0001
protein O-linked mannose beta 1,2-N-acetylglucosaminyltransferase	Pomgnt1	1.780	<0.0001
insulin-like growth factor binding protein 4	Igfbp4	1.779	<0.0001
mannoside acetylglucosaminyltransferase 1	Mgat1	1.779	<0.0001
family with sequence similarity 134, member C	Fam134c	1.779	<0.0001
hydroxysteroid dehydrogenase like 1	Hsdl1	1.778	<0.0001
interleukin 34	Il34	1.778	<0.0001
transcription elongation factor A (SII), 2	Tcea2	1.777	<0.0001
dynactin 2	Dctn2	1.777	<0.0001
milk fat globule-EGF factor 8 protein	Mfge8	1.776	<0.0001
cyclin D-type binding-protein 1	Ccndbp1	1.776	<0.0001
ubiquitin specific peptidase 29	Usp29	1.775	<0.0001
troponin C, cardiac/slow skeletal	Tnnc1	1.774	<0.0001
CDGSH iron sulfur domain 2	Cisd2	1.774	<0.0001
mitochondrial ribosomal protein L20	Mrpl20	1.774	<0.0001
Slc10a3-Ubl4a readthrough transcript (NMD candidate)	SlcUbl4a	1.774	<0.0001
epoxide hydrolase 1, microsomal	Ephx1	1.774	<0.0001
heterogeneous nuclear ribonucleoprotein M	Hnrnpm	1.774	<0.0001
protein phosphatase 1, regulatory (inhibitor) subunit 11	Ppp1r11	1.771	<0.0001
translocation associated membrane protein 1-like 1	Tram1l1	1.771	<0.0001
SYF2 homolog, RNA splicing factor (S. cerevisiae)	Syf2	1.771	<0.0001
fizzy/cell division cycle 20 related 1 (Drosophila)	Fzr1	1.769	<0.0001
tubulin, alpha 1A	Tuba1a	1.768	<0.0001
transmembrane protein 18	Tmem18	1.766	<0.0001
Trp53 induced glycolysis repulatory phosphatase	Tigar	1.766	<0.0001
threonyl-tRNA synthetase-like 2	Tarsl2	1.766	<0.0001
hypoxanthine guanine phosphoribosyl transferase	Hprt	1.764	<0.0001
triosephosphate isomerase 1	Tpi1	1.762	<0.0001
K(lysine) acetyltransferase 5	Kat5	1.761	<0.0001
X-ray repair complementing defective repair in Chinese hamster cells 6	Xrcc6	1.761	<0.0001
solute carrier family 25 (mitochondrial carrier, adenine nucleotide translocator), member 4	Slc25a4	1.760	<0.0001
non imprinted in Prader-Willi/Angelman syndrome 1 homolog (human)	Nipa1	1.758	<0.0001

immediate early response 5	Ier5	1.758	<0.0001
regulator of G-protein signaling 17	Rgs17	1.757	<0.0001
legumain	Lgmn	1.757	<0.0001
ubiquitin protein ligase E3A	Ube3a	1.757	<0.0001
RNA methyltransferase like 1	Rnmtl1	1.756	<0.0001
single-pass membrane protein with aspartate rich tail 1	Smdt1	1.756	<0.0001
neuraminidase 1	Neu1	1.756	<0.0001
tropomyosin 1, alpha	Tpm1	1.756	<0.0001
NADH dehydrogenase (ubiquinone) 1 alpha subcomplex, assembly factor 4	Ndufaf4	1.755	<0.0001
phosphoglucomutase 3	Pgm3	1.755	<0.0001
negative elongation factor complex member E, Rdbp	Nelfe	1.754	<0.0001
NADH dehydrogenase (ubiquinone) Fe-S protein 6	Ndufs6	1.752	0.0049
ubiquitin-conjugating enzyme E2H	Ube2h	1.752	<0.0001
transmembrane protein 208	Tmem208	1.752	<0.0001
retinol dehydrogenase 11	Rdh11	1.752	<0.0001
replication protein A2	Rpa2	1.752	<0.0001
eukaryotic translation initiation factor 3, subunit I	Eif3i	1.750	<0.0001
alanyl-tRNA synthetase domain containing 1	Aarsd1	1.750	<0.0001
tubulin polymerization promoting protein	Tppp	1.749	<0.0001
polycomb group ring finger 5	Pcgf5	1.749	<0.0001
zinc finger protein 512	Zfp512	1.749	<0.0001
glycerol-3-phosphate acyltransferase, mitochondrial	Gpam	1.749	<0.0001
ELAV (embryonic lethal, abnormal vision, Drosophila)-like 2 (Hu antigen B)	Elavl2	1.749	<0.0001
small G protein signaling modulator 3	Sgsm3	1.749	<0.0001
proteasome (prosome, macropain) 26S subunit, non-ATPase, 10	Psmd10	1.748	<0.0001
acyl-Coenzyme A oxidase 1, palmitoyl	Acox1	1.748	<0.0001
general transcription factor IIH, polypeptide 5	Gtf2h5	1.746	<0.0001
farnesyltransferase, CAAX box, alpha	Fnta	1.745	<0.0001
EP300 interacting inhibitor of differentiation 1	Eid1	1.744	<0.0001
nuclear prelamin A recognition factor	Narf	1.744	<0.0001
nipsnap homolog 3B (C. elegans)	Nipsnap3b	1.743	<0.0001
transmembrane emp24 protein transport domain containing 9	Tmed9	1.742	<0.0001
OTU deubiquitinase with linear linkage specificity	Otulin	1.741	<0.0001
tetratricopeptide repeat domain 9C	Ttc9c	1.737	<0.0001

ets variant 5	Etv5	1.737	<0.0001
mitochondrial ribosomal protein S28	Mrps28	1.734	<0.0001
trans-golgi network vesicle protein 23B	Tvp23b	1.734	<0.0001
transmembrane protein 147	Tmem147	1.733	<0.0001
sorting nexin 16	Snx16	1.733	<0.0001
BCL2-associated X protein	Bax	1.732	<0.0001
small integral membrane protein 10 like 2A	Smim10l2a	1.731	<0.0001
mitogen-activated protein kinase kinase 6	Map2k6	1.731	<0.0001
metallothionein 1	Mt1	1.731	<0.0001
AT rich interactive domain 3C (BRIGHT-like)	Arid3c	1.730	<0.0001
thioredoxin-related transmembrane protein 1	Tmx1	1.730	<0.0001
potassium channel tetramerisation domain containing 5	Kctd5	1.730	<0.0001
parvalbumin	Pvalb	1.728	0.0001
family with sequence similarity 76, member A	Fam76a	1.726	<0.0001
hydroxysteroid dehydrogenase like 2	Hsdl2	1.726	<0.0001
enoyl coenzyme A hydratase 1, peroxisomal	Ech1	1.725	<0.0001
yippee-like 5 (Drosophila)	Ypel5	1.725	<0.0001
major facilitator superfamily domain containing 1	Mfsd1	1.723	<0.0001
protein phosphatase 1G (formerly 2C), magnesium-dependent, gamma isoform	Ppm1g	1.723	<0.0001
solute carrier family 39 (zinc transporter), member 10	Slc39a10	1.723	<0.0001
quinoid dihydropteridine reductase	Qdpr	1.723	<0.0001
interferon, alpha-inducible protein 27	Ifi27	1.722	<0.0001
phosphodiesterase 12	Pde12	1.721	<0.0001
RAD50 interactor 1	Rint1	1.721	<0.0001
ephrin B2	Efnb2	1.720	<0.0001
Werner helicase interacting protein 1	Wrnip1	1.720	<0.0001
aldolase A, fructose-bisphosphate	Aldoa	1.719	<0.0001
ankyrin repeat domain 34C	Ankrd34c	1.719	0.0003
mediator complex subunit 9	Med9	1.718	<0.0001
GPN-loop GTPase 1	Gpn1	1.718	<0.0001
SMAD family member 1	Smad1	1.717	<0.0001
family with sequence similarity 114, member A2	Fam114a2	1.717	<0.0001
calcium binding protein 1	Cabp1	1.716	<0.0001
activator of basal transcription 1	Abt1	1.715	<0.0001

lysine (K)-specific demethylase 5A	Kdm5a	1.715	<0.0001
enoyl-Coenzyme A delta isomerase 1	Eci1	1.714	<0.0001
phosphatidylinositol glycan anchor biosynthesis, class F	Pigf	1.714	<0.0001
fatty acid binding protein 3, muscle and heart	Fabp3	1.714	0.0004
cap methyltransferase 1	Cmtr1	1.714	<0.0001
neuroguidin, EIF4E binding protein	Ngdn	1.713	<0.0001
microfibrillar-associated protein 1B	Mfap1b	1.712	<0.0001
canopy 4 homolog (zebrafish)	Cnpy4	1.712	<0.0001
BLOC-1 related complex subunit 5	Borcs5	1.711	<0.0001
endosulfine alpha	Ensa	1.711	<0.0001
succinate dehydrogenase complex, subunit B, iron sulfur (Ip)	Sdhb	1.710	<0.0001
kit oncogene	Kit	1.709	<0.0001
N-acetylglucosamine kinase	Nagk	1.709	<0.0001
armadillo repeat containing, X-linked 5	Armcx5	1.707	<0.0001
phosphoglycerate mutase family member 5	Pgam5	1.706	<0.0001
nuclear receptor subfamily 1, group D, member 2	Nr1d2	1.706	<0.0001
kelch-like 26	Klhl26	1.706	<0.0001
glycosyltransferase 8 domain containing 2	Glt8d2	1.705	<0.0001
lysyl-tRNA synthetase	Kars	1.705	<0.0001
guanosine monophosphate reductase 2	Gmpr2	1.704	<0.0001
mitochondrial GTPase 1 homolog (S. cerevisiae)	Mtg1	1.704	<0.0001
transmembrane emp24 domain containing 3	Tmed3	1.703	<0.0001
DnaJ (Hsp40) homolog, subfamily C, member 9	Dnajc9	1.703	<0.0001
tRNA nucleotidyl transferase, CCA-adding, 1	Trnt1	1.702	<0.0001
gelsolin	Gsn	1.701	<0.0001
WAPL cohesin release factor	Wapl	1.701	<0.0001
calcium and integrin binding family member 2	Cib2	1.701	<0.0001
Kruppel-like factor 6	Klf6	1.700	<0.0001
inturned planar cell polarity effector homolog (Drosophila)	Intu	1.700	<0.0001
ring finger protein 141	Rnf141	1.699	<0.0001
tranlocase of inner mitochondrial membrane 21	Timm21	1.698	<0.0001
transmembrane protein 41a	Tmem41a	1.698	<0.0001
cyclin G2	Ccng2	1.697	<0.0001
mitochondrial ribosomal protein S23	Mrps23	1.697	<0.0001

histone cluster 3, H2ba	Hist3h2ba	1.697	<0.0001
polyglutamine binding protein 1	Pqbp1	1.696	<0.0001
ribonucleotide reductase M2 B (TP53 inducible)	Rrm2b	1.696	<0.0001
cleavage and polyadenylation specific factor 2	Cpsf2	1.696	<0.0001
transforming growth factor, beta receptor I	Tgfbr1	1.695	<0.0001
neuropeptide S receptor 1	Npsr1	1.694	<0.0001
centrin 4	Cetn4	1.694	<0.0001
trafficking protein particle complex 2	Trappc2	1.693	<0.0001
exostoses (multiple)-like 2	Extl2	1.693	<0.0001
FK506 binding protein 3	Fkbp3	1.693	<0.0001
UTP14, U3 small nucleolar ribonucleoprotein, homolog B (yeast)	Utp14b	1.692	<0.0001
eukaryotic translation elongation factor 1 alpha 2	Eef1a2	1.691	<0.0001
coiled-coil domain containing 85B	Ccdc85b	1.691	<0.0001
CGG triplet repeat binding protein 1	Cggbp1	1.691	<0.0001
zinc finger protein 114	Zfp114	1.690	<0.0001
transmembrane protein 205	Tmem205	1.690	<0.0001
mitochondrial ribosomal protein L38	Mrpl38	1.690	<0.0001
retinoid X receptor beta	Rxrb	1.688	<0.0001
mitotic spindle organizing protein 2	Mzt2	1.688	<0.0001
lipid droplet associated hydrolase	Ldah	1.687	<0.0001
peptidase (mitochondrial processing) alpha	Pmpca	1.686	<0.0001
ATP synthase, H+ transporting, mitochondrial F0 complex, subunit F2	Atp5j2	1.685	0.0014
transformation related protein 53 inducible nuclear protein 1	Trp53inp1	1.684	<0.0001
ADP-ribosylation factor interacting protein 2	Arfip2	1.684	<0.0001
protein arginine N-methyltransferase 7	Prmt7	1.684	<0.0001
calmodulin 3	Calm3	1.683	<0.0001
protein phosphatase 1, regulatory (inhibitor) subunit 16A	Ppp1r16a	1.683	<0.0001
bone morphogenic protein/retinoic acid inducible neural-specific 2	Brinp2	1.682	<0.0001
protein phosphatase 1, regulatory (inhibitor) subunit 8	Ppp1r8	1.682	<0.0001
IZUMO family member 4	Izumo4	1.682	<0.0001
coatomer protein complex, subunit epsilon	Cope	1.682	<0.0001
GTPase activating protein and VPS9 domains 1	Gapvd1	1.681	<0.0001
coatomer protein complex, subunit beta 2 (beta prime)	Copb2	1.681	<0.0001

pterin 4 alpha carbinolamine dehydratase/dimerization cofactor of hepatocyte nuclear factor 1 alpha (TCF1) 2	Pcbd2	1.681	<0.0001
dynein, cytoplasmic 1 light intermediate chain 2	Dync1li2	1.680	<0.0001
ring finger protein 167	Rnf167	1.680	<0.0001
ATPase, H+ transporting, lysosomal V0 subunit B	Atp6v0b	1.679	<0.0001
membrane-associated ring finger (C3HC4) 8	March8	1.679	<0.0001
HECT domain and ankyrin repeat containing, E3 ubiquitin protein ligase 1	Hace1	1.678	<0.0001
calcium/calmodulin-dependent protein kinase I	Camk1	1.676	<0.0001
missing oocyte, meiosis regulator, homolog (Drosophila)	Mios	1.676	<0.0001
heterogeneous nuclear ribonucleoprotein L	Hnrnpl	1.675	<0.0001
pleckstrin homology domain containing, family B (evectins) member 2	Plekhb2	1.674	<0.0001
ankyrin repeat and BTB (POZ) domain containing 1	Abtb1	1.674	<0.0001
PDZ domain containing 11	Pdzd11	1.673	<0.0001
transaldolase 1	Taldo1	1.673	<0.0001
phosphatidylinositol 4-kinase type 2 alpha	Pi4k2a	1.673	<0.0001
limb-bud and heart	Lbh	1.673	<0.0001
apolipoprotein E	Apoe	1.673	<0.0001
Mid1 interacting protein 1 (gastrulation specific G12-like (zebrafish))	Mid1ip1	1.672	<0.0001
transmembrane protein 132A	Tmem132a	1.672	<0.0001
methyltransferase like 9	Mettl9	1.671	<0.0001
epilepsy, progressive myoclonic epilepsy, type 2 gene alpha	Epm2a	1.671	<0.0001
cold shock domain containing E1, RNA binding	Csde1	1.671	<0.0001
mediator complex subunit 10	Med10	1.670	<0.0001
timeless interacting protein	Tipin	1.670	<0.0001
negative elongation factor complex member B	Nelfb	1.669	<0.0001
GDP-mannose pyrophosphorylase B	Gmppb	1.669	<0.0001
ring finger protein 112	Rnf112	1.668	<0.0001
megakaryocyte-associated tyrosine kinase	Matk	1.667	<0.0001
septin 4	Sept4	1.666	<0.0001
coenzyme Q9 homolog (yeast)	Coq9	1.666	<0.0001
WD repeat domain 3	Wdr3	1.665	<0.0001
sodium channel modifier 1	Scnm1	1.665	<0.0001
profilin family, member 4	Pfn4	1.665	<0.0001
Luc7 homolog (S. cerevisiae)-like	Luc7l	1.664	<0.0001

protein phosphatase 4, catalytic subunit	Ppp4c	1.663	<0.0001
mitotic spindle organizing protein 1	Mzt1	1.663	<0.0001
T cell leukemia translocation altered gene	Tcta	1.663	<0.0001
MOB family member 4, phocein	Mob4	1.662	<0.0001
epoxide hydrolase 4	Ephx4	1.662	<0.0001
CDKN2A interacting protein N-terminal like	Cdkn2aipnl	1.661	<0.0001
zinc finger protein 426	Zfp426	1.660	<0.0001
mitochondrial ribosomal protein S31	Mrps31	1.659	<0.0001
tubulin, beta 2B class IIB	Tubb2b	1.659	<0.0001
pescadillo homolog 1, containing BRCT domain (zebrafish)	Pes1	1.659	<0.0001
mannose phosphate isomerase	Mpi	1.658	<0.0001
chaperonin containing Tcp1, subunit 5 (epsilon)	Cct5	1.658	<0.0001
family with sequence similarity 58, member B	Fam58b	1.658	<0.0001
syntaxin 17	Stx17	1.658	<0.0001
pyrroline-5-carboxylate reductase family, member 2	Pycr2	1.658	<0.0001
taspase, threonine aspartase 1	Tasp1	1.657	<0.0001
adenylate kinase 4	Ak4	1.657	0.0008
inositol hexaphosphate kinase 1	Ip6k1	1.657	<0.0001
mindbomb homolog 2 (Drosophila)	Mib2	1.656	<0.0001
2,4-dienoyl CoA reductase 1, mitochondrial	Decr1	1.656	<0.0001
family with sequence similarity 174, member A	Fam174a	1.654	<0.0001
ornithine decarboxylase antizyme 2	Oaz2	1.654	<0.0001
zinc finger, DHHC domain containing 16	Zdhhc16	1.654	<0.0001
zinc finger, CCHC domain containing 17	Zcchc17	1.653	<0.0001
family with sequence similarity 214, member A	Fam214a	1.653	<0.0001
DEAH (Asp-Glu-Ala-His) box polypeptide 9	Dhx9	1.653	<0.0001
DEAD (Asp-Glu-Ala-Asp) box polypeptide 5	Ddx5	1.652	<0.0001
acyl-CoA synthetase long-chain family member 5	Acsl5	1.652	<0.0001
mitogen-activated protein kinase kinase 5	Map2k5	1.651	<0.0001
D-tyrosyl-tRNA deacylase 1	Dtd1	1.651	<0.0001
vacuolar protein sorting 45 (yeast)	Vps45	1.650	<0.0001
CDC16 cell division cycle 16	Cdc16	1.650	<0.0001
prolyl 4-hydroxylase, beta polypeptide	P4hb	1.650	<0.0001
UTP6, small subunit (SSU) processome component, homolog (yeast)	Utp6	1.650	<0.0001

family with sequence similarity 162, member A	Fam162a	1.649	<0.0001
FAST kinase domains 2	Fastkd2	1.649	<0.0001
katanin p80 (WD40-containing) subunit B 1	Katnb1	1.648	<0.0001
M-phase phosphoprotein 10 (U3 small nucleolar ribonucleoprotein)	Mphosph10	1.648	<0.0001
RAB39B, member RAS oncogene family	Rab39b	1.648	<0.0001
FK506 binding protein 5	Fkbp5	1.648	<0.0001
atlastin GTPase 2	Atl2	1.647	<0.0001
exosome component 6	Exosc6	1.647	<0.0001
syntaxin 4A (placental)	Stx4a	1.647	<0.0001
N(alpha)-acetyltransferase 30, NatC catalytic subunit	Naa30	1.646	<0.0001
mannosyl-oligosaccharide glucosidase	Mogs	1.645	<0.0001
protein phosphatase 5, catalytic subunit	Ppp5c	1.645	<0.0001
coronin, actin binding protein 1B	Coro1b	1.644	<0.0001
methyltransferase like 6	Mettl6	1.644	<0.0001
anterior pharynx defective 1c homolog (C. elegans)	Aph1c	1.643	<0.0001
basic helix-loop-helix domain containing, class B9	Bhlhb9	1.643	<0.0001
LanC (bacterial lantibiotic synthetase component C)-like 2	Lancl2	1.643	<0.0001
mitochondrial ribosomal protein L46	Mrpl46	1.642	<0.0001
golgi autoantigen, golgin subfamily a, 3	Golga3	1.642	<0.0001
HIG1 domain family, member 1A	Higd1a	1.642	<0.0001
UDP-Gal:betaGlcNAc beta 1,3-galactosyltransferase, polypeptide 2	B3galt2	1.642	<0.0001
VPS33B interacting protein, apical-basolateral polarity regulator, spe-39 homolog	Vipas39	1.641	<0.0001
AHA1, activator of heat shock protein ATPase 1	Ahsa1	1.641	<0.0001
polymerase (DNA-directed), delta 4	Pold4	1.641	<0.0001
RNA 2,3-cyclic phosphate and 5-OH ligase	Rtcb	1.640	<0.0001
rhomboid, veinlet-like 1 (Drosophila)	Rhbdl1	1.640	<0.0001
cytochrome C oxidase assembly factor 3	Coa3	1.639	<0.0001
karyopherin (importin) beta 1	Kpnb1	1.638	<0.0001
N-terminal Asn amidase	Ntan1	1.638	<0.0001
PNMA-like 1	Pnmal1	1.633	<0.0001
spermatogenesis associated 2	Spata2	1.633	<0.0001
transmembrane protein 168	Tmem168	1.632	<0.0001
SWI5 dependent recombination repair 1	Sfr1	1.632	<0.0001
PRP19/PSO4 pre-mRNA processing factor 19 homolog (S. cerevisiae)	Prpf19	1.631	<0.0001

Bardet-Biedl syndrome 5 (human)	Bbs5	1.631	<0.0001
pleckstrin homology domain containing, family A (phosphoinositide binding specific) member 1	Plekha1	1.630	<0.0001
vacuolar protein sorting 28 (yeast)	Vps28	1.628	<0.0001
Max dimerization protein 4	Mxd4	1.628	<0.0001
ring finger protein 139	Rnf139	1.628	<0.0001
basic transcription factor 3	Btf3	1.627	0.001
actin related protein 2/3 complex, subunit 4	Arpc4	1.626	<0.0001
OMA1 homolog, zinc metallopeptidase (S. cerevisiae)	Oma1	1.626	<0.0001
small EDRK-rich factor 2	Serf2	1.626	<0.0001
pyruvate dehydrogenase kinase, isoenzyme 1	Pdk1	1.625	<0.0001
CAAX box 1C	Cxx1c	1.625	<0.0001
ATPase, H+ transporting, lysosomal V1 subunit E1	Atp6v1e1	1.625	<0.0001
SET domain containing 3	Setd3	1.625	<0.0001
tectonic family member 2	Tctn2	1.625	<0.0001
proteasome (prosome, macropain) 26S subunit, non-ATPase, 7	Psmd7	1.624	<0.0001
centrosomal protein 83, opposite strand	Cep83os	1.624	<0.0001
protein phosphatase 1A, magnesium dependent, alpha isoform	Ppm1a	1.624	<0.0001
tectonin beta-propeller repeat containing 1	Tecpr1	1.624	<0.0001
signal peptidase complex subunit 2 homolog (S. cerevisiae)	Spcs2	1.624	<0.0001
RAB27B, member RAS oncogene family	Rab27b	1.624	0.0002
endo/exonuclease (5-3), endonuclease G-like	Exog	1.623	<0.0001
phosphatidylinositol-4-phosphate 5-kinase, type 1 alpha	Pip5k1a	1.623	<0.0001
cathepsin D	Ctsd	1.622	<0.0001
zinc finger protein 763	Zfp763	1.622	<0.0001
heat shock 105kDa/110kDa protein 1	Hsph1	1.621	<0.0001
transmembrane protein 126A	Tmem126a	1.619	<0.0001
resistance to inhibitors of cholinesterase 3 homolog (C. elegans)	Ric3	1.618	<0.0001
myotubularin related protein 1	Mtmr1	1.618	<0.0001
tripartite motif-containing 27	Trim27	1.618	<0.0001
myotrophin	Mtpn	1.617	<0.0001
meteorin, glial cell differentiation regulator	Metrn	1.617	<0.0001
eukaryotic translation initiation factor 3, subunit D	Eif3d	1.616	<0.0001
prostaglandin E synthase 3 (cytosolic)	Ptges3	1.616	<0.0001

HECT domain containing 3	Hectd3	1.615	<0.0001
ATP-binding cassette, sub-family B (MDR/TAP), member 6	Abcb6	1.615	<0.0001
ubiquitin family domain containing 1	Ubfd1	1.615	<0.0001
praja ring finger 1, E3 ubiquitin protein ligase	Pja1	1.614	<0.0001
ubiquitin associated domain containing 1	Ubac1	1.614	<0.0001
branched chain ketoacid dehydrogenase E1, beta polypeptide	Bckdhb	1.614	<0.0001
isovaleryl coenzyme A dehydrogenase	Ivd	1.613	<0.0001
F-box protein 31	Fbxo31	1.613	<0.0001
WD repeat domain 20	Wdr20	1.612	<0.0001
H1 histone family, member 0	H1f0	1.612	<0.0001
family with sequence similarity 131, member B	Fam131b	1.611	<0.0001
WD repeat and SOCS box-containing 1	Wsb1	1.611	0.0013
CD82 antigen	Cd82	1.608	<0.0001
junction adhesion molecule 3	Jam3	1.607	<0.0001
coiled-coil-helix-coiled-coil-helix domain containing 10	Chchd10	1.607	<0.0001
SET and MYND domain containing 2	Smyd2	1.606	<0.0001
lysine (K)-specific demethylase 5B	Kdm5b	1.606	<0.0001
WD repeat domain 45B	Wdr45b	1.605	<0.0001
nucleoporin 133	Nup133	1.604	<0.0001
dual specificity phosphatase 12	Dusp12	1.604	<0.0001
ARP3 actin-related protein 3B	Actr3b	1.604	<0.0001
methionine adenosyltransferase II, beta	Mat2b	1.602	<0.0001
mitochondrial ribosomal protein L28	Mrpl28	1.602	<0.0001
F-box protein 6	Fbxo6	1.602	<0.0001
stromal antigen 1	Stag1	1.602	<0.0001
follistatin-like 1	Fstl1	1.601	0.0003
UDP-N-acetylglucosamine pyrophosphorylase 1	Uap1	1.601	<0.0001
NIPA-like domain containing 3	Nipal3	1.600	<0.0001
golgi SNAP receptor complex member 1	Gosr1	1.600	<0.0001
zinc finger protein 810	Zfp810	1.600	<0.0001
protein (peptidyl-prolyl cis/trans isomerase) NIMA-interacting 1	Pin1	1.599	<0.0001
heat shock protein 14	Hspa14	1.599	<0.0001
heat shock protein 70 family, member 13	Hspa13	1.598	<0.0001
COBW domain containing 1	Cbwd1	1.598	<0.0001

peroxiredoxin 1	Prdx1	1.597	0.0004
calcyon neuron-specific vesicular protein	Caly	1.597	<0.0001
UDP-N-acetyl-alpha-D-galactosamine:polypeptide N-acetylgalactosaminyltransferase 11	Galnt11	1.597	<0.0001
origin recognition complex, subunit 6	Orc6	1.597	<0.0001
RNA binding motif protein 17	Rbm17	1.596	<0.0001
MAP/microtubule affinity regulating kinase 3	Mark3	1.596	<0.0001
RAS protein activator like 1 (GAP1 like)	Rasal1	1.595	<0.0001
maturin, neural progenitor differentiation regulator homolog (Xenopus)	Mturn	1.595	<0.0001
carbonyl reductase 3	Cbr3	1.594	0.0019
dehydrogenase/reductase (SDR family) member 7	Dhrs7	1.593	<0.0001
SCY1-like 3 (S. cerevisiae)	Scyl3	1.593	<0.0001
serine/arginine-rich protein specific kinase 2	Srpk2	1.592	<0.0001
methylenetetrahydrofolate dehydrogenase (NADP+ dependent), methenyltetrahydrofolate cyclohydrolase, formyltetrahydrofolate synthase	Mthfd1	1.592	<0.0001
ring finger protein 34	Rnf34	1.591	<0.0001
ganglioside-induced differentiation-associated-protein 1	Gdap1	1.591	<0.0001
tetraspanin 3	Tspan3	1.591	<0.0001
phosphofructokinase, platelet	Pfkp	1.590	<0.0001
Bmi1 polycomb ring finger oncogene	Bmi1	1.590	<0.0001
histone deacetylase 11	Hdac11	1.589	<0.0001
gap junction protein, beta 1	Gjb1	1.589	0.0012
dihydrolipoamide dehydrogenase	Dld	1.588	<0.0001
cysteinyl-tRNA synthetase	Cars	1.587	<0.0001
acyl-Coenzyme A binding domain containing 4	Acbd4	1.587	<0.0001
X-ray repair complementing defective repair in Chinese hamster cells 5	Xrcc5	1.586	<0.0001
programmed cell death 6	Pdcd6	1.586	<0.0001
transducin (beta)-like 1X-linked receptor 1	Tbl1xr1	1.585	<0.0001
regulatory factor X-associated protein	Rfxap	1.585	<0.0001
amyloid beta precursor protein (cytoplasmic tail) binding protein 2	Appbp2	1.584	<0.0001
dual specificity phosphatase 6	Dusp6	1.584	0.0008
ankyrin repeat domain 6	Ankrd6	1.584	<0.0001
mitogen-activated protein kinase 10	Mapk10	1.583	<0.0001
FtsJ homolog 1 (E. coli)	Ftsj1	1.582	<0.0001
small ubiquitin-like modifier 3	Sumo3	1.582	<0.0001

breast carcinoma amplified sequence 2	Bcas2	1.581	<0.0001
X-ray repair complementing defective repair in Chinese hamster cells 4	Xrcc4	1.581	<0.0001
eukaryotic translation initiation factor 2 alpha kinase 3	Eif2ak3	1.580	<0.0001
syntaxin 16	Stx16	1.579	<0.0001
dihydrolipoamide S-acetyltransferase (E2 component of pyruvate dehydrogenase complex)	Dlat	1.579	<0.0001
zinc finger protein 317	Zfp317	1.578	<0.0001
asparaginyl-tRNA synthetase	Nars	1.577	<0.0001
X-linked inhibitor of apoptosis	Xiap	1.577	<0.0001
family with sequence similarity 20, member B	Fam20b	1.577	<0.0001
TATA box binding protein-like 1	Tbpl1	1.577	<0.0001
DiGeorge syndrome critical region gene 6	Dgcr6	1.577	<0.0001
Rho GTPase activating protein 12	Arhgap12	1.577	<0.0001
ATPase family, AAA domain containing 1	Atad1	1.577	<0.0001
sorting nexin 10	Snx10	1.576	<0.0001
AKT interacting protein	Aktip	1.575	<0.0001
glutaryl-Coenzyme A dehydrogenase	Gcdh	1.574	<0.0001
lysophospholipase-like 1	Lyplal1	1.574	<0.0001
transient receptor potential cation channel, subfamily C, member 4 associated protein	Trpc4ap	1.573	<0.0001
NADH dehydrogenase (ubiquinone) 1 beta subcomplex, 5	Ndufb5	1.573	<0.0001
nuclear distribution gene C homolog (Aspergillus)	Nudc	1.573	<0.0001
zinc finger protein 180	Zfp180	1.572	<0.0001
growth hormone inducible transmembrane protein	Ghitm	1.572	<0.0001
chemokine (C-X3-C motif) receptor 1	Cx3cr1	1.572	<0.0001
HIV-1 tat interactive protein 2, homolog (human)	Htatip2	1.571	<0.0001
NOP14 nucleolar protein	Nop14	1.570	<0.0001
G patch domain containing 8	Gpatch8	1.570	<0.0001
DCN1, defective in cullin neddylation 1, domain containing 5 (S. cerevisiae)	Dcun1d5	1.570	<0.0001
sirtuin 3	Sirt3	1.569	<0.0001
golgi phosphoprotein 3	Golph3	1.569	<0.0001
dynein light chain Tctex-type 3	Dynlt3	1.568	<0.0001
Tp53rk binding protein	Tprkb	1.568	<0.0001
transmembrane protein 70	Tmem70	1.568	<0.0001
ER membrane protein complex subunit 3	Emc3	1.568	<0.0001
cortactin	Cttn	1.567	<0.0001

mitochondrial ribosomal protein S18B	Mrps18b	1.566	<0.0001
surfeit gene 1	Surf1	1.566	<0.0001
RAS-like, family 10, member A	Rasl10a	1.565	<0.0001
membrane-associated ring finger (C3HC4) 1	March1	1.565	<0.0001
CCAAT/enhancer binding protein zeta	Cebpz	1.565	<0.0001
syntaxin 18	Stx18	1.564	<0.0001
propionyl-Coenzyme A carboxylase, alpha polypeptide	Pcca	1.564	<0.0001
guanylate cyclase 1, soluble, beta 3	Gucy1b3	1.564	<0.0001
elaC homolog 1 (E. coli)	Elac1	1.563	<0.0001
peroxisomal membrane protein 2	Pxmp2	1.563	<0.0001
jumping translocation breakpoint	Jtb	1.563	<0.0001
armadillo repeat containing 6	Armc6	1.561	<0.0001
isoamyl acetate-hydrolyzing esterase 1 homolog (S. cerevisiae)	Iah1	1.561	<0.0001
phosphatidylserine decarboxylase	Pisd	1.561	<0.0001
solute carrier family 4, sodium bicarbonate cotransporter, member 5	Slc4a5	1.561	<0.0001
sorting nexin 2	Snx2	1.561	<0.0001
SLIT and NTRK-like family, member 4	Slitrk4	1.561	0.0003
thymocyte nuclear protein 1	Thyn1	1.560	<0.0001
casein kinase 1, alpha 1	Csnk1a1	1.559	<0.0001
F-box and leucine-rich repeat protein 3	Fbxl3	1.559	<0.0001
protein kinase C, zeta	Prkcz	1.559	<0.0001
phosphatidylinositol 3 kinase, regulatory subunit, polypeptide 3 (p55)	Pik3r3	1.559	<0.0001
calcium response factor	Carf	1.558	<0.0001
budding uninhibited by benzimidazoles 3 homolog (S. cerevisiae)	Bub3	1.558	<0.0001
cyclin-dependent kinase 5	Cdk5	1.558	<0.0001
nrde-2 necessary for RNA interference, domain containing	Nrde2	1.558	<0.0001
mohawk homeobox	Mkx	1.558	<0.0001
vacuolar protein sorting 18 (yeast)	Vps18	1.557	<0.0001
SYS1 Golgi-localized integral membrane protein homolog (S. cerevisiae)	Sys1	1.557	<0.0001
ARP8 actin-related protein 8	Actr8	1.557	<0.0001
histocompatibility 2, T region locus 22	H2-T22	1.557	<0.0001
UFM1-specific peptidase 2	Ufsp2	1.556	<0.0001
polymerase (RNA) III (DNA directed) polypeptide K	Polr3k	1.555	<0.0001
receptor accessory protein 1	Reep1	1.555	<0.0001

fem-1 homolog c (C.elegans)	Fem1c	1.555	<0.0001
lipoma HMGIC fusion partner-like 2	Lhfpl2	1.555	<0.0001
yippee-like 4 (Drosophila)	Ypel4	1.555	<0.0001
tRNA splicing endonuclease 2 homolog (S. cerevisiae)	Tsen2	1.555	<0.0001
PQ loop repeat containing 1	Pqlc1	1.555	<0.0001
phosphoinositide-3-kinase, class 3	Pik3c3	1.554	<0.0001
anaphase promoting complex subunit 4	Anapc4	1.554	<0.0001
zinc finger prtoein 943	Zfp943	1.554	0.004
U7 snRNP-specific Sm-like protein LSM10	Lsm10	1.554	<0.0001
LIM and SH3 protein 1	Lasp1	1.554	<0.0001
fibroblast growth factor (acidic) intracellular binding protein	Fibp	1.553	<0.0001
solute carrier family 35, member A5	Slc35a5	1.553	<0.0001
RUN domain containing 3B	Rundc3b	1.553	<0.0001
hypoxia-inducible factor 1, alpha subunit inhibitor	Hif1an	1.553	<0.0001
wntless homolog (Drosophila)	Wls	1.553	<0.0001
WDYHV motif containing 1	Wdyhv1	1.552	<0.0001
feminization 1 homolog b (C. elegans)	Fem1b	1.552	<0.0001
armadillo repeat containing, X-linked 1	Armcx1	1.551	<0.0001
phosphatidylinositol binding clathrin assembly protein	Picalm	1.551	<0.0001
transcription factor A, mitochondrial	Tfam	1.551	<0.0001
SUMO/sentrin specific peptidase 3	Senp3	1.551	<0.0001
RB1-inducible coiled-coil 1	Rb1cc1	1.550	0.0002
anaphase promoting complex subunit 11	Anapc11	1.550	<0.0001
UDP-N-acetyl-alpha-D-galactosamine:polypeptide N-acetylgalactosaminyltransferase 16	Galnt16	1.550	<0.0001
RAD1 checkpoint DNA exonuclease	Rad1	1.550	<0.0001
zinc finger protein 941	Zfp941	1.550	<0.0001
centrin 3	Cetn3	1.550	<0.0001
WW domain binding protein 4	Wbp4	1.550	<0.0001
fibrillarin-like 1	Fbll1	1.550	<0.0001
phytanoyl-CoA hydroxylase	Phyh	1.550	<0.0001
sorting nexin 11	Snx11	1.550	<0.0001
SWI/SNF related, matrix associated, actin dependent regulator of chromatin, subfamily d, member 3	Smarcd3	1.549	<0.0001
NADH dehydrogenase (ubiquinone) complex I, assembly factor 5	Ndufaf5	1.549	<0.0001

zinc finger protein 830	Zfp830	1.549	<0.0001
phospholipid phosphatase 1	Plpp1	1.549	<0.0001
WW domain binding protein 1 like	Wbp1l	1.548	<0.0001
exosome component 7	Exosc7	1.548	<0.0001
spermatogenesis associated, serine-rich 2	Spats2	1.548	<0.0001
PTEN induced putative kinase 1	Pink1	1.547	<0.0001
regulator of chromosome condensation 2	Rcc2	1.546	<0.0001
docking protein 4	Dok4	1.546	<0.0001
RAB5A, member RAS oncogene family	Rab5a	1.546	<0.0001
glycerophosphodiester phosphodiesterase 1	Gde1	1.546	<0.0001
centromere protein B	Cenpb	1.546	<0.0001
mitochondrial ribosomal protein S18A	Mrps18a	1.546	<0.0001
mediator complex subunit 17	Med17	1.546	<0.0001
WD repeat domain 24	Wdr24	1.545	<0.0001
ring finger and SPRY domain containing 1	Rspry1	1.544	<0.0001
glutathione peroxidase 1	Gpx1	1.544	<0.0001
clusterin	Clu	1.544	<0.0001
aminoadipate-semialdehyde dehydrogenase-phosphopantetheinyl transferase	Aasdhppt	1.542	<0.0001
armadillo repeat containing 8	Armc8	1.542	<0.0001
amyloid beta (A4) precursor protein-binding, family B, member 3	Apbb3	1.542	<0.0001
polyadenylate binding protein-interacting protein 1	Paip1	1.541	<0.0001
acyl-Coenzyme A dehydrogenase, medium chain	Acadm	1.541	<0.0001
chloride channel, voltage-sensitive 3	Clcn3	1.541	<0.0001
protein phosphatase 2, regulatory subunit B, beta	Ppp2r2b	1.540	<0.0001
UDP-GlcNAc:betaGal beta-1,3-N-acetylglucosaminyltransferase 2	B3gnt2	1.540	0.0002
adaptor protein complex AP-1, sigma 1	Ap1s1	1.540	<0.0001
D-dopachrome tautomerase	Ddt	1.539	<0.0001
3-hydroxybutyrate dehydrogenase, type 1	Bdh1	1.539	<0.0001
mediator complex subunit 18	Med18	1.539	<0.0001
EMG1 nucleolar protein homolog (S. cerevisiae)	Emg1	1.539	<0.0001
adenomatosis polyposis coli down-regulated 1	Apcdd1	1.538	<0.0001
poly(rC) binding protein 4	Pcbp4	1.538	<0.0001
ubiquitin-conjugating enzyme E2Q (putative) 2	Ube2q2	1.538	<0.0001
solute carrier family 7 (cationic amino acid transporter, y+ system), member 4	Slc7a4	1.538	<0.0001

polymerase (RNA) III (DNA directed) polypeptide C	Polr3c	1.538	<0.0001
arylsulfatase G	Arsg	1.537	0.0098
exostoses (multiple) 2	Ext2	1.537	<0.0001
glutathione S-transferase, mu 4	Gstm4	1.537	<0.0001
zinc finger protein 35	Zfp35	1.537	<0.0001
POU domain, class 3, transcription factor 3 adjacent noncoding transcript 1	Pantr1	1.537	<0.0001
glucosidase, beta, acid	Gba	1.536	<0.0001
aspartyl-tRNA synthetase 2 (mitochondrial)	Dars2	1.536	<0.0001
histidine triad nucleotide binding protein 3	Hint3	1.536	0.0001
sorting nexin 17	Snx17	1.536	<0.0001
S100 calcium binding protein A13	S100a13	1.536	<0.0001
Der1-like domain family, member 1	Derl1	1.535	0.0001
zinc finger SWIM-type containing 1	Zswim1	1.535	<0.0001
WD repeat domain 53	Wdr53	1.535	<0.0001
small nuclear ribonucleoprotein 25 (U11/U12)	Snrnp25	1.535	<0.0001
leucine zipper, putative tumor suppressor family member 3	Lzts3	1.534	<0.0001
tetraspanin 2	Tspan2	1.534	<0.0001
golgi coiled coil 1	Gcc1	1.534	<0.0001
proteasome (prosome, macropain) 26S subunit, non-ATPase, 13	Psmd13	1.534	<0.0001
ribosomal protein S6 kinase-like 1	Rps6kl1	1.534	<0.0001
polymerase (RNA) II (DNA directed) polypeptide I	Polr2i	1.533	<0.0001
transmembrane protein 241	Tmem241	1.533	<0.0001
general transcription factor IIF, polypeptide 1	Gtf2f1	1.533	<0.0001
2-4-dienoyl-Coenzyme A reductase 2, peroxisomal	Decr2	1.533	<0.0001
phospholipase A2, group XV	Pla2g15	1.533	<0.0001
transcriptional adaptor 1	Tada1	1.533	<0.0001
5-aminoimidazole-4-carboxamide ribonucleotide formyltransferase/IMP cyclohydrolase	Atic	1.532	<0.0001
aminolevulinic acid synthase 1	Alas1	1.532	<0.0001
RUN and FYVE domain containing 3	Rufy3	1.532	<0.0001
topoisomerase (DNA) II beta	Top2b	1.532	<0.0001
cytochrome b5 reductase 1	Cyb5r1	1.532	<0.0001
flightless I homolog (Drosophila)	Flii	1.532	<0.0001
L antigen family, member 3	Lage3	1.531	0.0002
ubiquitin-like modifier activating enzyme 5	Uba5	1.531	<0.0001

guanine nucleotide binding protein (G protein), beta 4	Gnb4	1.530	0.0005
Rab9 effector protein with kelch motifs	Rabepk	1.530	<0.0001
mitochondrial ribosomal protein L45	Mrpl45	1.530	<0.0001
JNK1/MAPK8-associated membrane protein	Jkamp	1.530	<0.0001
tetraspanin 4	Tspan4	1.530	<0.0001
glutamate-rich WD repeat containing 1	Grwd1	1.529	<0.0001
proteasome (prosome, macropain) 26S subunit, ATPase, 6	Psmc6	1.529	<0.0001
exocyst complex component 2	Exoc2	1.529	<0.0001
inhibitor of growth family, member 2	Ing2	1.529	<0.0001
ribosomal protein L30	Rpl30	1.528	0.0003
F-box protein 7	Fbxo7	1.528	<0.0001
serine (or cysteine) peptidase inhibitor, clade B, member 6a	Serpinb6a	1.528	<0.0001
twist basic helix-loop-helix transcription factor 1 neighbor	Twistnb	1.528	<0.0001
anaphase promoting complex subunit 2	Anapc2	1.528	<0.0001
myocardial zonula adherens protein	Myzap	1.528	<0.0001
histone cluster 1, H1b	Hist1h1b	1.528	0.0001
mitochondrial ribosomal protein L1	Mrpl1	1.528	<0.0001
huntingtin interacting protein 1 related	Hip1r	1.528	<0.0001
transmembrane protein 192	Tmem192	1.528	<0.0001
queuine tRNA-ribosyltransferase domain containing 1	Qtrtd1	1.527	<0.0001
A kinase (PRKA) anchor protein 7	Akap7	1.526	<0.0001
translocase of inner mitochondrial membrane 17b	Timm17b	1.526	<0.0001
N-acetylneuraminic acid synthase (sialic acid synthase)	Nans	1.525	<0.0001
solute carrier family 9 (sodium/hydrogen exchanger), member 6	Slc9a6	1.525	<0.0001
lactate dehydrogenase B	Ldhb	1.525	<0.0001
aldo-keto reductase family 1, member A1 (aldehyde reductase)	Akr1a1	1.524	<0.0001
von Willebrand factor A domain containing 5A	Vwa5a	1.524	<0.0001
CKLF-like MARVEL transmembrane domain containing 6	Cmtm6	1.524	<0.0001
immunity-related GTPase family, Q	Irgq	1.524	<0.0001
protein phosphatase 1, regulatory (inhibitor) subunit 3C	Ppp1r3c	1.524	<0.0001
mediator complex subunit 7	Med7	1.522	0.0002
thioredoxin interacting protein	Txnip	1.522	<0.0001
GDP-mannose 4, 6-dehydratase	Gmds	1.521	<0.0001
RAN binding protein 6	Ranbp6	1.521	<0.0001

mediator complex subunit 22	Med22	1.520	<0.0001
alkB, alkylation repair homolog 8 (E. coli)	Alkbh8	1.519	<0.0001
mediator complex subunit 4	Med4	1.518	<0.0001
hnRNP-associated with lethal yellow	Raly	1.518	<0.0001
solute carrier family 4 (anion exchanger), member 8	Slc4a8	1.518	<0.0001
ribosomal protein S13	Rps13	1.518	<0.0001
succinate-Coenzyme A ligase, ADP-forming, beta subunit	Sucla2	1.518	<0.0001
coactosin-like 1 (Dictyostelium)	Cotl1	1.516	<0.0001
zinc finger protein 148	Zfp148	1.516	<0.0001
kelch-like ECH-associated protein 1	Keap1	1.515	<0.0001
ATP synthase, H+ transporting, mitochondrial F1 complex, gamma polypeptide 1	Atp5c1	1.514	<0.0001
chloride channel, nucleotide-sensitive, 1A	Clns1a	1.513	<0.0001
ribophorin II	Rpn2	1.513	<0.0001
B cell translocation gene 1, anti-proliferative	Btg1	1.512	<0.0001
ring finger protein 208	Rnf208	1.512	<0.0001
ribosomal protein S19 binding protein 1	Rps19bp1	1.512	<0.0001
DEAD (Asp-Glu-Ala-Asp) box polypeptide 28	Ddx28	1.512	<0.0001
adducin 1 (alpha)	Add1	1.511	<0.0001
potassium channel tetramerisation domain containing 20	Kctd20	1.511	<0.0001
transmembrane protein 55A	Tmem55a	1.511	<0.0001
TBC1 domain family, member 22a	Tbc1d22a	1.511	<0.0001
transmembrane protein 59	Tmem59	1.510	<0.0001
solute carrier family 45, member 1	Slc45a1	1.510	<0.0001
uridine-cytidine kinase 1	Uck1	1.509	<0.0001
proteasome (prosome, macropain) subunit, beta type 10	Psmb10	1.509	<0.0001
integral membrane protein 2B	Itm2b	1.509	<0.0001
ubiquitin specific peptidase like 1	Uspl1	1.508	<0.0001
solute carrier family 5 (inositol transporters), member 3	Slc5a3	1.508	0.0001
UDP-N-acetyl-alpha-D-galactosamine:polypeptide N-acetylgalactosaminyltransferase 1	Galnt1	1.508	<0.0001
metallophosphoesterase 1	Mppe1	1.508	<0.0001
BTB (POZ) domain containing 6	Btbd6	1.508	<0.0001
lysosomal-associated membrane protein family, member 5	Lamp5	1.507	<0.0001
src homology 2 domain-containing transforming protein C1	Shc1	1.506	<0.0001
adenylate kinase 2	Ak2	1.506	<0.0001

general transcription factor II A, 1	Gtf2a1	1.505	<0.0001
exosome component 4	Exosc4	1.505	<0.0001
Ctr9, Paf1/RNA polymerase II complex component, homolog (S. cerevisiae)	Ctr9	1.504	<0.0001
kinesin family member 1C	Kif1c	1.504	<0.0001
glycerophosphocholine phosphodiesterase GDE1 homolog (S. cerevisiae)	Gpcpd1	1.504	<0.0001
calcium channel flower domain containing 1	Cacfd1	1.503	<0.0001
POU domain, class 3, transcription factor 2	Pou3f2	1.503	<0.0001
unc-119 homolog B (C. elegans)	Unc119b	1.503	<0.0001
methyltransferase like 18	Mettl18	1.503	<0.0001
MYB binding protein (P160) 1a	Mybbp1a	1.503	<0.0001
general transcription factor II H, polypeptide 1	Gtf2h1	1.502	<0.0001
protein phosphatase 1, regulatory (inhibitor) subunit 1A	Ppp1r1a	1.502	0.0002
snurportin 1	Snupn	1.502	<0.0001
nitrogen permease regulator-like 3	Nprl3	1.502	<0.0001
smg-8 homolog, nonsense mediated mRNA decay factor (C. elegans)	Smg8	1.501	<0.0001
Eph receptor A5	Epha5	1.501	<0.0001
histone deacetylase 2	Hdac2	1.501	<0.0001
ribosomal protein S15A	Rps15a	1.500	<0.0001
myeloid/lymphoid or mixed-lineage leukemia (trithorax homolog, Drosophila)	Mllt11	1.500	<0.0001
agrin	Agrn	0.499	<0.0001
protein tyrosine phosphatase, receptor type, J	Ptprj	0.499	<0.0001
mitochondrial ribosomal protein L34	Mrpl34	0.499	<0.0001
RAS-related protein-1a	Rap1a	0.498	<0.0001
heparan sulfate (glucosamine) 3-O-sulfotransferase 1	Hs3st1	0.497	<0.0001
ring finger protein 24	Rnf24	0.495	<0.0001
leucine rich repeat transmembrane neuronal 3	Lrrtm3	0.493	<0.0001
ubiquitin-associated protein 2	Ubap2	0.492	<0.0001
sphingomyelin phosphodiesterase 3, neutral	Smpd3	0.491	<0.0001
dicer 1, ribonuclease type III	Dicer1	0.491	<0.0001
tweety homolog 3 (Drosophila)	Ttyh3	0.490	<0.0001
four and a half LIM domains 1	Fhl1	0.489	<0.0001
nuclear receptor co-repressor 1	Ncor1	0.489	<0.0001
proline rich 36	Prr36	0.488	<0.0001
adhesion G protein-coupled receptor G1	Adgrg1	0.488	<0.0001

delta-like 1 homolog (Drosophila)	Dlk1	0.487	<0.0001
leucine rich adaptor protein 1-like	Lurap1l	0.487	<0.0001
ubiquilin 2	Ubqln2	0.486	<0.0001
regulator of G-protein signalling 10	Rgs10	0.486	<0.0001
staufen (RNA binding protein) homolog 1 (Drosophila)	Stau1	0.486	<0.0001
patched homolog 1	Ptch1	0.486	<0.0001
DNL-type zinc finger	Dnlz	0.485	<0.0001
zinc finger protein 523	Zfp523	0.485	<0.0001
solute carrier family 27 (fatty acid transporter), member 4	Slc27a4	0.485	<0.0001
beta-1,3-glucuronyltransferase 2 (glucuronosyltransferase S)	B3gat2	0.484	<0.0001
sprouty-related, EVH1 domain containing 2	Spred2	0.483	<0.0001
F-box and WD-40 domain protein 5	Fbxw5	0.482	<0.0001
tenascin R	Tnr	0.480	<0.0001
GTP binding protein 2	Gtpbp2	0.480	<0.0001
poly (A) polymerase alpha	Papola	0.478	<0.0001
microtubule associated monooxygenase, calponin and LIM domain containing 2	Mical2	0.478	<0.0001
solute carrier family 25, member 37	Slc25a37	0.478	<0.0001
myelin transcription factor 1-like	Myt1l	0.477	<0.0001
zinc finger, MIZ-type containing 1	Zmiz1	0.477	<0.0001
syntaxin 1B	Stx1b	0.476	<0.0001
serine/arginine repetitive matrix 1	Srrm1	0.475	<0.0001
potassium voltage-gated channel, Shal-related family, member 3	Kcnd3	0.475	<0.0001
ring finger protein 40	Rnf40	0.475	<0.0001
guanine nucleotide binding protein (G protein), gamma 4	Gng4	0.475	<0.0001
heparan sulfate 6-O-sulfotransferase 3	Hs6st3	0.475	<0.0001
bromodomain containing 4	Brd4	0.475	<0.0001
vascular endothelial growth factor B	Vegfb	0.474	<0.0001
immunoglobulin superfamily, member 11	Igsf11	0.473	<0.0001
neuron navigator 1	Nav1	0.472	<0.0001
shisa family member 7	Shisa7	0.471	<0.0001
actinin, alpha 1	Actn1	0.471	<0.0001
F-box and leucine-rich repeat protein 20	Fbxl20	0.471	<0.0001
heterogeneous nuclear ribonucleoprotein U-like 2	Hnrnpul2	0.470	<0.0001
carbonic anyhydrase 12	Car12	0.470	<0.0001

androgen receptor	Ar	0.469	<0.0001
NADH dehydrogenase (ubiquinone) Fe-S protein 7	Ndufs7	0.469	<0.0001
ataxin 1	Atxn1	0.467	<0.0001
FXYD domain-containing ion transport regulator 5	Fxyd5	0.465	<0.0001
forkhead box P1	Foxp1	0.465	<0.0001
voltage-dependent anion channel 3	Vdac3	0.464	<0.0001
regulator of G-protein signaling 2	Rgs2	0.464	<0.0001
solute carrier family 20, member 1	Slc20a1	0.463	<0.0001
nuclear transport factor 2	Nutf2	0.463	<0.0001
ankyrin repeat domain 33B	Ankrd33b	0.461	<0.0001
human immunodeficiency virus type I enhancer binding protein 1	Hivep1	0.460	<0.0001
enabled homolog (Drosophila)	Enah	0.460	<0.0001
TGF-beta activated kinase 1/MAP3K7 binding protein 2	Tab2	0.458	<0.0001
solute carrier family 7 (cationic amino acid transporter, y+ system), member 10	Slc7a10	0.457	<0.0001
synuclein, alpha	Snca	0.457	<0.0001
clavesin 1	Clvs1	0.454	<0.0001
centriolar coiled coil protein 110	Ccp110	0.454	<0.0001
ring finger protein 128	Rnf128	0.453	<0.0001
forkhead box K1	Foxk1	0.452	<0.0001
sine oculis-binding protein homolog (Drosophila)	Sobp	0.452	<0.0001
calcium channel, voltage-dependent, gamma subunit 4	Cacng4	0.452	<0.0001
proline and serine rich 1	Proser1	0.451	<0.0001
zinc finger SWIM-type containing 6	Zswim6	0.450	<0.0001
discs, large homolog-associated protein 4 (Drosophila)	Dlgap4	0.449	<0.0001
SH3 domain containing ring finger 2	Sh3rf2	0.449	0.0008
solute carrier family 39 (metal ion transporter), member 13	Slc39a13	0.448	<0.0001
ataxin 7-like 1	Atxn7l1	0.446	<0.0001
human immunodeficiency virus type I enhancer binding protein 2	Hivep2	0.445	<0.0001
acetylcholinesterase	Ache	0.445	0.0003
ribosomal protein S27-like	Rps27l	0.445	<0.0001
pseudopodium-enriched atypical kinase 1	Peak1	0.444	<0.0001
serine/threonine kinase 24	Stk24	0.443	<0.0001
DDB1 and CUL4 associated factor 12	Dcaf12	0.443	<0.0001
calcium binding protein 39	Cab39	0.442	<0.0001

mannosidase, endo-alpha-like	Maneal	0.442	<0.0001
atrophin 1	Atn1	0.441	<0.0001
Rho GDP dissociation inhibitor (GDI) alpha	Arhgdia	0.441	<0.0001
adaptor-related protein complex 3, mu 1 subunit	Ap3m1	0.441	<0.0001
carbohydrate sulfotransferase 11	Chst11	0.441	<0.0001
cellular repressor of E1A-stimulated genes 1	Creg1	0.440	<0.0001
trafficking protein, kinesin binding 1	Trak1	0.439	<0.0001
formin-like 1	Fmnl1	0.439	<0.0001
oligosaccharyltransferase 4 homolog (S. cerevisiae)	Ost4	0.439	<0.0001
CD24a antigen	Cd24a	0.438	<0.0001
fibroblast growth factor receptor substrate 2	Frs2	0.438	<0.0001
serine/threonine kinase 40	Stk40	0.438	<0.0001
H1 histone family, member X	H1fx	0.438	<0.0001
metastasis suppressor 1-like	Mtss1l	0.435	<0.0001
sortilin-related VPS10 domain containing receptor 1	Sorcs1	0.434	<0.0001
dynactin 6	Dctn6	0.433	<0.0001
adenosine A2a receptor	Adora2a	0.433	0.0027
proline arginine-rich end leucine-rich repeat	Prelp	0.433	<0.0001
solute carrier family 38, member 6	Slc38a6	0.431	<0.0001
suppressor of Ty 3	Supt3	0.429	<0.0001
5-nucleotidase, cytosolic II	Nt5c2	0.428	<0.0001
chromobox 7	Cbx7	0.428	<0.0001
ubiquitin specific peptidase 54	Usp54	0.428	<0.0001
Purkinje cell protein 4-like 1	Pcp4l1	0.428	<0.0001
Y box protein 3	Ybx3	0.428	<0.0001
tetraspanin 7	Tspan7	0.427	<0.0001
neural precursor cell expressed, developmentally down-regulated gene 4-like	Nedd4l	0.427	<0.0001
SH3 and cysteine rich domain 2	Stac2	0.426	<0.0001
RAB, member RAS oncogene family-like 6	Rabl6	0.425	<0.0001
glycine amidinotransferase (L-arginine:glycine amidinotransferase)	Gatm	0.424	<0.0001
claudin domain containing 1	Cldnd1	0.423	<0.0001
solute carrier family 9 (sodium/hydrogen exchanger), member 1	Slc9a1	0.423	<0.0001
DNA segment, Chr 8, ERATO Doi 738, expressed	D8Ertd738e	0.423	<0.0001
neurexin III	Nrxn3	0.422	<0.0001

galactose-3-O-sulfotransferase 3	Gal3st3	0.422	<0.0001
ankyrin 2, brain	Ank2	0.418	<0.0001
peroxisome proliferative activated receptor, gamma, coactivator 1 alpha	Ppargc1a	0.415	<0.0001
transmembrane protein 141	Tmem141	0.414	<0.0001
catalase	Cat	0.414	<0.0001
GRAM domain containing 1A	Gramd1a	0.414	<0.0001
zinc finger protein 281	Zfp281	0.414	<0.0001
ubiquinol-cytochrome c reductase complex assembly factor 2	Uqcc2	0.414	<0.0001
SH3-domain binding protein 5 (BTK-associated)	Sh3bp5	0.413	<0.0001
cholinergic receptor, nicotinic, alpha polypeptide 4	Chrna4	0.413	<0.0001
TAF9B RNA polymerase II, TATA box binding protein (TBP)-associated factor	Taf9b	0.411	<0.0001
activity regulated cytoskeletal-associated protein	Arc	0.410	<0.0001
forkhead box J2	Foxj2	0.406	<0.0001
ST8 alpha-N-acetyl-neuraminide alpha-2,8-sialyltransferase 1	St8sia1	0.405	<0.0001
parathymosin	Ptms	0.404	<0.0001
PET100 homolog (S. cerevisiae)	Pet100	0.403	<0.0001
potassium inwardly-rectifying channel, subfamily J, member 10	Kcnj10	0.403	<0.0001
transducin-like enhancer of split 3, homolog of Drosophila E(spl)	Tle3	0.402	<0.0001
Ngfi-A binding protein 2	Nab2	0.402	<0.0001
cortexin 1	Ctxn1	0.400	<0.0001
RAB GTPase activating protein 1	Rabgap1	0.399	<0.0001
phosphodiesterase 10A	Pde10a	0.399	0.001
neuropilin (NRP) and tolloid (TLL)-like 2	Neto2	0.399	<0.0001
metallothionein 2	Mt2	0.397	<0.0001
solute carrier family 36 (proton/amino acid symporter), member 1	Slc36a1	0.397	<0.0001
DEAD/H (Asp-Glu-Ala-Asp/His) box polypeptide 26B	Ddx26b	0.395	<0.0001
long non-protein coding RNA, Trp53 induced transcript	Lncpint	0.394	<0.0001
disabled 1	Dab1	0.391	<0.0001
beta-transducin repeat containing protein	Btrc	0.387	<0.0001
splicing factor, suppressor of white-apricot homolog (Drosophila)	Sfswap	0.386	<0.0001
leucine rich repeat containing 61	Lrrc61	0.386	<0.0001
methylmalonyl CoA epimerase	Mcee	0.386	<0.0001
guanine nucleotide binding protein, alpha O	Gnao1	0.386	<0.0001
deoxyguanosine kinase	Dguok	0.385	<0.0001

pre B cell leukemia homeobox 3	Pbx3	0.385	<0.0001
zinc fingers and homeoboxes 3	Zhx3	0.383	<0.0001
R3H domain containing 2	R3hdm2	0.381	<0.0001
desumoylating isopeptidase 1	Desi1	0.378	<0.0001
uridine-cytidine kinase 2	Uck2	0.378	<0.0001
NADH dehydrogenase (ubiquinone) 1, subcomplex unknown, 2	Ndufc2	0.377	<0.0001
proliferation-associated 2G4	Pa2g4	0.374	<0.0001
OTU domain, ubiquitin aldehyde binding 2	Otub2	0.373	<0.0001
V-set and transmembrane domain containing 2-like	Vstm2l	0.373	<0.0001
ubiquitin-associated protein 2-like	Ubap2l	0.372	<0.0001
transmembrane protein 230	Tmem230	0.371	<0.0001
vesicle amine transport protein 1 homolog (T californica)	Vat1	0.371	<0.0001
3-hydroxyacyl-CoA dehydratase 3	Hacd3	0.370	<0.0001
zinc finger protein 384	Zfp384	0.369	<0.0001
hairy/enhancer-of-split related with YRPW motif 1	Hey1	0.367	<0.0001
low density lipoprotein receptor-related protein 1	Lrp1	0.366	<0.0001
formin binding protein 1	Fnbp1	0.366	<0.0001
Sin3A associated protein	Sap130	0.365	<0.0001
teashirt zinc finger family member 3	Tshz3	0.364	<0.0001
nuclear factor I/X	Nfix	0.363	<0.0001
GATA zinc finger domain containing 2B	Gatad2b	0.363	<0.0001
Wiskott-Aldrich syndrome-like (human)	Wasl	0.357	<0.0001
cold shock domain containing C2, RNA binding	Csdc2	0.357	<0.0001
triple functional domain (PTPRF interacting)	Trio	0.355	<0.0001
S100 calcium binding protein A1	S100a1	0.355	<0.0001
retinoblastoma binding protein 6	Rbbp6	0.353	<0.0001
splicing factor 1	Sf1	0.351	<0.0001
nuclear receptor binding protein 2	Nrbp2	0.350	<0.0001
myeloid/lymphoid or mixed-lineage leukemia (trithorax homolog, Drosophila)	Mllt1	0.350	<0.0001
myosin, light polypeptide 4	Myl4	0.350	<0.0001
zinc finger, DHHC domain containing 18	Zdhhc18	0.348	<0.0001
zinc finger protein 207	Zfp207	0.347	<0.0001
5-hydroxytryptamine (serotonin) receptor 1B	Htr1b	0.342	<0.0001
DNA binding protein with his-thr domain	Dbpht2	0.339	<0.0001

negative elongation factor complex member A, Whsc2	Nelfa	0.338	<0.0001
dishevelled 3, dsh homolog (Drosophila)	Dvl3	0.336	<0.0001
prostate transmembrane protein, androgen induced 1	Pmepa1	0.334	<0.0001
runt-related transcription factor 1	Runx1t1	0.333	<0.0001
pannexin 2	Panx2	0.333	<0.0001
glutamyl-tRNA(Gln) amidotransferase, subunit C	Gatc	0.329	<0.0001
fatty acid 2-hydroxylase	Fa2h	0.328	<0.0001
G-protein coupled receptor 88	Gpr88	0.323	0.0003
gamma-aminobutyric acid (GABA) A receptor, subunit beta 3	Gabrb3	0.321	<0.0001
sirtuin 6	Sirt6	0.320	<0.0001
signal sequence receptor, alpha	Ssr1	0.320	<0.0001
teneurin transmembrane protein 2	Tenm2	0.319	<0.0001
coiled-coil domain containing 53	Ccdc53	0.310	<0.0001
UDP-Gal:betaGlcNAc beta 1,4-galactosyltransferase, polypeptide 6	B4galt6	0.306	<0.0001
retinoid X receptor gamma	Rxrg	0.305	0.0001
CREB regulated transcription coactivator 1	Crtc1	0.304	<0.0001
nuclear factor I/A	Nfia	0.303	<0.0001
beta-1,4-N-acetyl-galactosaminyl transferase 1	B4galnt1	0.298	<0.0001
poly(rC) binding protein 1	Pcbp1	0.293	<0.0001
required for meiotic nuclear division 1 homolog (S. cerevisiae)	Rmnd1	0.288	<0.0001
ciliary neurotrophic factor receptor	Cntfr	0.285	<0.0001
POU domain, class 3, transcription factor 4	Pou3f4	0.285	<0.0001
insulin-like growth factor binding protein 7	Igfbp7	0.282	<0.0001
unc-13 homolog C (C. elegans)	Unc13c	0.280	<0.0001
calcium binding and coiled coil domain 1	Calcoco1	0.266	<0.0001
neuronal PAS domain protein 2	Npas2	0.257	<0.0001
cysteine and histidine-rich domain (CHORD)-containing, zinc-binding protein 1	Chordc1	0.254	<0.0001
MORN repeat containing 2	Morn2	0.250	<0.0001
selenoprotein K	Selk	0.249	<0.0001
adaptor-related protein complex 2, mu 1 subunit	Ap2m1	0.245	<0.0001
transformation related protein 53 inducible nuclear protein 2	Trp53inp2	0.244	<0.0001
early growth response 3	Egr3	0.243	<0.0001
serine/arginine repetitive matrix 2	Srrm2	0.241	<0.0001
SH3-domain GRB2-like endophilin B2	Sh3glb2	0.238	<0.0001

WNK lysine deficient protein kinase 1	Wnk1	0.224	<0.0001
nardilysin, N-arginine dibasic convertase, NRD convertase 1	Nrd1	0.217	<0.0001
ataxin 2-like	Atxn2l	0.213	<0.0001
cysteine-rich hydrophobic domain 2	Chic2	0.204	<0.0001
LSM12 homolog (S. cerevisiae)	Lsm12	0.202	<0.0001
dopamine receptor D2	Drd2	0.196	<0.0001
nuclear receptor subfamily 4, group A, member 2	Nr4a2	0.157	<0.0001
solute carrier family 6 (neurotransmitter transporter, creatine), member 8	Slc6a8	0.153	<0.0001
cholinergic receptor, muscarinic 1, CNS	Chrm1	0.097	<0.0001
fatty acid binding protein 7, brain	Fabp7	0.072	<0.0001

Supplementary Table 2: Biological processes that were significantly enriched or depleted of the genes and non-coding RNA that had altered levels expression in the cortex of Chrm1-/-.

Settings:
Analysis Type: PANTHER Overrepresentation Test (Released 20200728)
Annotation Version and Release Date: PANTHER version 15.0 Released 2020-02-14
Analysed List: Client Text Box Input (Mus musculus)
Reference List: Mus musculus (all genes in database)
Annotation Data Set: PANTHER GO-slim Biological Process
Test Type: FISHER
Correction: FDR

PANTHER GO-Slim Biological Process	Fold Enrichment	P (FDR α 0.01)
signal peptide processing (GO:0006465)	11.40	5.12E-04
proteasome assembly (GO:0043248)	9.31	9.88E-03
establishment of protein localization to endoplasmic reticulum (GO:0072599)	5.21	6.03E-03
protein targeting to ER (GO:0045047)	5.21	6.06E-03
transcription initiation from RNA polymerase II promoter (GO:0006367)	4.89	4.26E-03
DNA-templated transcription, initiation (GO:0006352)	4.74	7.32E-04
response to oxidative stress (GO:0006979)	4.69	5.26E-03
protein targeting to mitochondrion (GO:0006626)	4.60	8.90E-04
establishment of protein localization to mitochondrion (GO:0072655)	4.60	8.96E-04
protein localization to mitochondrion (GO:0070585)	4.60	9.02E-04
electron transport chain (GO:0022900)	4.34	4.31E-03
respiratory electron transport chain (GO:0022904)	4.34	4.34E-03
response to toxic substance (GO:0009636)	4.07	6.27E-03
mitochondrial transport (GO:0006839)	3.61	2.68E-04
vacuole organization (GO:0007033)	3.59	7.43E-04
ATP metabolic process (GO:0046034)	3.49	1.53E-03
cellular respiration (GO:0045333)	3.37	2.06E-03
protein processing (GO:0016485)	3.32	3.69E-03
ribosomal small subunit biogenesis (GO:0042274)	3.32	5.48E-03
energy derivation by oxidation of organic compounds (GO:0015980)	3.21	1.35E-03
generation of precursor metabolites and energy (GO:0006091)	3.16	6.02E-05
purine nucleotide biosynthetic process (GO:0006164)	3.09	8.76E-04
ribonucleotide biosynthetic process (GO:0009260)	3.05	1.51E-03
purine ribonucleotide biosynthetic process (GO:0009152)	3.03	2.28E-03
purine nucleotide metabolic process (GO:0006163)	3.02	9.48E-06
protein maturation (GO:0051604)	3.02	1.12E-03
purine ribonucleotide metabolic process (GO:0009150)	3.01	2.00E-05
ribonucleotide metabolic process (GO:0009259)	2.99	1.56E-05

ribose phosphate biosynthetic process (GO:0046390)	2.98	1.27E-03
purine-containing compound biosynthetic process (GO:0072522)	2.98	1.27E-03
protein targeting (GO:0006605)	2.96	7.28E-05
ribose phosphate metabolic process (GO:0019693)	2.92	1.68E-05
protein folding (GO:0006457)	2.90	2.44E-03
endosomal transport (GO:0016197)	2.88	3.75E-03
purine-containing compound metabolic process (GO:0072521)	2.83	1.94E-05
mitochondrion organization (GO:0007005)	2.81	1.11E-04
nucleoside phosphate biosynthetic process (GO:1901293)	2.81	8.48E-04
establishment of protein localization to organelle (GO:0072594)	2.77	2.96E-05
regulation of protein complex assembly (GO:0043254)	2.77	1.91E-03
nucleotide metabolic process (GO:0009117)	2.72	3.48E-05
nucleotide biosynthetic process (GO:0009165)	2.71	1.76E-03
nucleobase-containing small molecule metabolic process (GO:0055086)	2.64	6.19E-06
nucleoside phosphate metabolic process (GO:0006753)	2.64	4.92E-05
ribonucleoprotein complex assembly (GO:0022618)	2.61	2.59E-03
glycoprotein biosynthetic process (GO:0009101)	2.58	3.84E-03
rRNA metabolic process (GO:0016072)	2.56	1.55E-03
small molecule catabolic process (GO:0044282)	2.53	4.28E-03
peptide metabolic process (GO:0006518)	2.51	6.02E-07
ribonucleoprotein complex subunit organization (GO:0071826)	2.49	3.69E-03
translation (GO:0006412)	2.47	2.22E-05
translational elongation (GO:0006414)	2.47	2.24E-05
protein-containing complex assembly (GO:0065003)	2.45	7.46E-09
cellular protein-containing complex assembly (GO:0034622)	2.45	2.99E-08
peptide biosynthetic process (GO:0043043)	2.44	4.18E-05
carbohydrate derivative biosynthetic process (GO:1901137)	2.43	1.40E-05
glycoprotein metabolic process (GO:0009100)	2.38	6.01E-03
rRNA processing (GO:0006364)	2.38	7.20E-03
cellular amide metabolic process (GO:0043603)	2.37	1.56E-07
amide biosynthetic process (GO:0043604)	2.33	3.60E-05
ncRNA metabolic process (GO:0034660)	2.33	4.15E-05
proteasomal protein catabolic process (GO:0010498)	2.29	3.41E-04
proteasome-mediated ubiquitin-dependent protein catabolic process (GO:0043161)	2.28	5.94E-04
ribonucleoprotein complex biogenesis (GO:0022613)	2.24	1.57E-04
ubiquitin-dependent protein catabolic process (GO:0006511)	2.22	5.55E-05
modification-dependent macromolecule catabolic process (GO:0043632)	2.20	3.91E-05
protein-containing complex subunit organization (GO:0043933)	2.19	9.30E-08
modification-dependent protein catabolic process (GO:0019941)	2.19	5.13E-05
organophosphate biosynthetic process (GO:0090407)	2.18	4.26E-03
organonitrogen compound biosynthetic process (GO:1901566)	2.17	4.59E-09
ncRNA processing (GO:0034470)	2.17	2.35E-03
cofactor metabolic process (GO:0051186)	2.17	4.42E-03
cellular macromolecule catabolic process (GO:0044265)	2.16	1.07E-06

proteolysis involved in cellular protein catabolic process (GO:0051603)	2.16	2.25E-05
cellular protein catabolic process (GO:0044257)	2.15	2.32E-05
protein localization to organelle (GO:0033365)	2.15	5.60E-05
protein catabolic process (GO:0030163)	2.13	1.98E-05
macromolecule catabolic process (GO:0009057)	2.11	8.83E-07
carbohydrate derivative metabolic process (GO:1901135)	2.11	1.11E-05
ribosome biogenesis (GO:0042254)	2.07	8.93E-03
intracellular transport (GO:0046907)	2.02	1.17E-07
intracellular protein transport (GO:0006886)	2.02	5.83E-06
organophosphate metabolic process (GO:0019637)	1.96	9.15E-04
mRNA metabolic process (GO:0016071)	1.95	3.10E-03
establishment of protein localization (GO:0045184)	1.94	4.33E-06
organonitrogen compound catabolic process (GO:1901565)	1.94	3.47E-05
amide transport (GO:0042886)	1.93	5.22E-06
protein transport (GO:0015031)	1.93	7.11E-06
RNA processing (GO:0006396)	1.92	1.26E-04
protein modification by small protein conjugation or removal (GO:0070647)	1.92	3.03E-03
cellular component biogenesis (GO:0044085)	1.91	1.07E-08
peptide transport (GO:0015833)	1.91	1.13E-05
cellular catabolic process (GO:0044248)	1.89	1.64E-07
organic substance catabolic process (GO:1901575)	1.89	5.50E-07
cellular component assembly (GO:0022607)	1.88	5.11E-07
establishment of localization in cell (GO:0051649)	1.86	7.53E-07
cellular protein localization (GO:0034613)	1.85	5.33E-06
cellular macromolecule localization (GO:0070727)	1.85	5.48E-06
proteolysis (GO:0006508)	1.80	6.22E-06
nitrogen compound transport (GO:0071705)	1.78	2.24E-05
small molecule metabolic process (GO:0044281)	1.78	9.28E-05
protein localization (GO:0008104)	1.77	1.36E-05
cellular localization (GO:0051641)	1.76	5.32E-07
catabolic process (GO:0009056)	1.76	1.05E-06
cellular protein metabolic process (GO:0044267)	1.70	5.73E-11
cellular response to stress (GO:0033554)	1.64	8.29E-03
macromolecule localization (GO:0033036)	1.62	1.70E-04
cellular nitrogen compound metabolic process (GO:0034641)	1.59	5.45E-12
organonitrogen compound metabolic process (GO:1901564)	1.58	5.52E-12
organic substance transport (GO:0071702)	1.56	8.92E-04
cellular metabolic process (GO:0044237)	1.53	1.80E-19
gene expression (GO:0010467)	1.51	2.59E-07
protein metabolic process (GO:0019538)	1.50	1.05E-07
heterocycle metabolic process (GO:0046483)	1.48	2.70E-07
nitrogen compound metabolic process (GO:0006807)	1.47	2.12E-14
nucleobase-containing compound metabolic process (GO:0006139)	1.47	5.11E-07
biosynthetic process (GO:0009058)	1.47	1.29E-06
cellular biosynthetic process (GO:0044249)	1.47	1.54E-06

cellular macromolecule metabolic process (GO:0044260)	1.46	2.94E-10
cellular aromatic compound metabolic process (GO:0006725)	1.46	5.39E-07
organic substance biosynthetic process (GO:1901576)	1.46	2.13E-06
organic cyclic compound metabolic process (GO:1901360)	1.44	1.03E-06
cellular nitrogen compound biosynthetic process (GO:0044271)	1.44	4.86E-05
primary metabolic process (GO:0044238)	1.42	3.86E-13
metabolic process (GO:0008152)	1.41	6.86E-14
organic substance metabolic process (GO:0071704)	1.41	3.71E-13
cellular component organization or biogenesis (GO:0071840)	1.41	9.94E-07
protein modification process (GO:0036211)	1.41	3.81E-03
cellular protein modification process (GO:0006464)	1.41	3.83E-03
establishment of localization (GO:0051234)	1.40	1.10E-04
macromolecule metabolic process (GO:0043170)	1.39	1.04E-09
organelle organization (GO:0006996)	1.39	1.23E-04
transport (GO:0006810)	1.39	1.44E-04
RNA metabolic process (GO:0016070)	1.39	4.83E-04
macromolecule modification (GO:0043412)	1.39	3.97E-03
cellular component organization (GO:0016043)	1.38	1.27E-05
nucleic acid metabolic process (GO:0090304)	1.36	5.17E-04
cellular macromolecule biosynthetic process (GO:0034645)	1.35	2.04E-03
macromolecule biosynthetic process (GO:0009059)	1.35	2.05E-03
localization (GO:0051179)	1.34	2.16E-04
cellular process (GO:0009987)	1.26	5.54E-12
biological_process (GO:0008150)	1.19	5.62E-09
Unclassified (UNCLASSIFIED)	0.86	5.37E-09
response to stimulus (GO:0050896)	0.75	2.37E-04
cell communication (GO:0007154)	0.71	4.70E-04
signaling (GO:0023052)	0.69	1.92E-04
signal transduction (GO:0007165)	0.66	3.88E-05
multicellular organismal process (GO:0032501)	0.58	6.77E-05
cell surface receptor signaling pathway (GO:0007166)	0.54	4.46E-04
response to external stimulus (GO:0009605)	0.38	1.70E-05
positive regulation of response to stimulus (GO:0048584)	0.38	7.37E-05
biological adhesion (GO:0022610)	0.32	5.93E-03
cell adhesion (GO:0007155)	0.32	5.96E-03
system process (GO:0003008)	0.30	3.04E-04
G protein-coupled receptor signaling pathway (GO:0007186)	0.29	2.06E-07
nervous system process (GO:0050877)	0.24	4.23E-04
immune system process (GO:0002376)	0.19	1.64E-12
positive regulation of immune system process (GO:0002684)	0.15	4.25E-06
multi-organism process (GO:0051704)	0.14	7.10E-10
immune response (GO:0006955)	0.13	4.30E-13
regulation of immune system process (GO:0002682)	0.13	6.46E-07
sensory perception (GO:0007600)	0.11	7.35E-05
lymphocyte mediated immunity (GO:0002449)	0.11	1.47E-04
leukocyte mediated immunity (GO:0002443)	0.11	1.53E-04

adaptive immune response based on somatic recombination of immune receptors built from immunoglobulin superfamily domains (GO:0002460)	0.11	1.54E-04
adaptive immune response (GO:0002250)	0.10	2.05E-05
cell activation (GO:0001775)	0.09	8.10E-06
leukocyte activation (GO:0045321)	0.09	1.12E-05
regulation of immune response (GO:0050776)	0.08	5.13E-07
positive regulation of immune response (GO:0050778)	0.08	8.78E-07
immune effector process (GO:0002252)	0.08	1.62E-06
B cell activation (GO:0042113)	0.06	3.15E-05
defense response (GO:0006952)	0.05	8.27E-11
lymphocyte activation (GO:0046649)	0.05	3.39E-06
innate immune response (GO:0045087)	0.04	1.48E-07
response to biotic stimulus (GO:0009607)	0.03	1.16E-10
response to other organism (GO:0051707)	0.03	1.21E-10
response to external biotic stimulus (GO:0043207)	0.03	1.30E-10
defense response to other organism (GO:0098542)	0.03	3.24E-09
response to bacterium (GO:0009617)	< 0.01	1.14E-08
activation of immune response (GO:0002253)	< 0.01	1.46E-07
humoral immune response (GO:0006959)	< 0.01	1.47E-07
immune response-regulating signaling pathway (GO:0002764)	< 0.01	1.84E-07
defense response to bacterium (GO:0042742)	< 0.01	1.91E-07
immune response-activating signal transduction (GO:0002757)	< 0.01	2.58E-07
immune response-activating cell surface receptor signaling pathway (GO:0002429)	< 0.01	6.58E-07
immune response-regulating cell surface receptor signaling pathway (GO:0002768)	< 0.01	6.59E-07
regulation of cell activation (GO:0050865)	< 0.01	1.24E-06
regulation of leukocyte activation (GO:0002694)	< 0.01	1.83E-06
antigen receptor-mediated signaling pathway (GO:0050851)	< 0.01	1.87E-06
phagocytosis (GO:0006909)	< 0.01	2.48E-06
regulation of lymphocyte activation (GO:0051249)	< 0.01	2.61E-06
positive regulation of cell activation (GO:0050867)	< 0.01	5.08E-06
positive regulation of leukocyte activation (GO:0002696)	< 0.01	5.16E-06
sensory perception of chemical stimulus (GO:0007606)	< 0.01	6.93E-06
positive regulation of lymphocyte activation (GO:0051251)	< 0.01	7.08E-06
B cell receptor signaling pathway (GO:0050853)	< 0.01	1.37E-05
immunoglobulin mediated immune response (GO:0016064)	< 0.01	1.92E-05
complement activation (GO:0006956)	< 0.01	1.94E-05

Supplementary Table 3: Classes of proteins that are enriched or depleted of the genes that had altered expression in the cortex of Chrm1-/- compared to wild type mice.

Settings:
Analysis Type: PANTHER Overrepresentation Test (Released 20200728)
Annotation Version and Release Date: PANTHER version 15.0 Released 2020-02-14
Analyzed List: Client Text Box Input (Mus musculus)
Reference List: Mus musculus (all genes in database)
Protein Class
Test Type: FISHER
Correction: FDR

PANTHER GO-Slim Biological Process	Fold Enrichment	p (FDR α 0.01)
chaperonin (PC00073)	5.01	4.81E-02
SNARE protein (PC00034)	4.62	1.46E-03
chaperone (PC00072)	3.52	1.49E-04
DNA-directed RNA polymerase (PC00019)	3.36	3.84E-02
reductase (PC00198)	3.07	1.24E-02
general transcription factor (PC00259)	2.95	4.44E-04
ribosomal protein (PC00202)	2.87	1.04E-05
translational protein (PC00263)	2.53	4.00E-07
dehydrogenase (PC00092)	2.41	4.32E-03
kinase (PC00137)	2.22	1.27E-02
oxidoreductase (PC00176)	2.03	1.65E-05
RNA binding protein (PC00031)	1.91	2.53E-06
transferase (PC00220)	1.72	1.03E-03
nucleic acid binding protein (PC00171)	1.70	1.11E-05
membrane traffic protein (PC00150)	1.68	8.37E-03
metabolite interconversion enzyme (PC00262)	1.62	1.63E-07
gene-specific transcriptional regulator (PC00264)	0.66	1.20E-02
DNA-binding transcription factor (PC00218)	0.60	3.77E-03
transmembrane signal receptor (PC00197)	0.48	4.74E-04
G-protein coupled receptor (PC00021)	0.39	2.19E-04
intercellular signal molecule (PC00207)	0.34	2.50E-03
serine protease (PC00203)	0.34	2.39E-02
helix-turn-helix transcription factor (PC00116)	0.24	7.32E-03
defense/immunity protein (PC00090)	< 0.01	1.07E-17
immunoglobulin receptor superfamily (PC00124)	< 0.01	6.77E-04
immunoglobulin (PC00123)	< 0.01	4.25E-11
oxygenase (PC00177)	< 0.01	3.23E-02

Supplementary Table 4: Grouping of genes with changed levels of expression in the cortex of the muscarinic M1 receptor knockout (Chrm1-/-) compared to wild type (w/t) mice into common functional domains.

Function Domain	Gene Name	Gene Symbol	Fold CHRM1$^{-/-}$ / wt	p
Mitochondria	mitochondrial inner membrane organizing system 1	Minos1	26.571	<0.0001
	translocase of outer mitochondrial membrane 7 homolog (yeast)	Tomm7	13.367	<0.0001
	NADH dehydrogenase (ubiquinone) 1 alpha subcomplex, 1	Ndufa1	7.940	<0.0001
	mitochondrial ribosomal protein S34	Mrps34	7.232	<0.0001
	mitochondrial ribosomal protein S12	Mrps12	6.112	<0.0001
	mitochondrial ribosomal protein S18C	Mrps18c	5.081	<0.0001
	translocase of inner mitochondrial membrane 10	Timm10	4.708	<0.0001
	NADH dehydrogenase (ubiquinone) 1 beta subcomplex, 7	Ndufb7	4.518	<0.0001
	NADH dehydrogenase (ubiquinone) 1 beta subcomplex 8	Ndufb8	4.426	<0.0001
	mitochondrial ribosomal protein S17	Mrps17	4.053	<0.0001
	translocase of outer mitochondrial membrane 40 homolog-like (yeast)	Tomm40l	3.729	<0.0001
	mitochondrial ribosomal protein S26	Mrps26	3.701	<0.0001
	mitochondrial ribosomal protein L2	Mrpl2	3.695	<0.0001
	NADH dehydrogenase (ubiquinone) 1 alpha subcomplex, assembly factor 2	Ndufaf2	3.488	<0.0001
	mitochondrial ribosomal protein L24	Mrpl24	3.473	<0.0001
	mitochondrial ribosomal protein L41	Mrpl41	3.235	<0.0001
	ATP synthase, H+ transporting, mitochondrial F1 complex, O subunit	Atp5o	3.055	<0.0001
	NADH dehydrogenase (ubiquinone) 1 alpha subcomplex, 5	Ndufa5	2.623	<0.0001
	mitochondrial ribosomal protein L11	Mrpl11	2.531	<0.0001
	translocase of inner mitochondrial membrane 44	Timm44	2.424	<0.0001
	translocase of inner mitochondrial membrane 17a	Timm17a	2.421	<0.0001
	mitochondrial ribosomal protein L55	Mrpl55	2.362	<0.0001
	mitochondrial ribosomal protein L14	Mrpl14	2.340	<0.0001
	mitochondrial ribosomal protein L9	Mrpl9	2.316	<0.0001
	mitochondrial ribosomal protein S7	Mrps7	2.239	<0.0001
	NADH dehydrogenase (ubiquinone) Fe-S protein 2	Ndufs2	2.193	<0.0001
	NADH dehydrogenase (ubiquinone) 1 alpha subcomplex, 6 (B14)	Ndufa6	2.126	<0.0001

NADH dehydrogenase (ubiquinone) 1 alpha subcomplex, 8	Ndufa8	2.122	<0.0001
ATP5S-like	Atp5sl	2.110	<0.0001
NADH dehydrogenase (ubiquinone) Fe-S protein 1	Ndufs1	2.092	<0.0001
mitochondrial amidoxime reducing component 2	Marc2	2.078	<0.0001
NADH dehydrogenase (ubiquinone) 1 beta subcomplex, 2	Ndufb2	2.065	<0.0001
translocase of outer mitochondrial membrane 70 homolog A (yeast)	Tomm70a	1.997	<0.0001
NADH dehydrogenase (ubiquinone) 1 alpha subcomplex, 9	Ndufa9	1.996	<0.0001
translocase of inner mitochondrial membrane 13	Timm13	1.982	<0.0001
mitochondrial ribosomal protein S25	Mrps25	1.924	<0.0001
ATP synthase, H+ transporting, mitochondrial F0 complex, subunit C3 (subunit 9)	Atp5g3	1.901	<0.0001
translocase of inner mitochondrial membrane 22	Timm22	1.887	<0.0001
NADH dehydrogenase (ubiquinone) 1 beta subcomplex, 11	Ndufb11	1.832	<0.0001
mitochondrial ribosomal protein S10	Mrps10	1.800	0.0008
NADH dehydrogenase (ubiquinone) 1 alpha subcomplex assembly factor 7	Ndufaf7	1.780	<0.0001
mitochondrial ribosomal protein L20	Mrpl20	1.774	<0.0001
NADH dehydrogenase (ubiquinone) 1 alpha subcomplex, assembly factor 4	Ndufaf4	1.755	<0.0001
NADH dehydrogenase (ubiquinone) Fe-S protein 6	Ndufs6	1.752	0.0049
mitochondrial ribosomal protein S28	Mrps28	1.734	<0.0001
mitochondrial GTPase 1 homolog (S. cerevisiae)	Mtg1	1.704	<0.0001
mitochondrial ribosomal protein S23	Mrps23	1.697	<0.0001
ATP synthase, H+ transporting, mitochondrial F0 complex, subunit F2	Atp5j2	1.694	0.0014
mitochondrial ribosomal protein L38	Mrpl38	1.689	<0.0001
mitochondrial ribosomal protein S31	Mrps31	1.658	<0.0001
2,4-dienoyl CoA reductase 1, mitochondrial	Decr1	1.656	<0.0001
mitochondrial ribosomal protein L46	Mrpl46	1.642	<0.0001
mitochondrial ribosomal protein L28	Mrpl28	1.602	<0.0001
NADH dehydrogenase (ubiquinone) 1 beta subcomplex, 5	Ndufb5	1.572	<0.0001
mitochondrial ribosomal protein S18B	Mrps18b	1.566	<0.0001
NADH dehydrogenase (ubiquinone) complex I, assembly factor 5	Ndufaf5	1.549	<0.0001
mitochondrial ribosomal protein S18A	Mrps18a	1.545	<0.0001
mitochondrial ribosomal protein L45	Mrpl45	1.530	<0.0001
mitochondrial ribosomal protein L1	Mrpl1	1.527	<0.0001
translocase of inner mitochondrial membrane 17b	Timm17b	1.526	<0.0001
ATP synthase, H+ transporting, mitochondrial F1 complex, gamma polypeptide 1	Atp5c1	1.513	<0.0001

	mitochondrial ribosomal protein L34	Mrpl34	0.499	<0.0001
	NADH dehydrogenase (ubiquinone) Fe-S protein 7	Ndufs7	0.469	<0.0001
	NADH dehydrogenase (ubiquinone) 1, subcomplex unknown, 2	Ndufc2	0.377	<0.0001
Protein Degradation	ubiquitin specific peptidase 17-like D	Usp17ld	13.603	<0.0001
	proteasome maturation protein	Pomp	7.603	<0.0001
	proteasome (prosome, macropain) subunit, beta type 6	Psmb6	4.160	<0.0001
	F-box protein 30	Fbxo30	4.019	<0.0001
	proteasome (prosome, macropain) 26S subunit, non-ATPase, 4	Psmd4	3.511	<0.0001
	proteasome (prosome, macropain) 26S subunit, ATPase 2	Psmc2	3.446	<0.0001
	proteasome (prosome, macropain) subunit, alpha type 7	Psma7	3.324	<0.0001
	proteasome (prosome, macropain) assembly chaperone 1	Psmg1	3.300	<0.0001
	f-box protein 9	Fbxo9	3.052	<0.0001
	SUMO/sentrin specific peptidase 8	Senp8	2.993	<0.0001
	ubiquitin-conjugating enzyme E2W (putative)	Ube2w	2.739	<0.0001
	F-box protein 45	Fbxo45	2.714	<0.0001
	ubiquitin-conjugating enzyme E2M	Ube2m	2.702	<0.0001
	ubiquitin-conjugating enzyme E2E 1	Ube2e1	2.577	<0.0001
	proteasome (prosome, macropain) subunit, beta type 1	Psmb1	2.515	<0.0001
	ubiquitin specific peptidase 5 (isopeptidase T)	Usp5	2.507	<0.0001
	proteasome (prosome, macropain) activator subunit 1 (PA28 alpha)	Psme1	2.505	<0.0001
	proteasome (prosome, macropain) subunit, alpha type 3	Psma3	2.477	<0.0001
	ubiquitin carboxy-terminal hydrolase L1	Uchl1	2.445	<0.0001
	SUMO/sentrin specific peptidase 2	Senp2	2.175	<0.0001
	proteasome (prosome, macropain) 26S subunit, ATPase 3	Psmc3	2.146	<0.0001
	proteasome (prosome, macropain) 26S subunit, non-ATPase, 6	Psmd6	2.125	<0.0001
	ubiquitin fusion degradation 1 like	Ufd1l	2.117	<0.0001
	ubiquitin-like 5	Ubl5	1.993	<0.0001
	proteasome (prosome, macropain) 26S subunit, non-ATPase, 3	Psmd3	1.986	<0.0001
	F-box protein 42	Fbxo42	1.964	<0.0001
	proteasome (prosome, macropain) subunit, alpha type 2	Psma2	1.940	<0.0001
	ubiquitin-conjugating enzyme E2 variant 2	Ube2v2	1.903	<0.0001
	proteasome (prosome, macropain) 26S subunit, non-ATPase, 11	Psmd11	1.871	<0.0001
	F-box protein 2	Fbxo2	1.815	<0.0001

	ubiquitin-conjugating enzyme E2N	Ube2n	1.808	<0.0001
	ubiquitin-fold modifier conjugating enzyme 1	Ufc1	1.808	<0.0001
	ubiquitin specific peptidase 29	Usp29	1.774	<0.0001
	ubiquitin protein ligase E3A	Ube3a	1.756	<0.0001
	ubiquitin-conjugating enzyme E2H	Ube2h	1.752	<0.0001
	proteasome (prosome, macropain) 26S subunit, non-ATPase, 10	Psmd10	1.748	<0.0001
	proteasome (prosome, macropain) 26S subunit, non-ATPase, 7	Psmd7	1.624	<0.0001
	ubiquitin family domain containing 1	Ubfd1	1.615	<0.0001
	ubiquitin associated domain containing 1	Ubac1	1.614	<0.0001
	F-box protein 31	Fbxo31	1.612	<0.0001
	F-box protein 6	Fbxo6	1.602	<0.0001
	SUMO/sentrin specific peptidase 3	Senp3	1.551	<0.0001
	ubiquitin-conjugating enzyme E2Q (putative) 2	Ube2q2	1.537	<0.0001
	proteasome (prosome, macropain) 26S subunit, non-ATPase, 13	Psmd13	1.534	<0.0001
	ubiquitin-like modifier activating enzyme 5	Uba5	1.531	<0.0001
	proteasome (prosome, macropain) 26S subunit, ATPase, 6	Psmc6	1.529	<0.0001
	F-box protein 7	Fbxo7	1.528	<0.0001
	proteasome (prosome, macropain) subunit, beta type 10	Psmb10	1.509	<0.0001
	ubiquitin specific peptidase like 1	Uspl1	1.508	<0.0001
	ubiquitin-associated protein 2	Ubap2	0.492	<0.0001
	ubiquitin specific peptidase 54	Usp54	0.428	<0.0001
	ubiquitin-associated protein 2-like	Ubap2l	0.372	<0.0001
Membrane Transport	solute carrier family 40 (iron-regulated transporter), member 1	Slc40a1	3.440	<0.0001
	solute carrier family 33 (acetyl-CoA transporter), member 1	Slc33a1	2.870	<0.0001
	ATP-binding cassette, sub-family B (MDR/TAP), member 1A	Abcb1a	2.650	<0.0001
	solute carrier family 39 (zinc transporter), member 12	Slc39a12	2.530	<0.0001
	solute carrier family 35 (UDP-N-acetylglucosamine (UDP-GlcNAc) transporter), member 3	Slc35a3	2.490	<0.0001
	ATP-binding cassette, sub-family B (MDR/TAP), member 8	Abcb8	2.490	<0.0001
	solute carrier family 5 (sodium-dependent vitamin transporter), member 6	Slc5a6	2.470	<0.0001
	solute carrier family 25 (mitochondrial carrier, phosphate carrier), member 25	Slc25a25	2.370	<0.0001
	solute carrier family 16 (monocarboxylic acid transporters), member 1	Slc16a1	2.360	<0.0001
	solute carrier family 25 (mitochondrial carrier, brain), member 14	Slc25a14	2.160	<0.0001

	solute carrier family 13 (sodium-dependent citrate transporter), member 5	Slc13a5	2.120	<0.0001
	solute carrier family 25, member 33	Slc25a33	2.120	<0.0001
	solute carrier family 35, member E3	Slc35e3	2.010	<0.0001
	ATP-binding cassette, sub-family F (GCN20), member 3	Abcf3	1.910	<0.0001
	solute carrier family 37 (glycerol-3-phosphate transporter), member 3	Slc37a3	1.900	<0.0001
	solute carrier family 6 (neurotransmitter transporter, L-proline), member 7	Slc6a7	1.900	<0.0001
	solute carrier family 35 (CMP-sialic acid transporter), member 1	Slc35a1	1.880	<0.0001
	ATP-binding cassette, sub-family F (GCN20), member 2	Abcf2	1.810	<0.0001
	solute carrier family 25 (mitochondrial carrier, adenine nucleotide translocator), member 4	Slc25a4	1.760	<0.0001
	solute carrier family 39 (zinc transporter), member 10	Slc39a10	1.720	<0.0001
	ATP-binding cassette, sub-family B (MDR/TAP), member 6	Abcb6	1.620	<0.0001
	solute carrier family 4, sodium bicarbonate cotransporter, member 5	Slc4a5	1.560	<0.0001
	solute carrier family 35, member A5	Slc35a5	1.550	<0.0001
	solute carrier family 7 (cationic amino acid transporter, y+ system), member 4	Slc7a4	1.540	<0.0001
	solute carrier family 9 (sodium/hydrogen exchanger), member 6	Slc9a6	1.530	<0.0001
	solute carrier family 4 (anion exchanger), member 8	Slc4a8	1.520	<0.0001
	solute carrier family 45, member 1	Slc45a1	1.510	<0.0001
	solute carrier family 5 (inositol transporters), member 3	Slc5a3	1.510	0.0001
	solute carrier family 27 (fatty acid transporter), member 4	Slc27a4	0.490	<0.0001
	solute carrier family 25, member 37	Slc25a37	0.480	<0.0001
	solute carrier family 20, member 1	Slc20a1	0.460	<0.0001
	solute carrier family 7 (cationic amino acid transporter, y+ system), member 10	Slc7a10	0.460	<0.0001
	solute carrier family 39 (metal ion transporter), member 13	Slc39a13	0.450	<0.0001
	solute carrier family 38, member 6	Slc38a6	0.430	<0.0001
	solute carrier family 9 (sodium/hydrogen exchanger), member 1	Slc9a1	0.420	<0.0001
	solute carrier family 36 (proton/amino acid symporter), member 1	Slc36a1	0.400	<0.0001
	solute carrier family 6 (neurotransmitter transporter, creatine), member 8	Slc6a8	0.150	<0.0001
Neurotransmission	gamma-aminobutyric acid (GABA) A receptor-associated protein-like 2	Gabarapl2	2.731	<0.0001
	syntaxin 7	Stx7	2.689	<0.0001
	adrenergic receptor, beta 1	Adrb1	2.307	<0.0001
	5-hydroxytryptamine (serotonin) receptor 2C	Htr2c	2.116	<0.0001
	syntaxin 1A (brain)	Stx1a	2.040	<0.0001

	glutamate dehydrogenase 1	Glud1	1.967	<0.0001
	synaptosomal-associated protein, 47	Snap47	1.892	<0.0001
	syntaxin 17	Stx17	1.658	<0.0001
	syntaxin 4A (placental)	Stx4a	1.647	<0.0001
	syntaxin 16	Stx16	1.579	<0.0001
	syntaxin 18	Stx18	1.564	<0.0001
	syntaxin 1B	Stx1b	0.476	<0.0001
	acetylcholinesterase	Ache	0.445	0.0003
	cholinergic receptor, nicotinic, alpha polypeptide 4	Chrna4	0.413	<0.0001
	5-hydroxytryptamine (serotonin) receptor 1B	Htr1b	0.341	<0.0001
	gamma-aminobutyric acid (GABA) A receptor, subunit beta 3	Gabrb3	0.321	<0.0001
	dopamine receptor D2	Drd2	0.196	<0.0001
	cholinergic receptor, muscarinic 1, CNS	Chrm1	0.097	<0.0001
Receptor Signalling	tetraspanin 15	Tspan15	2.474	<0.0001
	regulator of G-protein signaling 4	Rgs4	2.265	<0.0001
	regulator of G-protein signaling 14	Rgs14	2.130	<0.0001
	guanine nucleotide binding protein (G protein), alpha inhibiting 3	Gnai3	1.938	<0.0001
	tetraspanin 12	Tspan12	1.844	<0.0001
	regulator of G-protein signaling 17	Rgs17	1.757	<0.0001
	adenylate kinase 4	Ak4	1.657	0.0008
	tetraspanin 3	Tspan3	1.591	<0.0001
	tetraspanin 2	Tspan2	1.534	<0.0001
	guanine nucleotide binding protein (G protein), beta 4	Gnb4	1.530	0.0005
	tetraspanin 4	Tspan4	1.529	<0.0001
	adenylate kinase 2	Ak2	1.505	<0.0001
	regulator of G-protein signalling 10	Rgs10	0.485	<0.0001
	guanine nucleotide binding protein (G protein), gamma 4	Gng4	0.475	<0.0001
	regulator of G-protein signaling 2	Rgs2	0.464	<0.0001
	tetraspanin 7	Tspan7	0.427	<0.0001
Ribosomal Function	ribosomal protein S4-like	Rps4l	3.745	<0.0001
	ribosomal protein S3	Rps3	3.602	<0.0001
	ribosomal protein S14	Rps14	3.431	<0.0001

	ribosomal protein L31	Rpl31	3.352	<0.0001
	ribosomal protein S9	Rps9	3.201	<0.0001
	ribosomal protein L22	Rpl22	2.689	<0.0001
	ribosomal protein L8	Rpl8	2.628	<0.0001
	DEAD/H (Asp-Glu-Ala-Asp/His) box polypeptide 3, X-linked	Ddx3x	2.393	<0.0001
	DEAD (Asp-Glu-Ala-Asp) box polypeptide 3, Y-linked	Ddx3y	2.351	<0.0001
	DEAD (Asp-Glu-Ala-Asp) box polypeptide 42	Ddx42	2.190	<0.0001
	ribosomal protein L37	Rpl37	2.045	<0.0001
	DEAD (Asp-Glu-Ala-Asp) box polypeptide 20	Ddx20	1.961	<0.0001
	ribosomal protein SA	Rpsa	1.919	<0.0001
	ribosomal protein L21	Rpl21	1.850	<0.0001
	DEAD (Asp-Glu-Ala-Asp) box polypeptide 25	Ddx25	1.845	<0.0001
	DEAD (Asp-Glu-Ala-Asp) box polypeptide 5	Ddx5	1.652	<0.0001
	ribosomal protein S6 kinase-like 1	Rps6kl1	1.534	<0.0001
	ribosomal protein L30	Rpl30	1.528	0.0003
	ribosomal protein S13	Rps13	1.518	<0.0001
	DEAD (Asp-Glu-Ala-Asp) box polypeptide 28	Ddx28	1.512	<0.0001
	ribosomal protein S19 binding protein 1	Rps19bp1	1.512	<0.0001
	ribosomal protein S15A	Rps15a	1.500	<0.0001
	ribosomal protein S27-like	Rps27l	0.446	<0.0001
	DEAD/H (Asp-Glu-Ala-Asp/His) box polypeptide 26B	Ddx26b	0.395	<0.0001
Calcium Biochemistry	S100 calcium binding protein A6 (calcyclin)	S100a6	2.502	<0.0001
	S100 protein, beta polypeptide, neural	S100b	2.177	<0.0001
	calcium regulated heat stable protein 1	Carhsp1	2.111	<0.0001
	calcium binding protein 1	Cabp1	1.716	<0.0001
	calcium and integrin binding family member 2	Cib2	1.700	<0.0001
	calmodulin 3	Calm3	1.683	<0.0001
	calcium/calmodulin-dependent protein kinase I	Camk1	1.675	<0.0001
	calcium response factor	Carf	1.556	<0.0001
	S100 calcium binding protein A13	S100a13	1.536	<0.0001
	calcium channel flower domain containing 1	Cacfd1	1.503	<0.0001
	calcium channel, voltage-dependent, gamma subunit 4	Cacng4	0.452	<0.0001
	calcium binding protein 39	Cab39	0.441	<0.0001

	S100 calcium binding protein A1	S100a1	0.355	<0.0001
	calcium binding and coiled coil domain 1	Calcoco1	0.266	<0.0001
Phosphorylation	protein phosphatase 4, regulatory subunit 2	Ppp4r2	3.002	<0.0001
	protein phosphatase 2, regulatory subunit B, delta	Ppp2r2d	2.626	<0.0001
	protein phosphatase 1K (PP2C domain containing)	Ppm1k	2.401	<0.0001
	protein phosphatase 1F (PP2C domain containing)	Ppm1f	1.891	<0.0001
	protein phosphatase 1, regulatory (inhibitor) subunit 11	Ppp1r11	1.771	<0.0001
	protein phosphatase 1G (formerly 2C), magnesium-dependent, gamma isoform	Ppm1g	1.723	<0.0001
	protein phosphatase 1, regulatory (inhibitor) subunit 16A	Ppp1r16a	1.683	<0.0001
	protein phosphatase 1, regulatory (inhibitor) subunit 8	Ppp1r8	1.682	<0.0001
	protein phosphatase 4, catalytic subunit	Ppp4c	1.663	<0.0001
	protein phosphatase 5, catalytic subunit	Ppp5c	1.645	<0.0001
	protein phosphatase 1A, magnesium dependent, alpha isoform	Ppm1a	1.624	<0.0001
	protein phosphatase 2, regulatory subunit B, beta	Ppp2r2b	1.540	<0.0001
	protein phosphatase 1, regulatory (inhibitor) subunit 3C	Ppp1r3c	1.523	<0.0001
	protein phosphatase 1, regulatory (inhibitor) subunit 1A	Ppp1r1a	1.502	0.0002
Heat Shock	DnaJ (Hsp40) homolog, subfamily C, member 12	Dnajc12	4.370	<0.0001
	DnaJ (Hsp40) homolog, subfamily C, member 10	Dnajc10	2.378	<0.0001
	DnaJ (Hsp40) homolog, subfamily A, member 1	Dnaja1	2.207	0.0005
	DnaJ (Hsp40) homolog, subfamily C, member 25	Dnajc25	2.086	<0.0001
	DnaJ (Hsp40) homolog, subfamily C, member 3	Dnajc3	2.067	<0.0001
	DnaJ (Hsp40) homolog, subfamily C, member 11	Dnajc11	1.991	<0.0001
	DnaJ (Hsp40) homolog, subfamily C, member 18	Dnajc18	1.794	<0.0001
	DnaJ (Hsp40) homolog, subfamily C, member 9	Dnajc9	1.703	<0.0001
Redox reactions	hydroxyacyl glutathione hydrolase	Hagh	2.894	<0.0001
	glutathione S-transferase, alpha 4	Gsta4	2.807	<0.0001
	glutathione S-transferase, pi 1	Gstp1	2.708	<0.0001
	hydroxyacylglutathione hydrolase-like	Haghl	2.088	<0.0001
	glutathione S-transferase omega 1	Gsto1	2.025	<0.0001
	glutathione S-transferase, mu 5	Gstm5	1.886	<0.0001
	glutathione peroxidase 1	Gpx1	1.543	<0.0001

	glutathione S-transferase, mu 4	Gstm4	1.537	<0.0001
Tubulin	tubulin folding cofactor B	Tbcb	2.432	<0.0001
	tubulin, beta 2A class IIA	Tubb2a	2.157	<0.0001
	tubulin polyglutamylase complex subunit 1	Tpgs1	2.078	<0.0001
	tubulin-specific chaperone C	Tbcc	1.803	<0.0001
	tubulin-specific chaperone d	Tbcd	1.801	<0.0001
	tubulin, alpha 1A	Tuba1a	1.768	<0.0001
	tubulin polymerization promoting protein	Tppp	1.749	<0.0001
	tubulin, beta 2B class IIB	Tubb2b	1.659	<0.0001
Inflammation	interferon-related developmental regulator 1	Ifrd1	3.571	<0.0001
	tumor necrosis factor alpha induced protein 6	Tnfaip6	2.458	<0.0001
	TNF receptor-associated protein 1	Trap1	2.082	<0.0001
	interleukin 34	Il34	1.778	<0.0001
	interferon, alpha-inducible protein 27	Ifi27	1.721	<0.0001
	chemokine (C-X3-C motif) receptor 1	Cx3cr1	1.571	<0.0001
	TGF-beta activated kinase 1/MAP3K7 binding protein 2	Tab2	0.458	<0.0001
Energy and Metabolism	succinate-CoA ligase, GDP-forming, alpha subunit	Suclg1	3.199	<0.0001
	pyruvate dehydrogenase complex, component X	Pdhx	2.641	<0.0001
	pyruvate dehydrogenase E1 alpha 1	Pdha1	2.587	<0.0001
	succinate dehydrogenase complex, subunit D, integral membrane protein	Sdhd	2.202	<0.0001
	succinate dehydrogenase complex, subunit B, iron sulfur (Ip)	Sdhb	1.710	<0.0001
	pyruvate dehydrogenase kinase, isoenzyme 1	Pdk1	1.625	<0.0001
	succinate-Coenzyme A ligase, ADP-forming, beta subunit	Sucla2	1.518	<0.0001
Lipid Metabolism	acyl-Coenzyme A binding domain containing 5	Acbd5	2.804	<0.0001
	acyl-Coenzyme A dehydrogenase family, member 8	Acad8	2.343	<0.0001
	acyl-Coenzyme A dehydrogenase family, member 9	Acad9	1.950	<0.0001
	acyl-Coenzyme A oxidase 1, palmitoyl	Acox1	1.748	<0.0001
	acyl-Coenzyme A binding domain containing 4	Acbd4	1.587	<0.0001
	acyl-Coenzyme A dehydrogenase, medium chain	Acadm	1.541	<0.0001

Supplementary Table 5: Genes with changed levels of expression in the cortex of muscarinic M1 receptor knockout ($Chrm1^{-/-}$) compared to wild type mice (w/t) mice and in Brodmann's area 10 from patients with schizophrenia (Sz) compared to controls (Cont).

Gene Name	Murine Gene Symbol	Murine Fold $CHRM1^{-/-}$ / wt	Murine p	Human Gene Symbol	Human Fold Sz / Cont	Human p
AKT interacting protein	Aktip	1.575	<0.0001	AKTIP	0.774	0.0083
nuclear receptor subfamily 1, group D, member 2	Nr1d2	1.706	<0.0001	NR1D2	0.782	0.0046
acyl-Coenzyme A dehydrogenase, medium chain	Acadm	1.541	<0.0001	ACADM	0.728	0.0031
coiled-coil domain containing 53	Ccdc53	0.310	<0.0001	CCDC53	0.736	0.0035
centrin 3	Cetn3	1.550	<0.0001	CETN3	0.755	0.0071
dynactin 6	Dctn6	0.433	<0.0001	DCTN6	0.779	0.0067
D-dopachrome tautomerase	Ddt	1.539	<0.0001	DDT	0.785	0.0058
dihydrolipoamide dehydrogenase	Dld	1.588	<0.0001	DLD	0.760	0.0048
DnaJ (Hsp40) homolog, subfamily C, member 3	Dnajc3	2.067	<0.0001	DNAJC3	0.755	0.0064
eukaryotic translation initiation factor 3, subunit E	Eif3e	4.473	<0.0001	EIF3E	0.680	0.0022
fatty acid binding protein 7, brain	Fabp7	0.072	<0.0001	FABP7	0.682	0.0034
glyoxalase 1	Glo1	3.767	<0.0001	GLO1	0.721	0.0027
general transcription factor IIH, polypeptide 5	Gtf2h5	1.746	<0.0001	GTF2H5	0.723	0.0007
5-hydroxytryptamine (serotonin) receptor 1B	Htr1b	0.342	<0.0001	HTR1B	1.256	0.0088
intraflagellar transport 57	Ift57	2.003	<0.0001	IFT57	0.781	0.0025
KRR1, small subunit (SSU) processome component, homolog (yeast)	Krr1	2.084	<0.0001	KRR1	0.763	0.0098
lactate dehydrogenase B	Ldhb	1.525	<0.0001	LDHB	0.796	0.0082
malignant T cell amplified sequence 1	Mcts1	2.084	<0.0001	MCTS1	0.759	0.0003
methylmalonic aciduria (cobalamin deficiency) cblD type, with homocystinuria	Mmadhc	2.250	<0.0001	MMADHC	0.740	0.0034
NADH dehydrogenase (ubiquinone) 1 alpha subcomplex, 5	Ndufa5	2.623	<0.0001	NDUFA5	0.704	0.0026
neuroguidin, EIF4E binding protein	Ngdn	1.713	<0.0001	NGDN	0.792	0.0062
NOP58 ribonucleoprotein	Nop58	2.380	<0.0001	NOP58	0.751	0.0059
poly (A) polymerase alpha	Papola	0.478	<0.0001	PAPOLA	0.785	0.0082
progestin and adipoQ receptor family member VII	Paqr7	2.433	<0.0001	PAQR7	1.208	<0.0001
programmed cell death 10	Pdcd10	5.281	<0.0001	PDCD10	0.784	0.0098
pleiotropic regulator 1, PRL1 homolog (Arabidopsis)	Plrg1	1.928	<0.0001	PLRG1	0.786	0.0073

patatin-like phospholipase domain containing 8	Pnpla8	1.915	<0.0001	PNPLA8	0.668	0.0033
polymerase (RNA) II (DNA directed) polypeptide B	Polr2b	2.444	<0.0001	POLR2B	0.768	0.0088
peroxiredoxin 3	Prdx3	2.258	<0.0001	PRDX3	0.729	0.0018
proteasome (prosome, macropain) subunit, alpha type 2	Psma2	1.940	<0.0001	PSMA2	0.729	0.0018
regulator of G-protein signaling 2	Rgs2	0.464	<0.0001	RGS2	0.788	0.0064
RNA polymerase II associated protein 3	Rpap3	3.138	<0.0001	RPAP3	0.748	0.0084
succinate dehydrogenase complex, subunit D, integral membrane protein	Sdhd	2.202	<0.0001	SDHD	0.694	0.004
SEC11 homolog C, signal peptidase complex subunit	Sec11c	3.270	<0.0001	SEC11C	0.764	0.0028
solute carrier family 25, member 33	Slc25a33	2.119	<0.0001	SLC25A33	0.604	0.0035
sphingomyelin phosphodiesterase 3, neutral	Smpd3	0.491	<0.0001	SMPD3	1.209	0.0045
sorting nexin 2	Snx2	1.561	<0.0001	SNX2	0.691	0.0082
TGF-beta activated kinase 1/MAP3K7 binding protein 2	Tab2	0.458	<0.0001	TAB2	0.797	0.007
TAF13 RNA polymerase II, TATA box binding protein (TBP)-associated factor	Taf13	3.615	<0.0001	TAF13	0.702	0.0052
transferrin receptor	Tfrc	3.131	<0.0001	TFRC	0.784	0.0031
thioredoxin-related transmembrane protein 1	Tmx1	1.730	<0.0001	TMX1	0.776	0.0027
trafficking protein particle complex 2	Trappc2	1.693	<0.0001	TRAPPC2	0.745	0.0074
ubiquitin-conjugating enzyme E2 variant 2	Ube2v2	1.904	<0.0001	UBE2V2	0.800	0.0089
ubiquitin-fold modifier conjugating enzyme 1	Ufc1	1.809	<0.0001	UFC1	0.766	0.0006
WW domain binding protein 5	Wbp5	5.367	<0.0001	WBP5	0.753	0.0054
X-ray repair complementing defective repair in Chinese hamster cells 6	Xrcc6	1.761	<0.0001	XRCC6	1.280	0.0093
zinc finger, CCHC domain containing 17	Zcchc17	1.653	<0.0001	ZCCHC17	0.776	0.0048

Supplementary Table 6: Genes with changed levels of expression in the muscarinic M1 receptor knockout ($Chrm1^{-/-}$) which have been identifies as associated with human cognition ability.

Gene Name	Gene Symbol	Fold $CHRM1^{-/-}$ / wt	p
activator of basal transcription 1	Abt1	1.715	<0.0001
acyl-Coenzyme A binding domain containing 4	Acbd4	1.587	<0.0001
AKT interacting protein	Aktip	1.575	<0.0001
ATPase, H+ transporting, lysosomal V0 subunit B	Atp6v0b	1.679	<0.0001
ataxin 1	Atxn1	0.467	<0.0001
ataxin 2-like	Atxn2l	0.213	<0.0001
bleomycin hydrolase	Blmh	2.13	<0.0001
calumenin	Calu	1.888	<0.0001
CaM kinase-like vesicle-associated	Camkv	1.808	<0.0001
CLP1, cleavage and polyadenylation factor I subunit	Clp1	6.737	<0.0001
catenin (cadherin associated protein), alpha 2	Ctnna2	2.086	<0.0001
DDB1 and CUL4 associated factor 11	Dcaf11	2.366	<0.0001
dystrobrevin binding protein 1	Dtnbp1	2.018	<0.0001
euchromatic histone lysine N-methyltransferase 2	Ehmt2	1.897	<0.0001
ELAV (embryonic lethal, abnormal vision, Drosophila)-like 2 (Hu antigen B)	Elavl2	1.749	<0.0001
Eph receptor A5	Epha5	1.501	<0.0001
exostoses (multiple) 1	Ext1	2.004	<0.0001
family with sequence similarity 193, member A	Fam193a	2.008	<0.0001
forkhead box P1	Foxp1	0.465	<0.0001
GATA zinc finger domain containing 2B	Gatad2b	0.363	<0.0001
golgi coiled coil 1	Gcc1	1.534	<0.0001
GDP-mannose pyrophosphorylase B	Gmppb	1.669	<0.0001
golgi SNAP receptor complex member 1	Gosr1	1.6	<0.0001
G protein-coupled receptor 26	Gpr26	1.957	<0.0001
glutathione peroxidase 1	Gpx1	1.544	<0.0001
H1 histone family, member 0	H1f0	1.612	<0.0001
histidyl-tRNA synthetase	Hars	2.845	<0.0001
hexamethylene bis-acetamide inducible 1	Hexim1	2.027	<0.0001
histone cluster 1, H1b	Hist1h1b	1.528	<0.0001
inositol hexaphosphate kinase 1	Ip6k1	1.657	<0.0001
jumping translocation breakpoint	Jtb	1.563	<0.0001
leucine rich repeat containing 24	Lrrc24	1.969	<0.0001
MAP/microtubule affinity regulating kinase 3	Mark3	1.596	<0.0001
MOB family member 4, phocein	Mob4	1.662	<0.0001
myotubularin related protein 4	Mtmr4	2.012	<0.0001
N-6 adenine-specific DNA methyltransferase 2 (putative)	N6amt2	2.954	<0.0001
NADH dehydrogenase (ubiquinone) 1 alpha subcomplex, 6 (B14)	Ndufa6	2.127	<0.0001
NADH dehydrogenase (ubiquinone) 1 alpha subcomplex, assembly factor 2	Ndufaf2	3.488	<0.0001
neural precursor cell expressed, developmentally down-regulated gene 8	Nedd8	7.614	<0.0001
neuraminidase 1	Neu1	1.756	<0.0001
nuclear receptor subfamily 1, group D, member 2	Nr1d2	1.706	<0.0001

penta-EF hand domain containing 1	Pef1	1.796	<0.0001
protein phosphatase 1, regulatory (inhibitor) subunit 16A	Ppp1r16a	1.683	<0.0001
proteasome (prosome, macropain) 26S subunit, ATPase 3	Psmc3	2.147	<0.0001
proteasome (prosome, macropain) assembly chaperone 1	Psmg1	3.3	<0.0001
glutaminyl-tRNA synthetase	Qars	1.906	<0.0001
ribosomal protein S19 binding protein 1	Rps19bp1	1.512	<0.0001
runt-related transcription factor 1	Runx1t1	0.333	<0.0001
septin 4	Sept4	1.666	<0.0001
small EDRK-rich factor 2	Serf2	1.626	<0.0001
solute carrier family 39 (metal ion transporter), member 13	Slc39a13	0.448	<0.0001
single-pass membrane protein with aspartate rich tail 1	Smdt1	1.756	<0.0001
staphylococcal nuclease and tudor domain containing 1	Snd1	2.183	<0.0001
sine oculis-binding protein homolog (Drosophila)	Sobp	0.452	<0.0001
signal peptidase complex subunit 1 homolog (S. cerevisiae)	Spcs1	5.908	<0.0001
spinster homolog 1	Spns1	2.621	<0.0001
serine/arginine-rich protein specific kinase 2	Srpk2	1.592	<0.0001
stromal antigen 1	Stag1	1.602	<0.0001
staufen (RNA binding protein) homolog 1 (Drosophila)	Stau1	0.486	<0.0001
t-complex 11 (mouse) like 2	Tcp11l2	1.816	<0.0001
T cell leukemia translocation altered gene	Tcta	1.663	<0.0001
Tp53rk binding protein	Tprkb	1.568	<0.0001
tripartite motif-containing 27	Trim27	1.618	<0.0001
ubiquitin-conjugating enzyme E2E 1	Ube2e1	2.577	<0.0001
ubiquinol-cytochrome c reductase core protein 1	Uqcrc1	2.383	<0.0001
von Willebrand factor A domain containing 5A	Vwa5a	1.524	<0.0001
yippee-like 4 (Drosophila)	Ypel4	1.555	<0.0001
zinc finger, HIT type 3	Znhit3	7.921	<0.0001
zinc finger SWIM-type containing 6	Zswim6	0.45	<0.0001

Supplementary Table 7: Genes with changed levels of expression in Brodmann's area 10 from patients with schizophrenia (Sz) compared to controls (Cont) which have been identifies as associated with human cognitive ability.

Gene Name	Gene Symbol	Fold	p
AKT interacting protein	AKTIP	Sz / Cont	0.0083
nuclear receptor subfamily 1, group D, member 2	NR1D2	0.782	0.0046
cadherin 9, type 2 (T1-cadherin)	CDH9	0.755	0.0095
carboxypeptidase N, polypeptide 1	CPN1	1.472	0.0021
La ribonucleoprotein domain family, member 7	LARP7	0.697	0.0054
MARVEL domain containing 3	MARVELD3	1.215	0.0069
musculoskeletal, embryonic nuclear protein 1	MUSTN1	1.212	0.0050
neuroepithelial cell transforming 1	NET1	1.272	0.0020
solute carrier family 44, member 4	SLC44A4	1.214	0.0062
saitohin	STH	1.498	0.0009
transmembrane and immunoglobulin domain containing 1	TMIGD1	1.204	0.0095
TNFAIP3 interacting protein 2	TNIP2	1.245	0.0003

Supplementary Table 8: Genes with changed levels of expression in the cortex of muscarinic M1 receptor knockout (CHRM1$^{-/-}$) and in and in Brodmann's area 10 from patients with schizophrenia (Sz): established links to schizophrenia

Gene Name	Gene Symbol	
AKT Interacting Protein	Aktip	Involved in breakdown in the communication across different slow and fast neurotransmitter systems through intracellular signaling pathways (Duilio *et al*, 1998).<br>Dysregulation of lysosomal function and cytoskeleton remodelling (Zhao *et al*, 2015).
coiled-coil domain containing 53	Ccdc53	Variation in DNA methylation associated with suicide attempt in schizophrenia (Bani-Fatemi *et al*, 2018).
centrin 3	Cetn3	Differentially expressed by peripheral blood leukocytes from patients with schizophrenia (Zhang *et al*, 2020).<br>Associated with altered risk for schizophrenia (Li *et al*, 2017)
dynactin 6	Dctn6	Reported as having an increased level of expression in Brodmann's area 22 from patients with schizophrenia (Schmitt *et al*, 2012).<br>Interactions with DTNBP1 which is a gene associated with an altered risk for schizophrenia (Mead *et al*, 2010).
eukaryotic translation initiation factor 3, subunit E	Eif3e	Identified as having increased levels of expression in a study combining a number of data sets from Brodmann's areas 9, 10 and 46 from patients with schizophrenia and controls (Mistry *et al*, 2013).
fatty acid binding protein 7, brain	Fabp7	Suggested to play a role in linking the NMDA receptor, neurodevelopmental, glial dysfunction and a PPI endophenotype in schizophrenia (Watanabe *et al*, 2007).<br>Fabp3 knockout (KO) mice has decreased social memory and novelty seeking a behavioural phenotype thought to be a relevant to schizophrenia (Shimamoto *et al*, 2014).<br>Involved in abnormalities of polyunsaturated fatty acids and fatty acid binding protein in the pathophysiology of schizophrenia (Maekawa *et al*, 2011).
glyoxalase 1	Glo1	GLO1 deficits and carbonyl stress are linked to the development of a certain subtype of schizophrenia (Arai *et al*, 2010).<br>Frame shift mutation in the GLO1 gene associated with oxidative stress and structural alteration of the neurites (Mizutani *et al*, 2019).
general transcription factor IIH, polypeptide 5	Gtf2h5	Higher levels of expression in peripheral blood mononuclear cells (Gardiner *et al*, 2013).

5-hydroxytryptamine (serotonin) receptor 1B	Htr1b	Polymorphisms are associated with schizophrenia in the northern Han Chinese population (Xia *et al*, 2018).<br>Increased expression in the hippocampus from patients with schizophrenia (Lopez-Figueroa *et al*, 2004).
lactate dehydrogenase B	Ldhb	Lower levels of protein in corpus callosum from patients with schizophrenia (Sivagnanasundaram *et al*, 2007).<br>Higher levels of protein in peripheral blood mononuclear cells from patients with schizophrenia (Herberth *et al*, 2011).
malignant T cell amplified sequence 1	Mcts1	Higher level of expression in blood from patients with schizophrenia (Glatt *et al*, 2011).
neuroguidin, EIF4E binding protein	Ngdn	Level of expression correlated between neuropsychological domain scores and differential gene expression in olfactory endothelial cells from patients with schizophrenia (Horiuchi *et al*, 2016).
programmed cell death 10	Pdcd10	Higher expression variability in the dorsolateral prefrontal cortex from patients with schizophrenia (Huang *et al*, 2020).
patatin-like phospholipase domain containing 8	Pnpla8	Dysregulated splicing events in Brodmann's area 10 and the caudate putamen from patients with schizophrenia (Cohen *et al*, 2012)
peroxiredoxin 3	Prdx3	Decreased protein levels in Brodmann's area 10 from patients with schizophrenia (Hirayama-Kurogi *et al*, 2017).
proteasome (prosome, macropain) subunit, alpha type 2	Psma2	Lower levels of expression in pyramidal cells from Brodmann's area 9 from patients with schizophrenia (Arion *et al*, 2015).
regulator of G-protein signaling 2	Rgs2	Genetic variation associated with symptom severity in schizophrenia (Campbell *et al*, 2008).<br>Genetic variation associated with increased risk of extrapyramidal side effects after antipsychotic drug treatment (Gareeva *et al*, 2013).
succinate dehydrogenase complex, subunit D, integral membrane protein	Sdhd	Higher levels of succinate dehydrogenase activity in the dorsolateral prefrontal cortex from patients with schizophrenia (Bubber *et al*, 2011).
solute carrier family 25, member 33	Slc25a33	Differentially expressed in the CNS from patients with schizophrenia (Jaffe *et al*, 2018)
sphingomyelin phosphodiesterase 3, neutral	Smpd3	Contains a shared expressed genetic variant in families with individuals who have an atypical psychoses (Okayama *et al*, 2018).
sorting nexin 2	Snx2	Evidence from GWAS and gene set enrichment data for and involvement in schizophrenia (Zhang *et al*, 2020).<br>Interactions with dysbindin, a susceptibility gene for schizophrenia (Gokhale *et al*, 2012).
thioredoxin-related transmembrane protein 1	Tmx1	Lower levels of expression in the blood of first episode patients (Leirer *et al*, 2019).
trafficking protein particle complex 2	Trappc2	Evidence from GWAS and gene set enrichment data for and involvement in schizophrenia (Zhang *et al*, 2020).

ubiquitin-conjugating enzyme E2 variant 2	Ube2v2	Lower in peripheral blood mononuclear cells from treatment naïve patients with schizophrenia (Kumarasinghe *et al*, 2013).
WW domain binding protein 5	Wbp5	Higher levels of expression in peripheral blood mononuclear cells from treatment naïve patients with schizophrenia (Glatt *et al*, 2005).
X-ray repair complementing defective repair in Chinese hamster cells 6	Xrcc6	Lower levels of expression in the blood of first episode patients (Leirer *et al*, 2019).

Arai M, Yuzawa H, Nohara I, Ohnishi T, Obata N, Iwayama Y*, et al* (2010). Enhanced carbonyl stress in a subpopulation of schizophrenia. *Arch Gen Psychiatry* **67**(6): 589-597.

Arion D, Corradi JP, Tang S, Datta D, Boothe F, He A*, et al* (2015). Distinctive transcriptome alterations of prefrontal pyramidal neurons in schizophrenia and schizoaffective disorder. *Mol Psychiatry* **20**(11): 1397-1405.

Bani-Fatemi A, Jeremian R, Wang KZ, Silveira J, Zai C, Kolla NJ*, et al* (2018). Epigenome-wide association study of suicide attempt in schizophrenia. *J Psychiatr Res* **104**: 192-197.

Bubber P, Hartounian V, Gibson GE, Blass JP (2011). Abnormalities in the tricarboxylic acid (TCA) cycle in the brains of schizophrenia patients. *Eur Neuropsychopharmacol* **21**(3): 254-260.

Campbell DB, Lange LA, Skelly T, Lieberman J, Levitt P, Sullivan PF (2008). Association of RGS2 and RGS5 variants with schizophrenia symptom severity. *Schizophr Res* **101**(1-3): 67-75.

Cohen OS, McCoy SY, Middleton FA, Bialosuknia S, Zhang-James Y, Liu L*, et al* (2012). Transcriptomic analysis of postmortem brain identifies dysregulated splicing events in novel candidate genes for schizophrenia. *Schizophr Res* **142**(1): 188-199.

Duilio A, Faraonio R, Minopoli G, Zambrano N, Russo T (1998). Fe65L2: a new member of the Fe65 protein family interacting with the intracellular domain of the Alzheimer's beta-amyloid precursor protein. *The Biochemical journal* **330 (Pt 1)**: 513-519.

Gardiner EJ, Cairns MJ, Liu B, Beveridge NJ, Carr V, Kelly B*, et al* (2013). Gene expression analysis reveals schizophrenia-associated dysregulation of immune pathways in peripheral blood mononuclear cells. *J Psychiatr Res* **47**(4): 425-437.

Gareeva AE, Zakirov DF, Valinurov RG, Khusnutdinova EK (2013). Polymorphism of RGS2 gene as genetic marker of schizophrenia risk and pharmacogenetic markers of the efficiency of typical neuroleptics. *Mol Biol* **47**(6): 814-820.

Glatt SJ, Everall IP, Kremen WS, Corbeil J, Sasik R, Khanlou N*, et al* (2005). Comparative gene expression analysis of blood and brain provides concurrent validation of SELENBP1 up-regulation in schizophrenia. *Proc Natl Acad Sci U S A* **102**(43): 15533-15538.

Glatt SJ, Stone WS, Nossova N, Liew C-C, Seidman LJ, Tsuang MT (2011). Similarities and differences in peripheral blood gene-expression signatures of individuals with schizophrenia and their first-degree biological relatives. *American journal of medical genetics Part B, Neuropsychiatric genetics : the official publication of the International Society of Psychiatric Genetics* **156B**(8): 869-887.

Gokhale A, Larimore J, Werner E, So L, Moreno-De-Luca A, Lese-Martin C, *et al* (2012). Quantitative Proteomic and Genetic Analyses of the Schizophrenia Susceptibility Factor Dysbindin Identify Novel Roles of the Biogenesis of Lysosome-Related Organelles Complex 1. *J Neurosci* **32**(11): 3697-3711.

Herberth M, Koethe D, Cheng TM, Krzyszton ND, Schoeffmann S, Guest PC, *et al* (2011). Impaired glycolytic response in peripheral blood mononuclear cells of first-onset antipsychotic-naive schizophrenia patients. *Mol Psychiatry* **16**(8): 848-859.

Hirayama-Kurogi M, Takizawa Y, Kunii Y, Matsumoto J, Wada A, Hino M, *et al* (2017). Downregulation of GNA13-ERK network in prefrontal cortex of schizophrenia brain identified by combined focused and targeted quantitative proteomics. *J Proteomics* **158**: 31-42.

Horiuchi Y, Kondo MA, Okada K, Takayanagi Y, Tanaka T, Ho T, *et al* (2016). Molecular signatures associated with cognitive deficits in schizophrenia: a study of biopsied olfactory neural epithelium. *TransPsychiatr* **6**(10): e915-e915.

Huang G, Osorio D, Guan J, Ji G, Cai JJ (2020). Overdispersed gene expression in schizophrenia. *NPJ schizophrenia* **6**(1): 9-9.

Jaffe AE, Straub RE, Shin JH, Tao R, Gao Y, Collado-Torres L, *et al* (2018). Developmental and genetic regulation of the human cortex transcriptome illuminate schizophrenia pathogenesis. *Nat Neurosci* **21**(8): 1117-1125.

Kumarasinghe N, Beveridge NJ, Gardiner E, Scott RJ, Yasawardene S, Perera A, *et al* (2013). Gene expression profiling in treatment-naive schizophrenia patients identifies abnormalities in biological pathways involving AKT1 that are corrected by antipsychotic medication. *Int J Neuropsychopharmacol*: 1-21.

Leirer DJ, Iyegbe CO, Di Forti M, Patel H, Carra E, Fraietta S, *et al* (2019). Differential gene expression analysis in blood of first episode psychosis patients. *Schizophr Res* **209**: 88-97.

Li Z, Chen J, Yu H, He L, Xu Y, Zhang D, *et al* (2017). Genome-wide association analysis identifies 30 new susceptibility loci for schizophrenia. *Nat Genet* **49**(11): 1576-1583.

Lopez-Figueroa AL, Norton CS, Lopez-Figueroa MO, Armellini-Dodel D, Burke S, Akil H, *et al* (2004). Serotonin 5-HT1A, 5-HT1B, and 5-HT2A receptor mRNA expression in subjects with major depression, bipolar disorder, and schizophrenia. *Biol Psychiatr* **55**(3): 225-233.

Maekawa M, Owada Y, Yoshikawa T (2011). Role of polyunsaturated fatty acids and fatty acid binding protein in the pathogenesis of schizophrenia. *Curr Pharm Des* **17**(2): 168-175.

Mead C-LR, Kuzyk MA, Moradian A, Wilson GM, Holt RA, Morin GB (2010). Cytosolic protein interactions of the schizophrenia susceptibility gene dysbindin. *J Neurochem* **113**(6): 1491-1503.

Mistry M, Gillis J, Pavlidis P (2013). Genome-wide expression profiling of schizophrenia using a large combined cohort. *Mol Psychiatry* **18**(2): 215-225.

Mizutani R, Saiga R, Takeuchi A, Uesugi K, Terada Y, Suzuki Y, *et al* (2019). Three-dimensional alteration of neurites in schizophrenia. *TransPsychiatr* **9**(1): 85.

Okayama T, Hashiguchi Y, Kikuyama H, Yoneda H, Kanazawa T (2018). Next-generation sequencing analysis of multiplex families with atypical psychosis. *TransPsychiatr* **8**(1): 221-221.

Schmitt A, Leonardi-Essmann F, Durrenberger PF, Wichert SP, Spanagel R, Arzberger T, *et al* (2012). Structural synaptic elements are differentially regulated in superior temporal cortex of schizophrenia patients. *Eur Arch Psychiatry Clin Neurosci* **262**(7): 565-577.

Shimamoto C, Ohnishi T, Maekawa M, Watanabe A, Ohba H, Arai R, *et al* (2014). Functional characterization of FABP3, 5 and 7 gene variants identified in schizophrenia and autism spectrum disorder and mouse behavioral studies. *Hum Mol Genet* **23**(24): 6495-6511.

Sivagnanasundaram S, Cosset B, Dedova I, Cordwell S, Matsumoto I (2007). Abnormal pathways in the genu of the corpus collosum in schizophrenia pathogenesis: a proteome study. *Proteomics – Clinical Applications* **1**(10): 1291-1305.

Watanabe A, Toyota T, Owada Y, Hayashi T, Iwayama Y, Matsumata M, *et al* (2007). Fabp7 maps to a quantitative trait locus for a schizophrenia endophenotype. *PLoS Biol* **5**(11): e297-e297.

Xia X, Ding M, Xuan J-F, Xing J-X, Pang H, Wang B-J, *et al* (2018). Polymorphisms in the human serotonin receptor 1B (HTR1B) gene are associated with schizophrenia: a case control study. *BMC Psychiatry* **18**(1): 303-303.

Zhang Y, You X, Li S, Long Q, Zhu Y, Teng Z, *et al* (2020). Peripheral Blood Leukocyte RNA-Seq Identifies a Set of Genes Related to Abnormal Psychomotor Behavior Characteristics in Patients with Schizophrenia. *Medical science monitor : international medical journal of experimental and clinical research* **26**: e922426-e922426.

Zhao Z, Xu J, Chen J, Kim S, Reimers M, Bacanu SA, *et al* (2015). Transcriptome sequencing and genome-wide association analyses reveal lysosomal function and actin cytoskeleton remodeling in schizophrenia and bipolar disorder. *Mol Psychiatry* **20**(5): 563-572.

Supplementary Note 1

Background

Recently, attempts to optimise transcriptomic data to maximise the true positive / false positive ratio has involved correction for a false discovery rate (Benjamini and Hochberg, 1995). Unfortunately, such a correction introduces an analysis bias as it has been established this approach has major problems in identifying true positive differences when cohort sizes are low (Pawitan et al., 2005; Tong and Zhao, 2008). Another approach to maximising the true positive / false positive ratio when analysing transcriptomic data was recommended by the Maqc Consortium (Maqc Consortium et al., 2006). Following the Maqc Consortium recommendations, data is first differentiated by being separated using a non-stringent group-wise P-value cutoff of 0.01. Differences that exceed that differentiator (i.e. $p < 0.01$) are further separated into accepted true positives when there is a fold change in expression of ≥ 20%. In our studies using human data we have shown that using the Maqc Consortium recommendations gives a true to false positive ratio of ≥ 90%. However, in the mouse it has been argued that true positive changes in gene expression are better defined using a significance level of < 0.01 in combination with a fold change in level of expression ≥ 50% (Chiu et al., 2013). Thus, we decided to compare outcomes from these three approaches to identifying significant changes in gene expression in muscarinic M1 receptor knockout (Chrm1-/-) and wild type (w/t) mice to establish the most representative approach to analysing our data.

Methodology: Data Analyses

All CEL files were imported into JMP Genomics 9.0 (SAS, Cary, NC, USA) and manipulated as described in the methods section. Differentially expressed genes were then identified using ANOVA (t-test) with and FDR of α of 0.01, the Maqc Consortium criteria or the more stringent

criteria used with mouse data. The outcome of these three analyses were compared to identify the approach that included the highest percentage of changes in gene expression that were detected using all three approaches.

Results

Comparing levels of gene expression between Chrm1$^{-/-}$ and w/t mice using $p < 0.01$ from an ANOVA using an FDR of $\alpha = 0.01$ resulted in 7,444 genes being called as having an altered level of RNA in the cortex of CHRM1$^{-/-}$ mice (Figure 1A). The Maqc Consortium criteria (p < 0.01 and fold changes in gene expression compared to w/t of ±≥ 0.2) called levels of 4,745 RNAs as having altered levels of expression in the cortex of Chrm1$^{-/-}$ mice. Finally, there were 1,695 RNAs with a significant difference at p < 0.01 between groups and a fold change of ≥± 0.5 in the cortex of the CHRM1$^{-/-}$ mice.

Comparing the different fold criteria to the FDR called data showed that 98% of the RNA called as changed in the cortex of the Chrm1$^{-/-}$ mouse by the Maqc Consortium criteria were also called as having changed levels of expression by ANOVA with FDR (Figure 1B). By comparison, 100% of the RNAs called as having changed levels at p < 0.01 and ≥± 0.5 were called as changed using ANOVA and FDR. As would be expected, all of the RNAs called as being different in the cortex of the Chrm1$^{-/-}$ mice at p < 0.01 and ≥± 0.5 were called as changed by the Maqc Consortium criteria.

Conclusion

To maximise the true to false positive ratio in our study, our reported analyses is based on true positive changes of gene expression being defined as at significance of p < 0.01 with a fold change of ≥± 0.5 criteria (Chiu et al., 2013).

References

Benjamini Y, Hochberg Y (1995) Controlling the false discovery rate: A practical and powerful approach to multiple testing. Journal of the Royal Statisitcal Society Series B 57:289-300.

Chiu Isaac M, Morimoto Emiko TA, Goodarzi H, Liao Jennifer T, O'Keeffe S, Phatnani Hemali P, Muratet M, Carroll Michael C, Levy S, Tavazoie S, Myers Richard M, Maniatis T (2013) A Neurodegeneration-Specific Gene-Expression Signature of Acutely Isolated Microglia from an Amyotrophic Lateral Sclerosis Mouse Model. Cell Reports 4:385-401.

Maqc Consortium et al. (2006) The MicroArray Quality Control (MAQC) project shows inter- and intraplatform reproducibility of gene expression measurements. Nat Biotechnol 24:1151-1161.

Pawitan Y, Michiels S, Koscielny S, Gusnanto A, Ploner A (2005) False discovery rate, sensitivity and sample size for microarray studies. Bioinformatics 21:3017-3024.

Tong T, Zhao H (2008) Practical guidelines for assessing power and false discovery rate for a fixed sample size in microarray experiments. Stat Med 27:1960-1972.

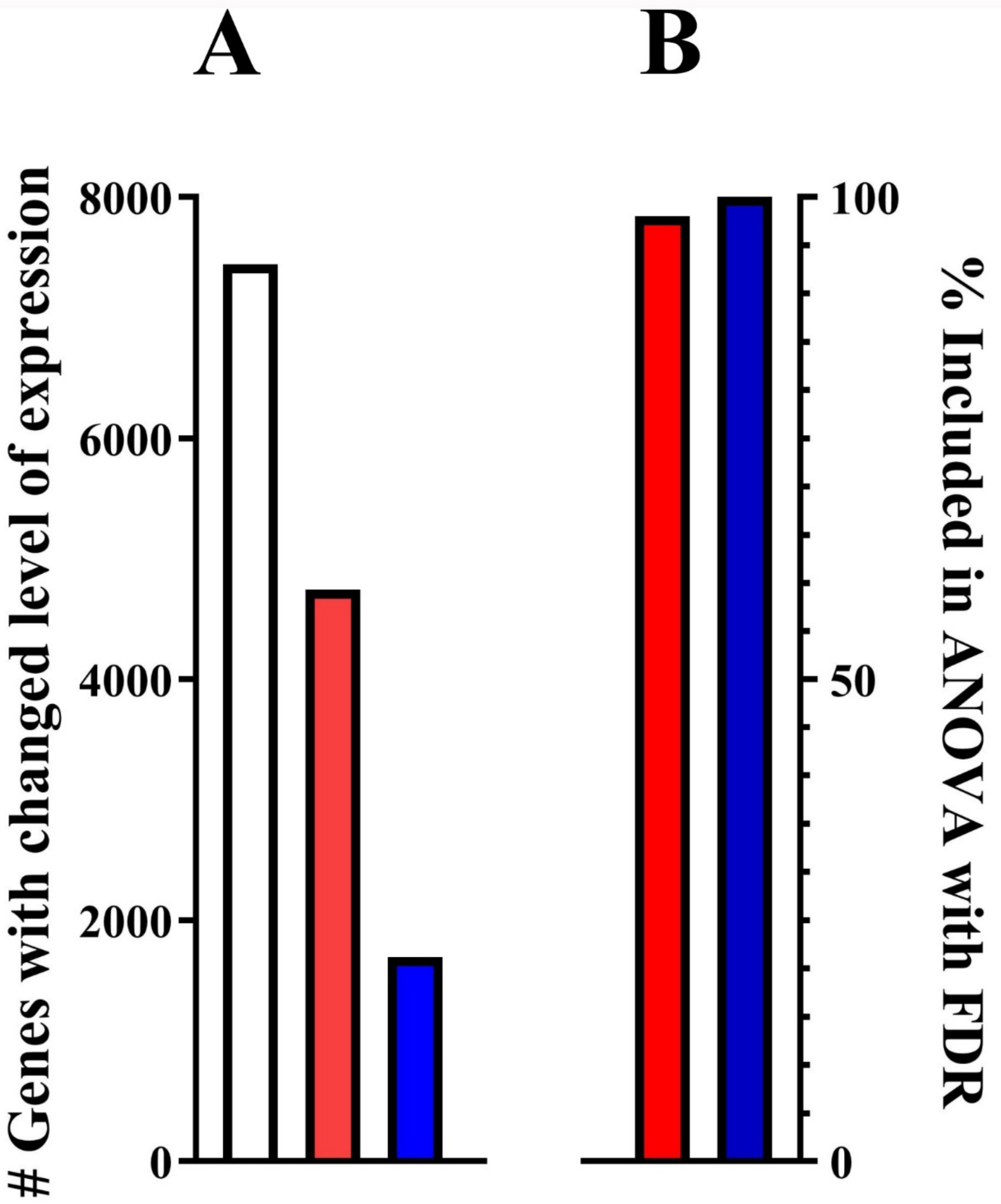

Figure 1: A: The number of genes called as true positives after comparing levels of RNA in the cortex of muscarinic M1 receptor (Chrm1$^{-/-}$) and wild type (w/t) using ANOVA with a false discovery rate (white column), the Maqc Consortium criteria ($p < 0.01$, fold $\geq\pm$ 0.2; red column) or criteria more appropriate to studies in mice ($p < 0.01$, fold $\geq\pm$ 0.5; blue column).

B: The per cent of levels of RNA in the cortex of muscarinic M1 receptor (Chrm1$^{-/-}$) mouse called as changed by the Maqc Consortium criteria ($p < 0.01$, fold $\geq\pm$ 0.2; red column) or criteria more appropriate to studies in mice ($p < 0.01$, fold $\geq\pm$ 0.5; blue column) compare to those called as changed using ANOVA with a false discovery rate.
